# Ultra-high resolution X-ray structure of orthorhombic bovine pancreatic Ribonuclease A at 100K

**DOI:** 10.1186/s13065-023-00959-6

**Published:** 2023-07-27

**Authors:** David R. Lisgarten, Rex A. Palmer, Jon B. Cooper, Claire E. Naylor, Rosemary C. Talbert, Brendan J. Howlin, John N. Lisgarten, Janez Konc, Shabir Najmudin, Carina M. C. Lobley

**Affiliations:** 1 Biomolecular Research Group, School of Psychology and Life Sciences, Canterbury Christ Church University, North Holmes Road, Canterbury Kent, CT1 1QU UK; 2 Department of Crystallography, Biochemical Sciences, Birkbeck College, Malet St., London, WC1E7HX UK; 3 Division of Medicine, UCL, Gower Street, London, WC1E 6BT UK; 4 Kisaco Research, 41a Maltby Street, London, SE1 3PA UK; 5 Biomolecular Research Group, School of Psychology and Life Sciences, Canterbury Christ Church University, North Holmes Road, Canterbury, Kent, CT1 1QU UK; 6 Chemical Sciences Division, Faculty of Health and Medical Sciences, University of Surrey, Surrey, Guildford, GU2 7HX UK; 7 School of Science, University of Greenwich (Medway Campus), Kent, Chatham Maritime, ME4 4TB UK; 8 National Institute of Chemistry, Hajdrihova 19, 1000 Ljubljana, Slovenia; 9 Randall Centre for Cell and Molecular Biophysics, Faculty of Life Sciences and Medicine, King’s College, 3rd Floor New Hunt’s House, London, SE1 1UL UK; 10 European Spallation Source ERIC Lund, Kalmar County, Sweden

**Keywords:** Ribonuclease, Orthorhombic, Ultra-high resolution, Cluster analysis and molecular dynamics

## Abstract

The crystal structure of orthorhombic Bovine Pancreatic Ribonuclease A has been determined to 0.85 Å resolution using low temperature, 100 K, synchrotron X-ray data collected at 16000 keV (λ = 0.77 Å). This is the first ultra-high-resolution structure of a native form of Ribonuclease A to be reported. Refinement carried out with anisotropic displacement parameters, stereochemical restraints, inclusion of H atoms in calculated positions, five ${\text{SO}}_{4}^{2 - }$ moieties, eleven ethanol molecules and 293 water molecules, converged with final R values of R1(Free) = 0.129 (4279 reflections) and R1 = 0.112 (85,346 reflections). The refined structure was deposited in the Protein Data Bank as structure 7p4r. Conserved waters, using four high resolution structures, have been investigated. Cluster analysis identified clusters of water molecules that are associated with the active site of Bovine Ribonuclease A. Particular attention has been paid to making detailed comparisons between the present structure and other high quality Bovine Pancreatic Ribonuclease A X-ray crystal structures with special reference to the deposited classic monoclinic structure 3RN3 Howlin et al. (Acta Crystallogr A 45:851–861, 1989). Detailed studies of various aspects of hydrogen bonding and conformation have been carried out with particular reference to active site residues Lys-1, Lys-7, Gln-11, His-12, Lys-41, Asn-44, Thr-45, Lys-66, His-119 and Ser-123. For the two histidine residues in the active site the initial electron density map gives a clear confirmation that the position of His-12 is very similar in the orthorhombic structure to that in 3RN3. In 3RN3 His-119 exhibited poor electron density which was modelled and refined as two distinct sites, A (65%) and B (35%) but with respect to His-119 in the present ultra-high resolution orthorhombic structure there is clear electron density which was modelled and refined as a single conformation distinct from either conformation A or B in 3RN3. Other points of interest include Serine-32 which is disordered at the end of the sidechain in the present orthorhombic form but has been modelled as a single form in 3RN3. Lysine-66: there is density indicating a possible conformation for this residue. However, the density is relatively weak, and the conformation is unclear. Three types of amino acid representation in the ultra-high resolution electron density are examined: (i) sharp with very clearly resolved features, for example Lys-37; (ii) well resolved but clearly divided into two conformations which are well behaved in the refinement, both having high quality geometry, for example Tyr-76; (iii) poor density and difficult or impossible to model, an example is Lys-31 for which density is missing except for Cβ. The side chains of Gln-11, His-12, Lys-41, Thr-45 and His-119 are generally recognised as being closely involved in the enzyme activity. It has also been suggested that Lys-7, Asp-44, Lys-66, Phe-120, Asp-121 and Ser-123 may also have possible roles in this mechanism. A molecular dynamics study on both structures has investigated the conformations of His-119 which was modelled as two conformations in 3RN3 but is observed to have a single clearly defined conformation in the present orthorhombic structure. MD has also been used to investigate Lys-31, Lys-41 and Ser32. The form of the Ribonuclease A enzyme used in both the present study and in 3RN3 (Howlin et al. in Acta Crystallogr A 45:851–861, 1989) includes a sulphate anion which occupies approximately the same location as the ${\text{PO}}_{4}^{2 - }$ phosphate group in protein nucleotide complexes (Borkakoti et al. in J Mol Biol 169:743–755, 1983). The present structure contains 5 ${\text{SO}}_{4}^{2 - }$ groups SO41151–SO41155 two of which, SO41152 and SO41153 are disordered, SO41152 being in the active site, and 11 EtOH molecules, EOH A 201–EOH A 211 all of which have good geometry. H atoms were built into the EtOH molecules geometrically. Illustrations of these features in the present structure are included here. The sulphates are presumably present in the material purchased for use in the present study. 293 water molecules are included in the present structure compared to 134 in 3RN3 (Howlin et al. in Acta Crystallogr A 45:851–861, 1989).

**Supplementary Information:**

The online version contains supplementary material available at 10.1186/s13065-023-00959-6.

## Introduction

**Fig. 1:**
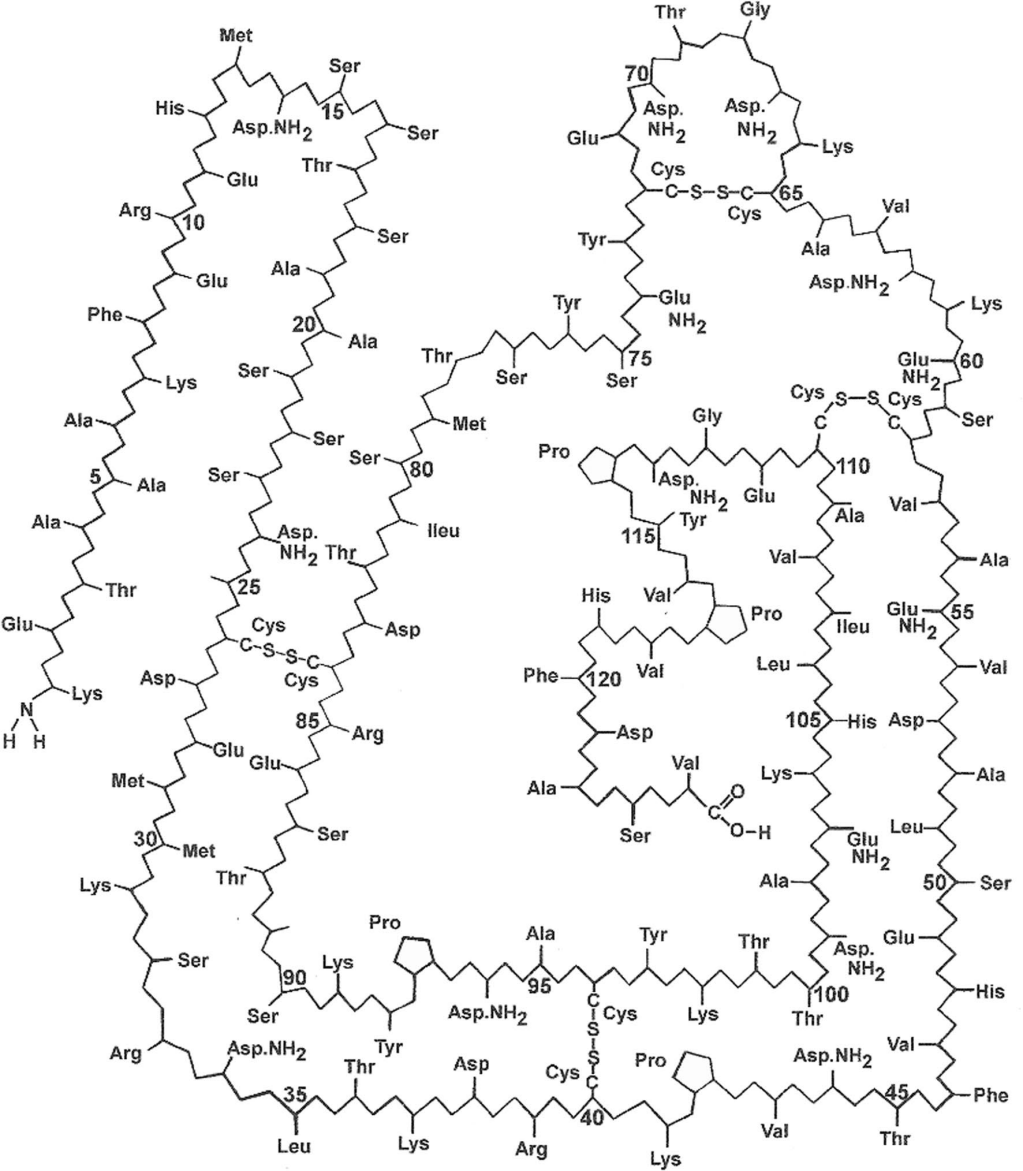
Bovine pancreatic ribonuclease A: Amino acid sequence. The side-chains of Gln-11, His-12, Lys-41, Thr-45 and His-119 are generally recognised as being closely involved in the enzyme activity. It has also been suggested that Lys-7, Asp-44, Lys-66, Phe-120, Asp-121 and Ser-123 may also have possible roles in this mechanism. This figure has no structural element apart from S–S Bridges

Ribonuclease A was one of the first enzymes to be studied by X-ray crystallography. Two independent structure analyses of Bovine Pancreatic Ribonuclease A were reported very early in the history of protein crystallography, neither of which included atomic coordinates based on their models of the structure: the first by Kartha et al. [1] and the second by Carlisle et al. [2]. A least-squares refinement of the bovine monoclinic structure at 1.45 Å resolution was undertaken in 1982 by Borkakoti et al. [3] initiated from atomic parameters published in 1982 by Wlodawer [4] from joint X-ray diffraction studies at 2.5 Å and neutron diffraction at 2.8 Å. Borkakoti et al. [3] were able to report the results of studies involving active site geometry, location of the sulphate molecule, hydrogen bonding, intermolecular contacts and solvent structure. In 1989 Howlin, Moss and Harris [5] using the 1.5 Å model of Borkakoti et al. [3] as their starting point, reported the results of segmented anisotropic refinement by the application of the rigid-body TLS model. These results were later deposited in the Protein Data Bank as structure 3RN3 [5] and are compared with the results of the present 0.85 Å refinement of the orthorhombic form. The amino acid sequence of Bovine Pancreatic Ribonuclease A is shown schematically in Fig. 1. Vergara et al. [6] have recently carried out studies on monoclinic RNase A but there is nothing to suggest that this paper includes high resolution studies of the RNase A structure either in the title of the paper or the PD Bank deposition. In 2002, Berisio et al. [7] published work based on the assumption that the diffraction pattern of protein crystals extending to atomic resolution guarantees a very accurate picture of the molecular structure and enables the study of subtle phenomena related to protein functionality. Six structures of Bovine Pancreatic Ribonuclease A at the pH* values 5.2, 5.9, 6.3, 7.1, 8.0 and 8.8 and at resolution limits in the range 1.05–1.15 Å have been refined. An overall description of the six structures and several aspects, mainly regarding pH-triggered conformational changes, are described here. Since subtle variations were expected, a thorough validation assessment of the six refined models was first carried out. Some stereochemical parameters, such as the N-Cα-C angle and the pyramidalization at the carbonyl C atoms, indicate that the standard target values and their weights typically used in refinement may need revision. A detailed comparison of the six structures has provided experimental evidence on the role of Lys41 in catalysis. Furthermore, insights are given into the structural effects related to the pH-dependent binding of a sulphate anion, which mimics the phosphate group of RNA, in the active site. Finally, the results support a number of thermodynamic and kinetic experimental data concerning the role of the disulphide bridge between Cys65 and Cys72 in the folding of RNase A. A discussion of this work in a subsequent paper on RNase A.

As described in “X-ray data processing for Ribonuclease A crystal” ultra-high-resolution data have now been collected for the native form of Orthorhombic Ribonuclease A. A 1.6 Å structure of an Orthorhombic form of Ribonuclease A was published by Dung and Bell in 1997 [8] which has the same space group as the present structure and similar unit cell parameters. A detailed comparison of the two orthorhombic structures will be published elsewhere. Here some of the features of the two orthorhombic structures are described for comparison and contrast. For the present ultra-high resolution structure maps have been generated and inspected at each of the three high resolution shells discussed. There are no significant differences between the data, consequently including the partial data to 0.85 Å has not had a detrimental effect and was included during the subsequent refinement stages. The use of a volatile mother liquor makes these crystals unsuitable for room temperature data collection and crystal dehydration using the HC1 device. Having obtained ultra-high-resolution data at 100 K there is no reason to pursue this further.

### Comparison of Structure RMSD’s

The structures from the radiation damage study by Caterino et al. [6] superimposed with the structure described in this paper (id 7P4R) give RMSD's of 0.65 and 0.67 Angstroms for the first and last structure they reported. In both cases, 124 residues were used to calculate the RMSD, and this is true for all the other subsequent superpositions reported unless otherwise stated. The active site residues (His 12, Lys 41 and His 119) were very similar except for bifurcation of the second His and the tip of the Lys residue. There were also differences in the loops from 36 to 40 and from 64 to 71 and some difference at Ser 21. Most of these are involved in peripheral active site contacts.

Looking at the structure with the isoaspartyl residue at 67 by Mazzarella et al. [7] and this is similar to the above comparison with more marked differences in the loop containing the mutation. The RMSD was therefore higher at 0.77 Angstroms for 122 equivalent residues.

The structures in the pH series (also by Mazzarella) from 1KF2 at pH 5.2 (RMSD 0.53 Angstroms) to 1kf5 at pH 7.1 (RMSD 0.54 A) and 1KF8 at pH 8.3 (RMSD 0.57 A). Of note is that these are all very much more similar to the structure reported in this present study than the others investigated.

The 7P4R structure is the only one that is orthorhombic, the rest being P21, so the similarity seems good despite the difference in space group.

## Materials and methods

### Materials

#### Ribonuclease A

Bovine Ribonuclease A type 2A was purchased from Sigma in the form of a white powder.

### Crystallization

**Fig. 2:**
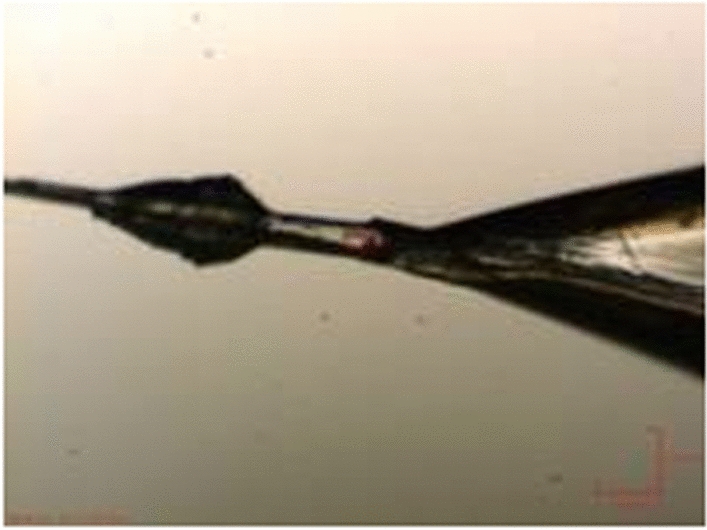
A crystal of RNase A mounted for data collection at Diamond

Crystals were obtained by dissolving approximately 100 mg of powdered Ribonuclease A in 1 ml of distilled water. After the powder had completely dissolved 2 ml of ice-cold absolute ethanol was added to the solution. This solution was then left at 4 degrees centigrade. Needle like crystals were obtained after a period of two weeks. The Ribonuclease A crystals were long needles – of the order of 500 µm long by about 50 µm by 50 µm. See Fig. 2.

### X-ray data collection

#### Diamond light source, MX beamline I02

Crystals of Ribonuclease A used for data collection were grown at 4 °C in absolute ethanol. The mother liquor from crystallisation was tested and behaves as a glass when flash cooled to 100 K. Consequently, no further cryoprotection was added to the crystals. A puck of 16 crystals were cooled and loaded to the sample changer. Each crystal was screened with three test shots, separated by 45° using 0.5 s exposure and 0.5° oscillation. Data were collected at 16,000 keV (0.77 Å) with the detector as close to the sample as possible (179.5 mm). Where diffraction data appeared to be of the order of 1.5 Å or better, data were collected. The EDNA strategy [9] was used to obtain a start angle and 180° of data were collected with 0.1° oscillation and 0.1 s exposure.

### X-ray data processing for Ribonuclease A crystal

Data were automatically integrated and scaled using Fast_dp and Xia2 on the beamline [10]. This highlighted the twelfth crystal as diffracting to the highest resolution. Manual processing of the data was carried out using XDS [11] to integrate and Aimless [12] to scale and merge intensities. The purpose of manual scaling was to optimise the included data to maximise the final resolution. Defining the resolution at which to cut off the data is a contentious issue. In this case the data extend to the corners of the detector, leading to low completeness in the outer shell. However, there was a signal in the outer shell at 0.85 Å resolution and consequently three.mtz files were available for analysis:

- Data scaled and merged to 0.85 Å.
- Data scaled and merged to 0.87 Å.
- Data scaled and merged to 0.89 Å.

The 0.85 Å data set was used throughout the subsequent analysis.

### Structure solution refinement

Molecular replacement was carried out using MOLREP [13] using the published structure 3RN3.pdb [5]. The final refinement was carried out using of REFMACS [14].

## Results

The space group is: P2_1_2_1_2_1_ (19) and the unit cell is: a = 43.9980 Å, b = 45.6970 Å, c = 51.8900 Å, α = 90°, β = 90^o^_,_ γ = 90°.

Model inspection and rebuilding were performed using the program COOT [15] and further isotropic refinement was carried out with the program PHENIX [16]. Water molecules were added at the end of refinement using the automated method provided in PHENIX [16]. Refinement of the orthorhombic Ribonuclease A crystal structure was carried iteratively using the program REFMAC5 interspersed with rounds of manual building and validation checks in COOT [15] linked to CCP4i2 suite [17]. Refinement was carried out with H atoms placed in the riding position and anisotropic temperature factors for all non-H atoms. Several residues were observed to have ordered or clear multiple conformations and were built into the structure with their relative occupancies summing to 1.0. The solvent and ligands were added towards the final steps. Final macromolecular structure model optimization for both was carried out on the PDB_REDO server [16]. At the end of the refinement the R factor and R free (all data) were 0.112 and 0.129, respectively. The program Dynarama [18] was used for structure validation. Inspection of the Ramachandran plot (see Additional file 1: Figs. S1 and S2) revealed that 96.77% of the residues are in favoured regions. The coordinates and data have been deposited in the Protein Data Bank, with identification code 7p4r. Statistics of refinement are summarized in Table 1.

**Table 1:** Refinement statistics for orthorhombic Ribonuclease A

Space group P2_1_2_1_2_1_ (19) Unit cell a = 43.9980 Å b = 45.6970 Å c = 51.8900 Å			
Data statistics at 0.85 Å initial refinement			
Aimless output			
	Overall	InnerShell	OuterShell
Low resolution limit	51.89	51.89	0.86
High resolution limit	0.85	4.66	0.85
Rmerge (within I+/I−)	0.076	0.051	0.697
Rmerge (all I+ and I−)	0.083	0.052	0.786
Rmeas (within I+ /I−)	0.091	0.059	0.893
Rmeas (all I + and I−)	0.090	0.057	0.908
Rpim (within I+/I−)	0.049	0.030	0.550
Rpim (all I+ and I−)	0.036	0.022	0.442
Rmerge in top intensity bin	0.034	–	–
Total number of observations	541,174	4209	11,184
Total number unique	87,934	668	2925
Mean((I)/sd(I))	13.2	48.6	1.6
Mn(I) half-set correlation CC(1/2)	0.998	0.996	0.402
Completeness	95.2	99.9	65.2
Multiplicity	6.2	6.3	3.8
Estimates of resolution limits: overall			
From half-dataset correlation CC(1/2)	> 0.50: limit = 0.87 Å		
From Mn(I/sd)	> 2.00: limit = 0.88 Å		

Molrep Output											
Nmon	RF	TF	theta	phi	chi	tx	ty	tz	TFcnt	wRfac	Score
1	1	1	107.65	176.59	95.81	0.257	0.209	0.170	19.11	0.532	0.595

Final refinement	
Refinement method	REFMACS [11]
Resolution high	0.85
Resolution low	34.29
Number of reflections (observed)	85,346
Number of reflections (R-free)	4279
Number of reflections (R-work)	79,223
R-Factor (R-work)	0.112
R-Factor (R-free)	0.129
RMSD bond lengths (Å)	0.020
RMSD bond angle (degree)	2.474
Number of non-hydrogen atoms used in refinement	
Protein atoms	1129
Heterogen atoms	68
Solvent atoms	293
Average isotropic B values (Å^2^)	
Main chain	4.01
Side chain	5.78
Whole chain	5.05

## Results

### Details of important structural features and selected residues

The present ultra-high-resolution structure of orthorhombic Bovine Pancreatic Ribonuclease A has revealed a number of interesting differences compared to the monoclinic structure 3RN3 [5]. In the first instance detailed studies of various aspects of hydrogen bonding have been carried out with particular reference to the following active site residues: Lys-1, Lys-7, Gln-11, His-12, Lys-41, Asn-44, Thr-45, Lys-66, His-119 and Ser-123. For the two histidine residues in the active site the initial electron density map gives clear confirmation that the position of His-12 is very similar in the orthorhombic structure to that in 3RN3 but with respect to His-119 in the ultra-high-resolution structure there is clear electron density, but the side chain occupies a single conformation different from either of the bifurcated sites A or B in the molecular replacement model 3RN3. Other points of interest include Serine-32 which has a double conformation in the present orthorhombic form but has been modelled as a single form in 3RN3. Lysine-66: There is good density with an extended major conformation and coiled minor conformation.

#### Ala-64–Cys-65–Lysine-66

**Fig. 3:**
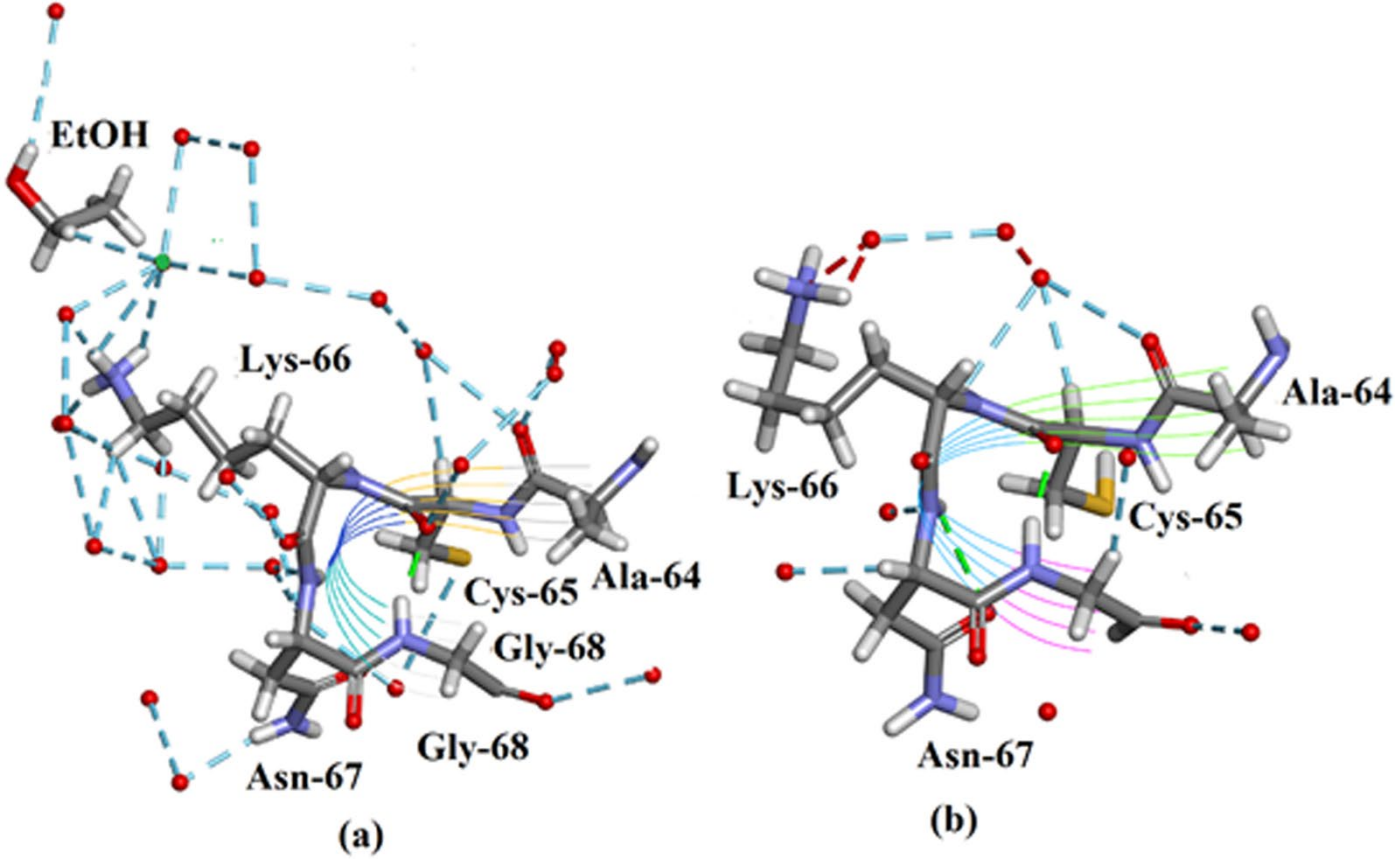
The sequence Ala-64–Cys-65–Lys-66: **a** Orthorhombic Ribonuclease A showing the extended major conformation of Lys66. **b** Monoclinic RNase A 3RN3 showing the single coiled side-chain conformation of Lys-66. Water oxygens are shown as red spheres; Hydrogen bonds are shown as broken lines; there is an ethanol EOH A 205 molecule linked in the orthorhombic form to Lys-66 side-chain via a water molecule; this ethanol is also linked to Asn-62 and Ser-123. Drawn with Biovia [19]. Note: it is thought that Lys-66 may have a role in the enzyme mechanism

In Fig. 3a The extended major conformation of Lys-66 in Orthorhombic Ribonuclease A is shown. There is good clear electron density for this residue which has been modelled largely as a single conformation but with a minor branch at the end. The X-ray model is divided into a major A part (67.5%) shown here and a minor B part (32.5%). A and B are only distinguishable at the end of the chain. The A chain shown here has an extended conformation. In Fig. 3b it is shown that for Monoclinic RNase A 3RN3 [5] there is a single coiled conformation of Lys-66. The three side chain torsion angles (deg) are: Orthorhombic: − 167.88, 178.29, − 162.23 extended; 3RN3 [5] Monoclinic: − 148.54, 9.22, -58.80 coiled.

Table 2 shows the main chain torsion angles φo and ψo for the sequence Ala-64–Cys-65–Lys-66. This indicates (reading down in the table) that the main chain conformations of 3RN3 [5] and Orthorhombic RNase A in this region are highly conserved. As seen in Fig. 3 however the side chain conformation of Lys-66 in the 0.85 Å resolution structure is extended but in the monoclinic 3RN3 [5] structure it is coiled.

**Table 2:** Main chain torsion angles φ° and ψ° for the sequence Ala-64–Cys-65–Lys-66

Cα	64	65	66	67	68
3RN3 φ^o^	− 76.17	− 71.77	− 62.61	− 87.51	101.53
Ortho φ^o^	− 72.78	− 68.04	− 64.82	− 90.53	90.13
3RN3 ψ^o^	161.80	143.99	161.90	− 26.52	5.50
Ortho ψ^o^	162.76	143.77	164.65	− 17.89	4.81

#### Lys-31–Ser-32–Arg-33

**Fig. 4:**
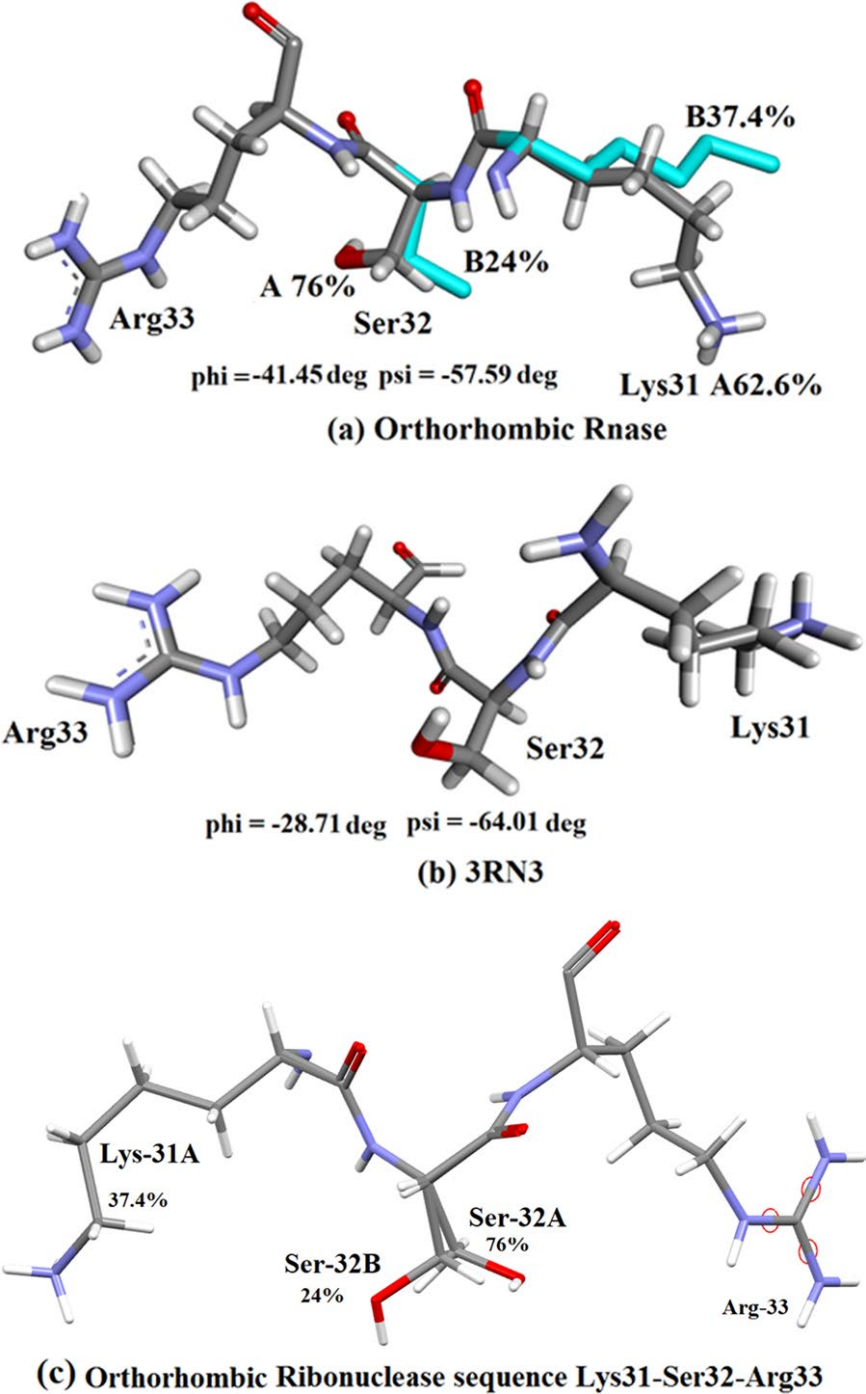
Conformation  of the sequence Lys-31-Ser32-Arg-33 at the end of alpha helix II (**a**) Orthorhombic RNase and (**b**) 3RN3. Details: **a** Orthorhombic RNase A Lys31-Ser32-Arg33 sequence showing the major (62.6%) conformation and minor (blue) (37.4%) conformation of Lys-31 and the major A conformation of Ser32 (76%) and minor conformation (blue) (24%) (Drawn with Biovia [19]; **b** Monoclinic RNase A 3RN3 [5] (Drawn with Biovia [19]); **c** Orthorhombic Ribonuclease A Lys-31A -Ser-32-Arg-33 sequence showing Ser-32 A (major 76%) and B (minor 24%) conformations. Drawn with MERCURY [20]. *Note*: The Major conformation of Orthorhombic Ser-32A is very similar to that of Ser-32 in 3RN3 [5]. **b** The main chain conformations of this sequence are characterised by significant differences as shown in Table 3 below

Lys-31 and Ser-32 have been modelled in the orthorhombic structure as each having 2 alternative conformations with occupancy A 62.6%, B = 37.4%, and A = 76%, B = 24% respectively, whereas only single conformations occur in 3RN3 [5], Fig. 4a, b. The minor side-chain conformations are shown in blue. The main-chain conformations are quite similar with φ = − 41.45°, ψ = − 57.59° in orthorhombic RNase A and φ = − 28.71, ψ = − 64.01 in 3RN3 [5] in each case within the accepted regions for α-helix formation. Figure 4c shows the two Ser-32 conformations in the orthorhombic structure.

**Table 3:** Main-chain torsion angles φ^o^ and ψ^o^ for residues Lys-31, Ser-32, Arg-33. This indicates (reading down in the table) that there are major differences in the main-chain conformations of 3RN3 [5] and Orthorhombic RNase A [OR] in this region

Cα	31	32	33
3RN3 φ^o^	− 71.42	− 64.01	− 88.83
OR φ^o^	− 173.52	− 176.22	169.41
3RN3 ψ^o^	50.80	− 28.71	6.50
OR ψ^o^	− 53.88	− 41.45	13.34

#### His-119 and ${\text{SO}}_{4}^{2 - }$

**Fig. 5:**
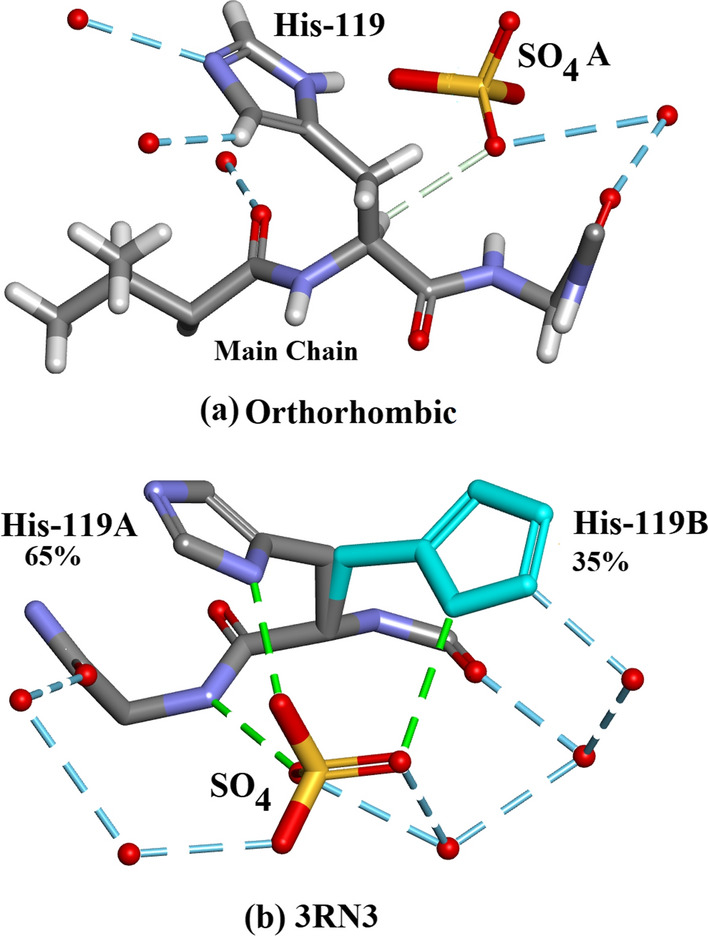
**a** Orthorhombic RNase A: His-119 occupies a single site. The ${\text{SO}}_{4}^{2 - }$ is disordered in this structure. Only the major ${\text{SO}}_{4}^{2 - }$ A site is shown. Drawn with Biovia [19]. **b** 3RN3 [5] His-119: major site A (65%) and minor site B (35%). The ${\text{SO}}_{4}^{2 - }$ is ordered. Drawn with Biovia [19]

In the ultra-high resolution Orthorhombic structure there is clear electron density for residue His-119 which occupies a single ordered site, but the sidechain occupies two different conformations in the molecular replacement model 3RN3 [5] site A 65% and B 35% (Fig. 5a, b). His-119 is also an important component of the active site described in “The active site details” below.

#### His-12

**Fig. 6:**
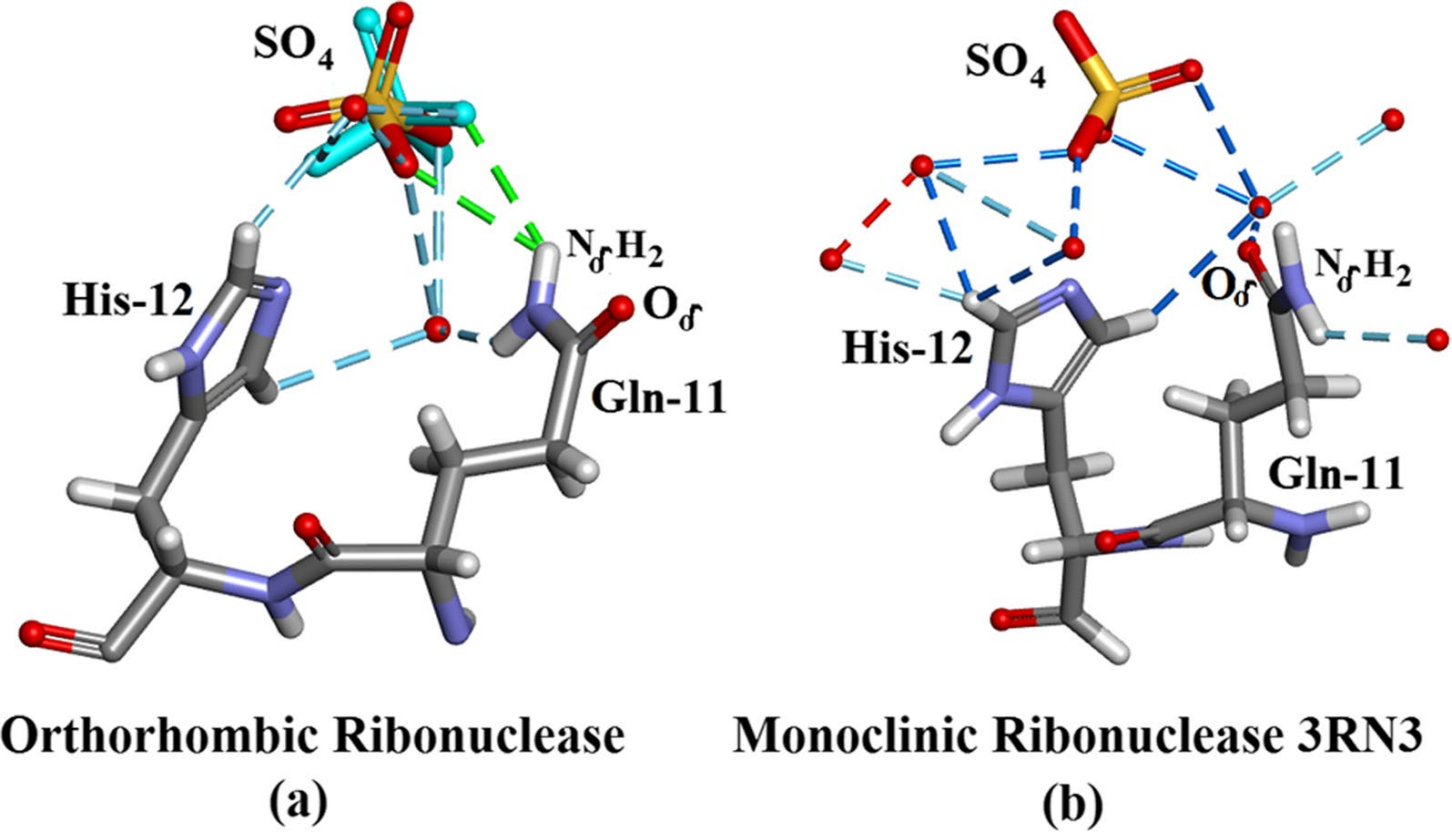
The active site of (**a**) Orthorhombic RNase A and (**b**) monoclinic RNase A 3RN3 [5] showing the ${\text{SO}}_{4}^{2 - }$, Gln-11 and His-12 groups and waters. H-bonds are dashed. In Orthorhombic RNase A His-12 H-bonds to the ${\text{SO}}_{4}^{2 - }$ group both directly and via a water molecule. Gln-11 H-bonds via the same water molecule and directly via the other NH group. In 3RN3 His-12 forms similar H-bonds and Gln-11 uses Oδ instead of Nδ. The conformations are similar. The ${\text{SO}}_{4}^{2 - }$ group is disordered into two groups in the orthorhombic structure. The electron density map gives clear definition of the position of His-12 in the orthorhombic model and is very close to that in 3RN3 (**b**). (Drawn with Biovia [19])

Partial views of the active sites of Orthorhombic RNase A are shown in Fig. 6a and 3RN3 in Fig. 6b. It is interesting to note that the conformation of Gln-11 appears to have changed (Fig. 6a, b). However, it would appear that the model selected for Gln-11 is responsible for this apparent difference as the conformation itself has not changed, see Fig. 6. His-12 is an important component of the active site residues, “The active site details”. In Orthorhombic RNase His-12 H-bonds to the ${\text{SO}}_{4}^{2 - }$ group both directly and via a water molecule. Gln-11 H-bonds via a water molecule and N_δ_H1 and directly via N_δ_H2. In 3RN3 [5] His-12 H-bonds to ${\text{SO}}_{4}^{2 - }$ via 2 water molecules and Gln-11 H-bonds to ${\text{SO}}_{4}^{2 - }$ by what appears to be O_δ_. However, the Gln-11 side-chain conformation appears to be unchanged between the two RNase A forms and H atoms have been modelled into 3RN3 with Biovia [19]. It is therefore quite likely that O_δ_ and N_δ_ in 3RN3 should be interchanged to make the two models of H-bonding between the two forms more compatible. This is shown in more detail in Fig. 6.

#### The active site details

**Fig. 7:**
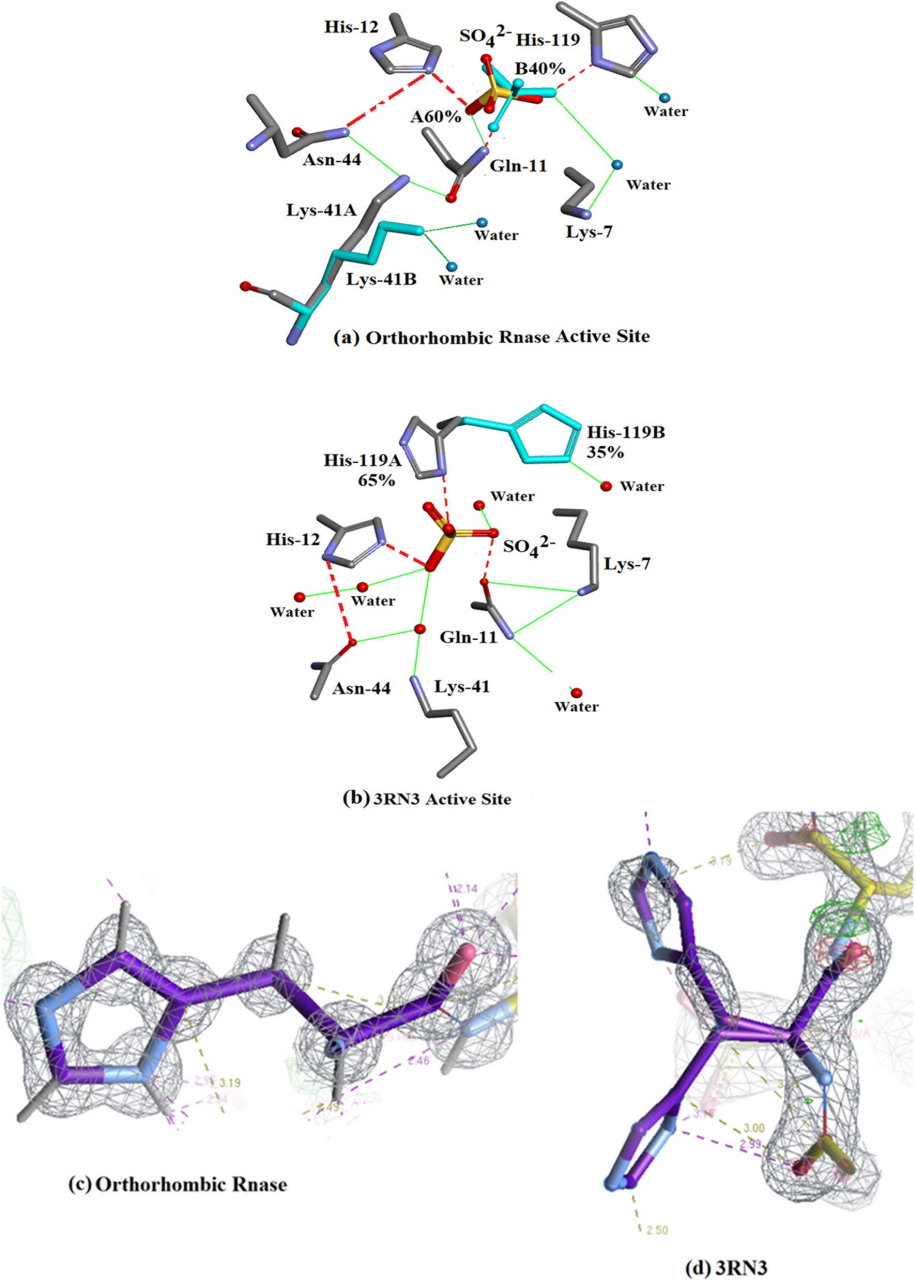
Structures of the active sites: (**a**) Orthorhombic RNase A. **b** 3RN3 [5]. **c** Clear single conformation of His-119 in Orthorhombic RNase A electron density. **d** Poor quality of electron density in His-119 3RN3 showing modelling of two conformations 65% and 35%

The form of the Ribonuclease A enzyme used in both the present study and in 3RN3 [5] includes a sulphate anion which occupies approximately the same location as the ${\text{PO}}_{4}^{2 - }$ phosphate group in protein nucleotide complexes [21]. The present structure contains 4 other ${\text{SO}}_{4}^{2 - }$ groups, two of which are disordered. Lys-7, Gln-11, His-12, Lys-41, Asn-44, and His-119 are considered to be important active site residues. In the orthorhombic structure Lys-7, Gln-11, His-12, Asn-44 and His-119 occupy single sites while Lys-41 is disordered into sites A and B and the ${\text{SO}}_{4}^{2 - }$ group into sites A (60%) and B (40%). In 3RN3 [5] Lys-7, Gln-11, His-12, Lys-41, Asn-44 and the ${\text{SO}}_{4}^{2 - }$ group occupy single sites while His-119 is disordered into sites A (65%) and B (35%). The locations of these residues are shown in Fig. 7. A number of water molecules also act to stabilize the structure as shown in Fig. 7. Further details of the interactions of the His-119 groups and the ${\text{SO}}_{4}^{2 - }$ moieties in the active sites can be seen in Fig. 7: (a) Shows how the single His-119 in orthorhombic Ribonuclease A links to the major ${\text{SO}}_{4}^{2 - }$ site and (b) shows how His-119 A and B in 3RN3 link to the single ${\text{SO}}_{4}^{2 - }$ site. There are 4 waters in the orthorhombic active site and six in 3RN3. Both active sites are stabilized by 11 hydrogen bonds.

As shown in Fig. 7a, the A site hydrogen bonds to His-12, and Gln-11 while the B-site hydrogen bonds to Gln-11, His-119 and a water molecule. His-12 is also stabilized by Asn-44, Lys-41A hydrogen bonds to Asn-44 and Gln-11 and Lys-41B are stabilized by 2 water molecules. Lys-7 hydrogen bonds to the same water molecule as ${\text{SO}}_{4}^{2 - }$B. Figure 7b shows 3RN3 [5]; His-119(A) hydrogen bonds to the ${\text{SO}}_{4}^{2 - }$ and His-119(B) hydrogen bonds to a water molecule. ${\text{SO}}_{4}^{2 - }$ is also stabilized by three water molecules and His-12, Gln-11 and His-119A. Asn-44 hydrogen bonds to both His-12, and Lys-41 via a water molecule and Lys-7 forms 2 H-bonds to Gln-11 which also H-bonds to a water molecule. Both of these assembled active sites seem to form quite stable units with no obvious reasons to explain the disorder of ${\text{SO}}_{4}^{2 - }$ in the orthorhombic structure or that of His-119 in 3RN3. Further details relevant to His-119 in 3RN3 are given in “His-119 and ${\text{SO}}_{4}^{2 - }$” and Fig. 4. As shown in Fig. 6, in Orthorhombic Ribonuclease A Gln11 Nε, not Oε, interacts with SO_4_, as reported by Borkakoti et al. [3] for Monoclinic Ribonuclease A. Drawn with Biovia [19]. Figure 7c shows clear single conformation of His-119 in Orthorhombic RNase A electron density. Figure 7d shows poor quality of electron density in His-119 3RN3 showing modelling of two conformations 65% and 35%.

### α-Helices and β-sheet structures in orthorhombic Ribonuclease A

#### α-Helices in orthorhombic Ribonuclease A

It was reported by Borkakoti et al. that in the monoclinic Ribonuclease A structure 3RN3 [5] there were three helix regions: α-helix I residues 3–13, α-helix II residues 24–33 and α-helix III residues 50–60; and three sheet regions βI residues 42–48, βII residues 71–92 and βIII residues 94–114.

An examination of these features in Orthorhombic Ribonuclease A follows.

### α-Helix regions

**Fig. 8:**
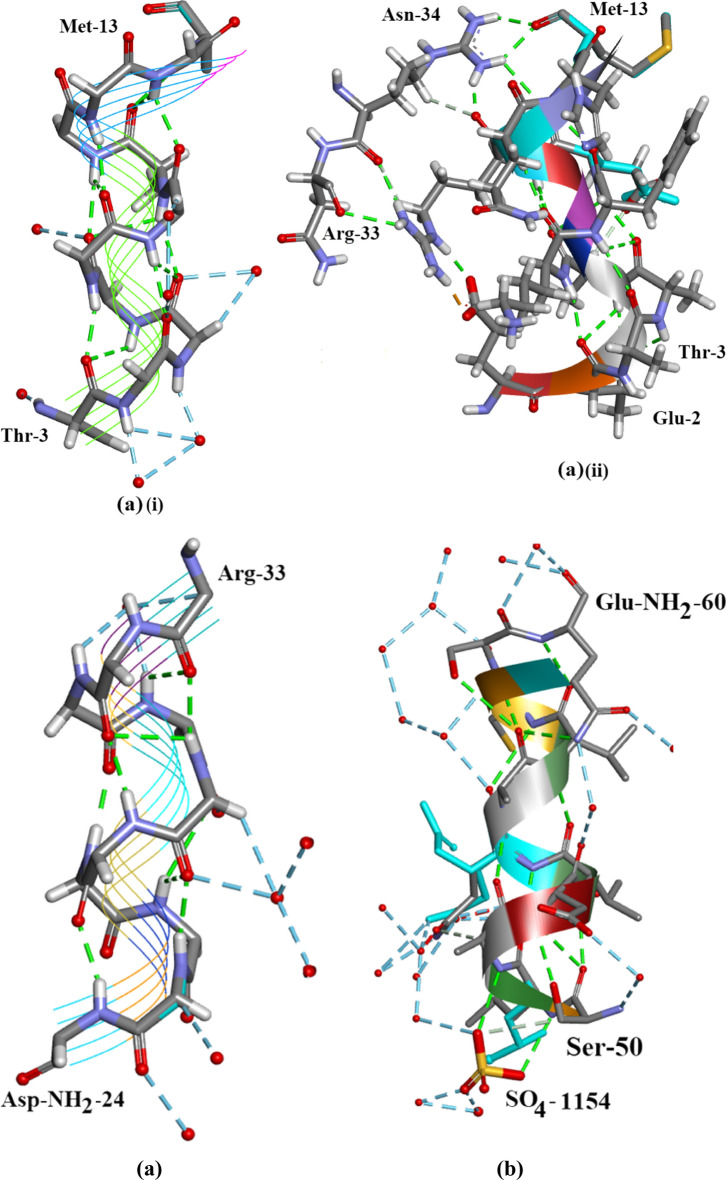

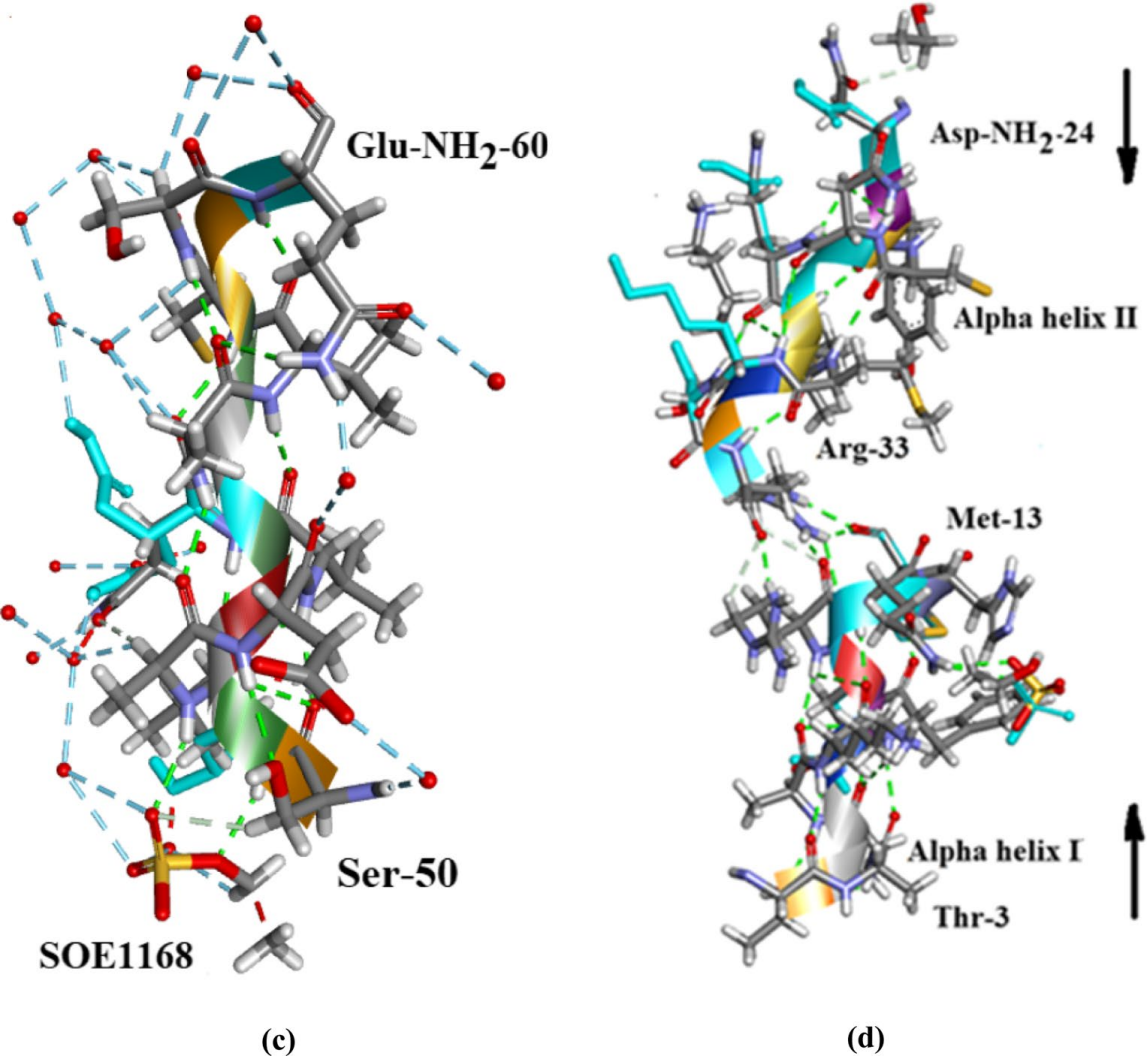
**a** (i) Alpha-helix II Orthorhombic Ribonuclease A. (ii) Alpha-helix II Orthorhombic Ribonuclease A showing Arg-33 and Asn-34 H-bonded at the side of the helix. **b** α-helix II covering residues Asp-NH_2_-24 to Arg-33. There are 2 waters and an EtOH on Asn-24, 2 waters on Asn-27 and Gln-28, 1 water on Met-30, Lys-31 and Ser-32 [not shown]. **c** α-helix III covering residues Ser-50 to Gln-NH2-60. SOE1168 hydrogen bonds to both Ser-50 and Leu-51. A number of water molecules H-bond to the residues along the helix [at least 15 can be seen in **c**]. Drawn with Biovia [19]. **d** The Hydrogen-bond link between α-helix I and α-helix II in Orthorhombic RNase A. The arrows indicate the direction of each helix which is opposed. There is an ethanol molecule EOH A 204 H-bonded to Asp-NH_2_-24 in α-helix II. Drawn with Biovia [19]

Helix Regions in Orthorhombic Ribonuclease A are shown in Fig. 8a i and ii, b, c and their Ramachandran plots in Additional file 1: Fig. S1, S2 and S3. Apart from the exceptions mentioned below the Ramachandran plots indicate that for all three helices the main chain (φ, ψ) values lie accurately within the region conventionally designated as α-helix. In the case of αI Gln-11 and His-12 (φ, ψ) values both lie outside this region, His-12 being the furthest away. It is interesting to note that α-helix I is linked to α-helix II via H-bonds between Met-13 and Arg-33, Fig. 8a (ii), d, the directions of the two helices being opposed.

#### β-sheet regions in orthorhombic Ribonuclease A

**Fig. 9:**
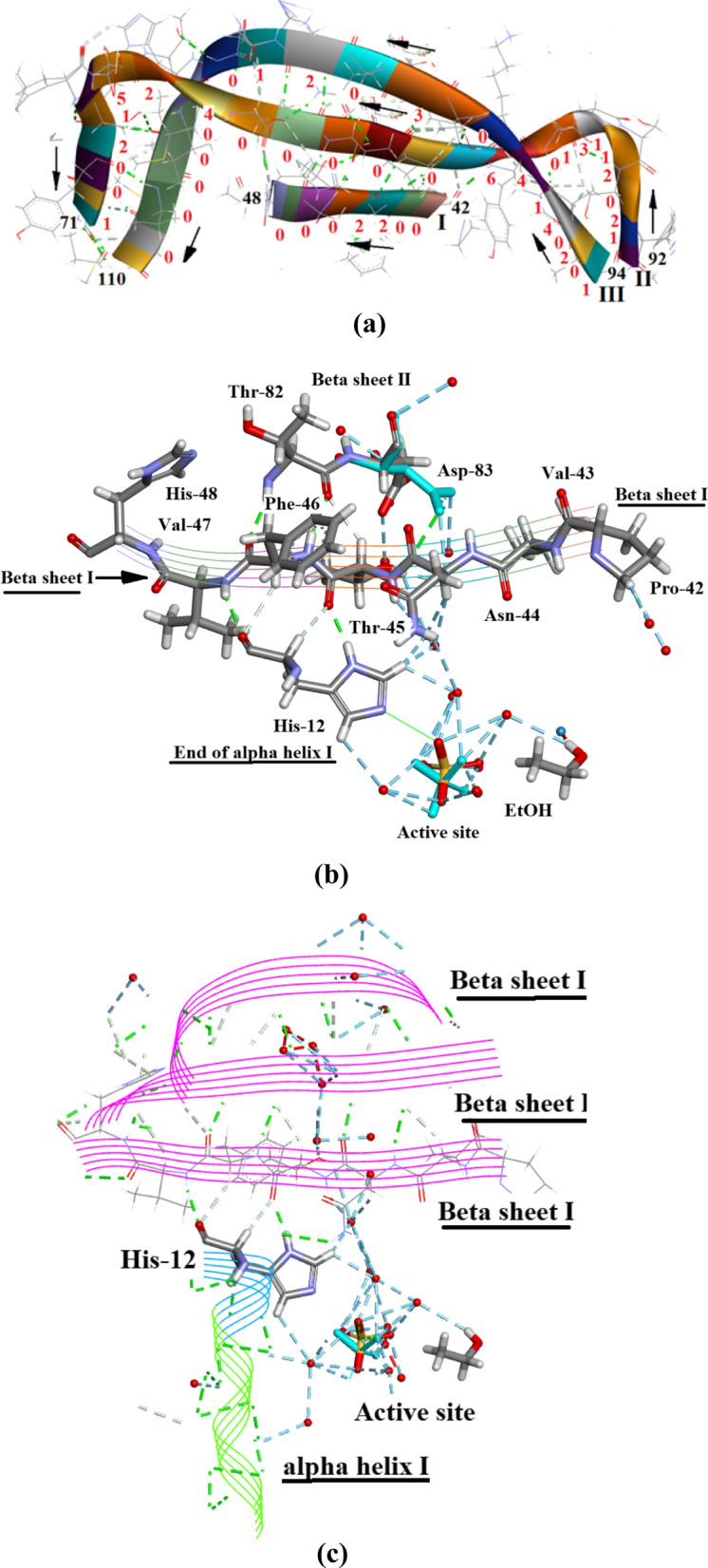
**a** The β- sheet regions in Orthorhombic RNase A. This shows that in the central region of the assembly the 3 β-sheets are approximately parallel. The red numbers indicate the number of waters at that site. There are several stretches with no water: notably 42–43 and, 46–48 in β-sheet I; 99, 100, 101, 102 in β-Sheet II. Drawn with Biovia [19]. **b** Shows how β-Sheet I is strongly linked to the active site through one of its residues His-12, (“The active site details”). The active site sulphate is SO41152 in the.pdb file and the EtOH is EOH A 203. **c** Shows how this association is strengthened further by Hydrogen bonding to β-Sheet II and β-Sheet III. The β-sheet structure and α-helix I thus make significant contributions to the stability of the active site. the EtOH is EOH A 203

The three β-sheet regions are shown in Fig. 9a and a combined Ramachandran plot in Additional file 1: Figures S4. The three regions cover residues (I) 42–48, (II) 71–92 and (III) 94–110. The arrows in Fig. 9a indicate that the β-sheet structural components are roughly parallel. The red numbers indicate the number of waters at a particular site. β-sheet I consisting of 6 residues has a total of 4 waters, β-sheet II has 27 waters for 21 residues and β-sheet III has 22 waters for 16 residues. The whole β-sheet region consisting of 43 residues attracts only 53 waters in contrast to the complete protein molecule of 124 residues and 300 waters. Of these 53 waters, 15 are linked to 4 residues in β-sheet II, and 14 are linked to 4 residues in β-sheet III. In other words, 29 waters are linked to only 9 residues and 34 waters are distributed between the remaining 24 residues. This means that it is possible that large regions in this assembly are almost waterless.

Figure 9b, c show how β-sheet I is strongly linked to the active site through one of its residues His-12, (“The active site details”) and Fig. 9c shows how this association is strengthened further by Hydrogen bonding to β-sheet II and β-sheet III. The β-sheet structure and α-helix I thus make significant contributions to the stability of the active site.

### Hydrogen bonding

#### Protein–protein and protein-solvent H-bonding in orthorhombic RNase A

In the crystallographic asymmetric unit, a total of 293 water molecule positions were assigned by stereochemical inspection and evaluation of the electron density displayed by WinCoot 0.7 [15]. These were included successfully in the refinement with anisotropic thermal displacement parameters. Water H atoms were fixed geometrically. Analysis of the hydrogen bonding properties of the water molecules and 124 peptide main-chain and side-chain atoms was carried out using Biovia in Accelerys Discovery Studio 3 [19]. The numbers of protein–protein and protein-solvent H-bonds are listed in Additional file 1: Table S2. For a given residue (sidechain or main-chain) the number of interactions varies from 0 to 6 and the number of protein–water interactions also varies from 0 to 6. A total of 299 protein–protein H-bonds was observed corresponding to 2–3 protein–protein H-bonds per residue and a total of 133 protein–water H-bonds were observed meaning that only about 40% of the water molecules take part in protein H-bonding or over 60% of the water molecules do not interact with a protein residue.

There are a number of other solvates present in the crystal structure: 5 ${\text{SO}}_{4}^{2 - }$ ions labelled in the.pdb file as SO41151, 1152, 1153, 1154 and 1155, all form H-bonds with side-chain atoms and 11 Ethanol molecules EOH A201 to EOH A 211 of which EOH A201, A202, A204 and A206 form close contacts with protein side-chain atoms while A 203, A 205 and A 208 are involved with waters.

Additional file 1: Table S1 lists the numbers of Protein–Protein and Protein Solvent Hydrogen bonds for each residue in Orthorhombic Ribonuclease A. Additional file 1: Table S2 summarises the number of residues with m Protein–Protein and n Protein-Water H-bonds. For example, reference to Additional file 1: Table S1 reveals that Ser-21 has neither of these types of bond (m = 0 and n = 0) and in all there are 10 residues with 1 Protein–Protein and 1 Protein-Water H-bond (see also Additional file 1: Figs S6, S7).

##### Examples of hydrogen bond formation

**Fig. 10:**
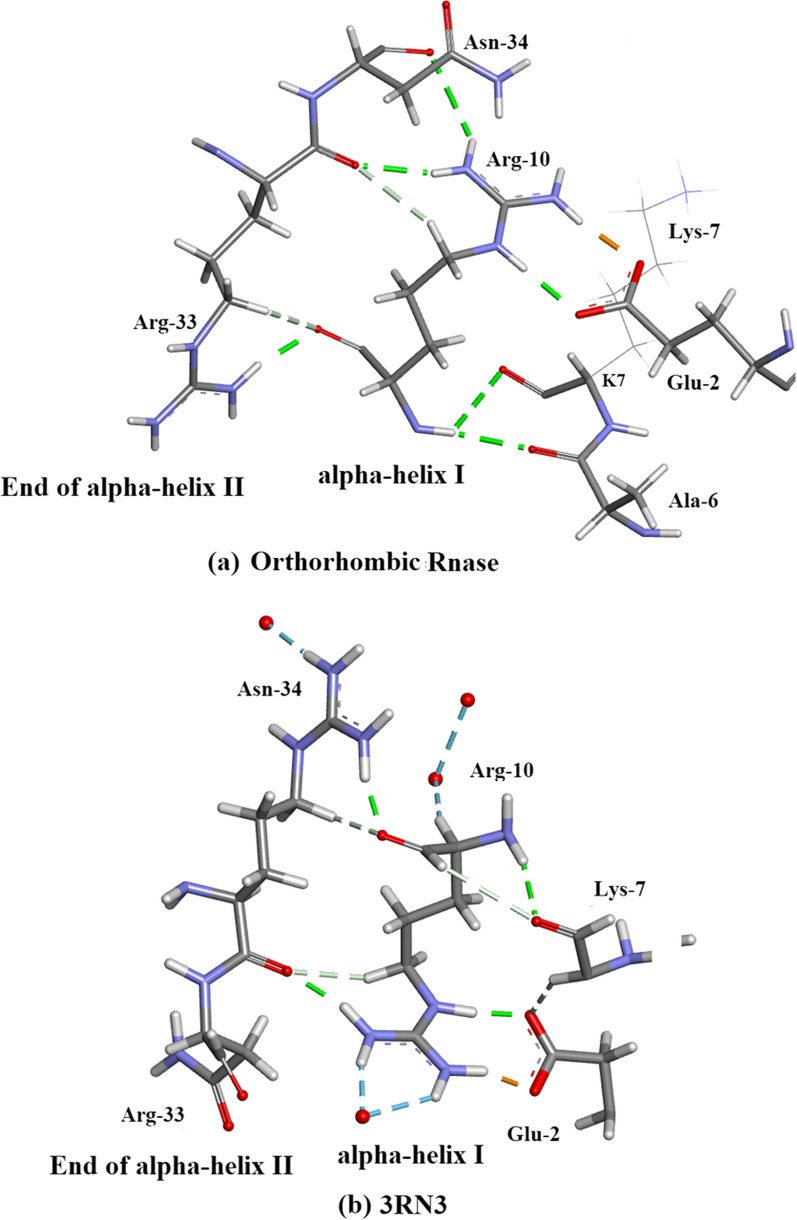
This shows some unusual H-bonding with respect to residue Arg-10 (**a**) in Orthorhombic RNase A and (ii) in 3RN3 [5]. Glu-2 forms 2 side-chain-side-chain H-bonds to Arg-10 in both structures. Ala-6 and Lys-7 each form a main-chain–main-chain H-bond to Arg-10. Asn-34 forms a main-chain-side-chain H-bond. Arg-33 forms a side-chain-main-chain H-bond and three main-chain-side-chain bonds. A total of nine inter residue H-bonds. Arg-10 which is a component residue towards the end of α-helix I (residues 3–13) interestingly does not form any H-bonds with solvent molecules. **b** In 3RN3 similar H-bonds form between Arg-33 and Asn-34 with Arg-10. Ala-6 does not interact with Arg-10 but both Glu-2 and Lys-7 do form H-bonds with Arg-10 which are not the same as those in Orthorhombic RNase A. In 3RN3 two water molecules form H-bonds with Arg-10 and one water molecule H-bonds to Orthorhombic RNase A Asn-34. Drawn with Biovia [19]

**Fig. 11:**
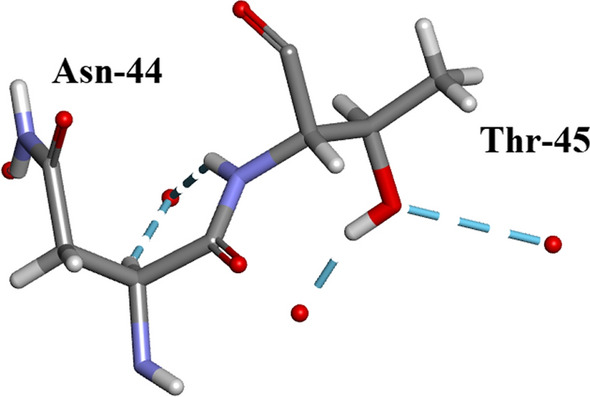
This shows the straightforward hydrogen bonding between Thr-45 and two water molecules

**Fig. 12:**
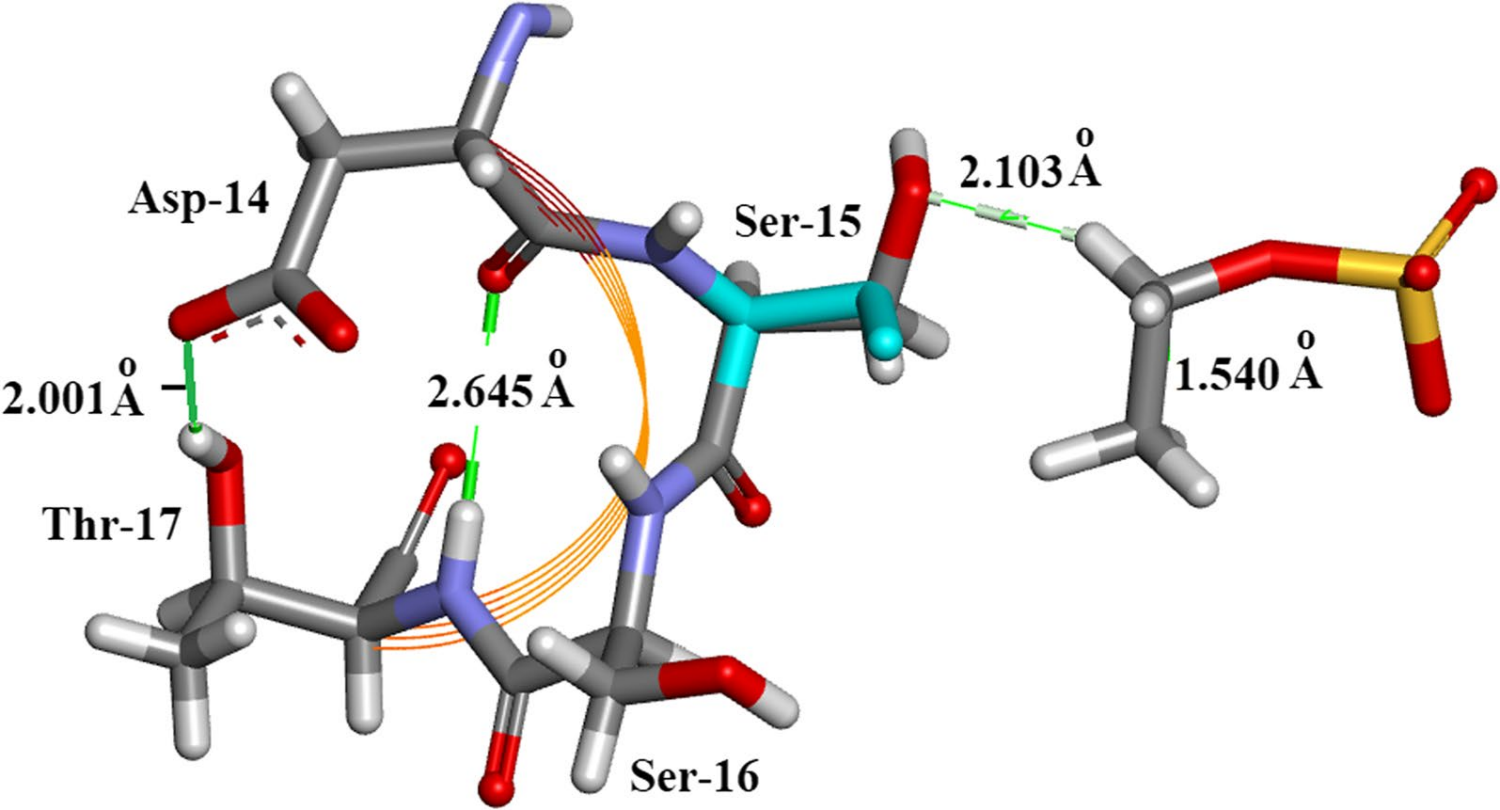
Orthorhombic RNase A. Residues Asp-14 to Thr-17 are located in α-helix I. H-bonds supporting the helix structure are shown. SOE1168 H-bonds to Ser-15

**Fig. 13:**
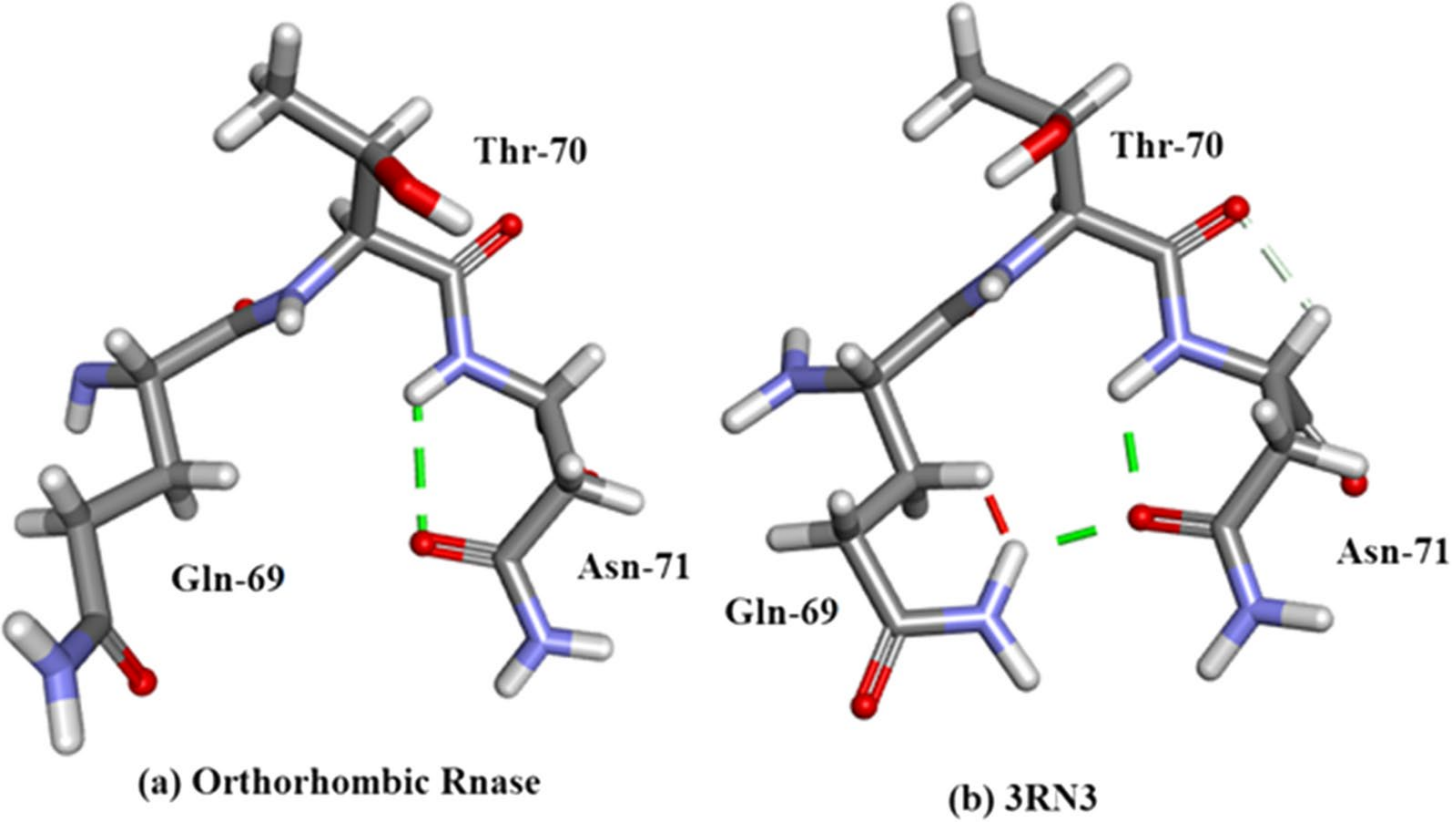
Orthorhombic RNase A. Gln-69 to Asn-71 compared to 3RN3

1. Arg-10
  Arg-10 forms H-bonds with 5 amino-acid residues: Glu-2, Ala-6, Lys-7, Arg-33 and Asn-34 as shown in Fig. 10a, b. There are no water-Arg-10 interactions. Arg-10 is a component of alpha-helix I (residues 3–13).
2. Thr-45 (Fig. 11). This an example of straightforward H-bonding. A water molecule H-bonds to two atoms in the Asn-44 side-chain and two other water molecules H-bond to the adjacent Thr-45 side-chain which is situated in β-sheet II.
3. Residues Asp-14 to Thr-17 are located in α-helix I Fig. 12. H-bonds supporting the helix structure are shown.
4. Orthorhombic RNase A the sequence Gln-69–Thr-70- Asn-71. In Orthorhombic RNase A Gln-69 and Asn-71 both have extended conformations. There is one Hydrogen bond which is main-chain (NH)–side-chain O=C in Asn-71 (Fig. 13a. In 3RN3 [5] Gln-69 now has a coiled conformation accompanied by 2 Hydrogen bonds: one side-chain-sidechain (self), one side-chain–sidechain (to Asn-71). Asn-71 has 2 further H-bonds not present in Orthorhombic RNase A, one side-chain-side-chain and the other H-bond main-chain–main-chain (Fig. 13b).

#### Water patches and voids in orthorhombic RNase A

**Fig. 14:**
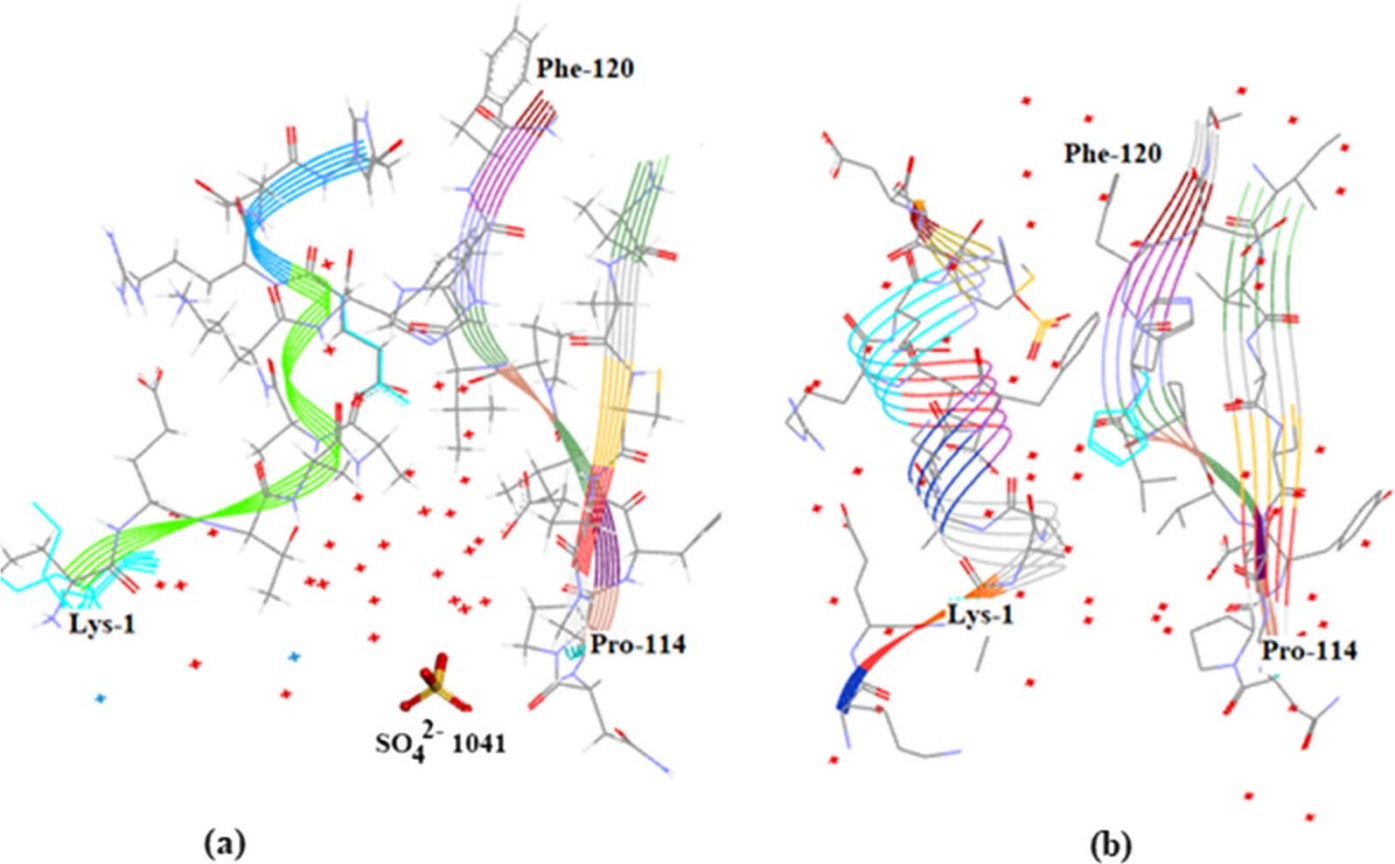
Water Patch at the C-Terminus entrance to the RNase A Structure: (**a**) Orthorhombic; (**b**) 3RN3

**Fig. 15:**
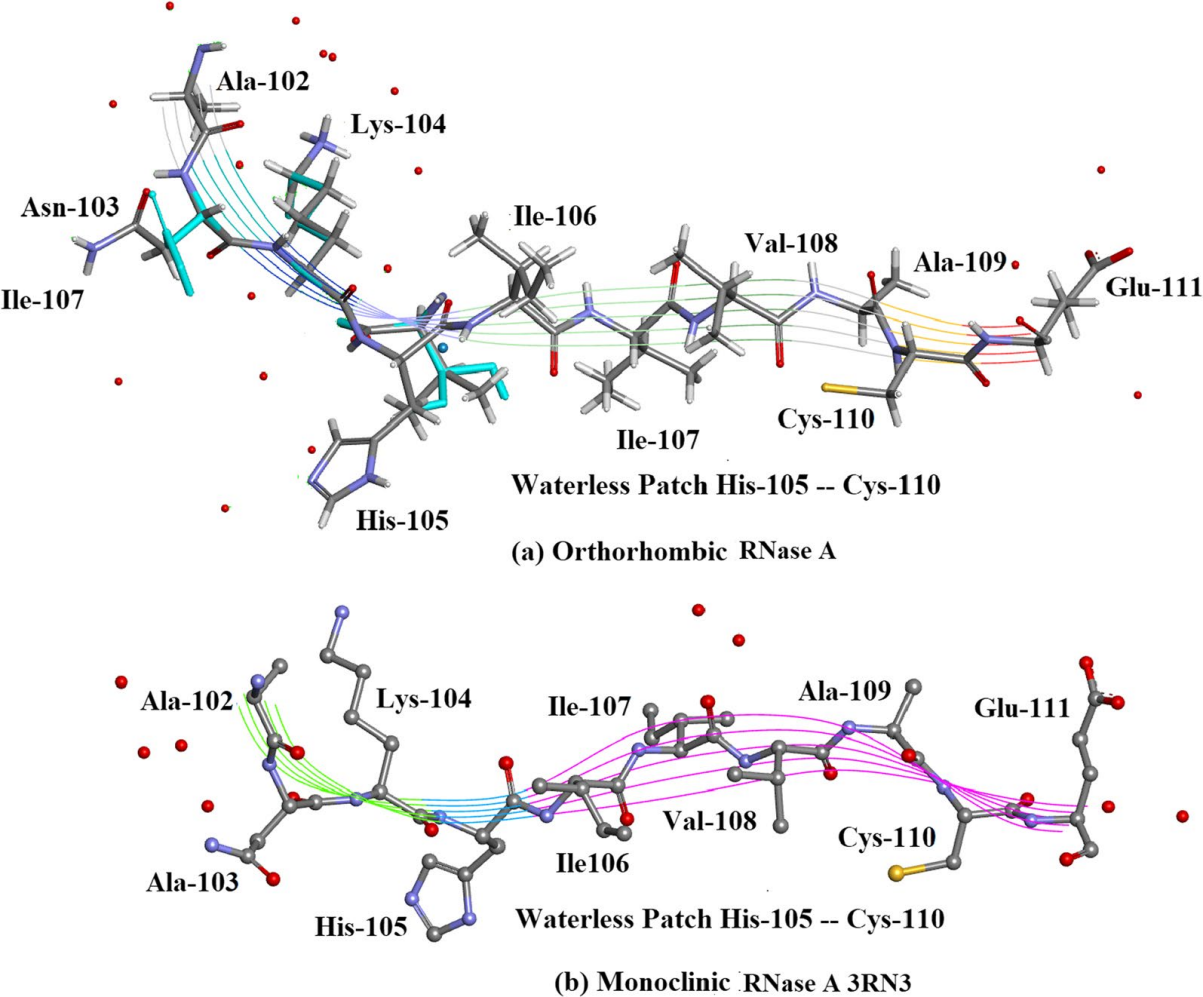
The Waterless Patch His-105–Cys-110

**Fig. 16:**
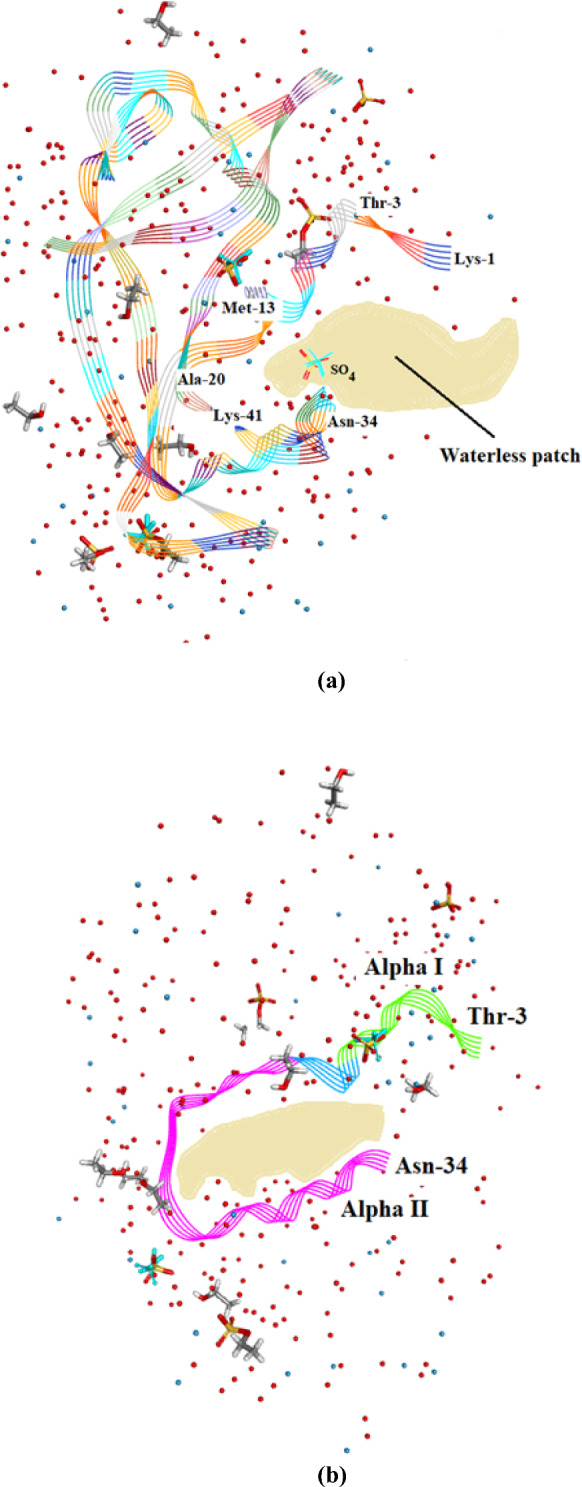
**a** The waterless patch I in Orthorhombic RNase A located between Lys-1 and Asn-34 (First view). **b**. A second view of this waterless patch including helices α-helix I and II. Other solvent molecules are shown in both **a** and **b**

1. The C-terminus–N-terminus region
  Figure 14a shows that there is a marked accumulation of water molecules in the region between the C-terminus Lys-1 and Pro-114 which is close to the N-terminus in Orthorhombic RNase A. SO_4_^2-^1041 is situated in this region. It is possible that this accumulation of water helps to stabilize the protein structure in this region. Figure 14b shows the corresponding region in 3RN3 [5] where a number of water molecules have been located but in keeping with the trend, in comparison between the two structures, in general the number of water molecules found is much less.
2. The Region His-105–Cys-110 Water Void
  Figure 15 Shows the region of the RNase A structures between Ala-102 and Glu-111 (a) Orthorhombic RNase A and (b) 3RN3. In both there is no sign of water in the regions His-105 to Cys-110. See Additional file 1: Table S1 where this void can be located. Other water voids are also evident on inspection
3. The Region Lys-1 to Asn-34 Water Void
  Figure 16a, b were produced by altering the position of the ribbon structure of Orthorhombic RNase A and examining the water content. There is a clear patch roughly between Lys-1 and Asn-34 and protruding towards the boundary of the protein structure which is void of water. To see this region more clearly would require looking at the crystal packing of the molecules. There may be a structural reason for this patch.

#### Numbers of protein–protein and protein solvent hydrogen bonds

Additional file 1: Table S1 lists for each residue of Orthorhombic RNase A the number of Protein–Protein Hydrogen bonds present and which residues are involved, the number of Protein Water Hydrogen bonds present and other Protein–Solvent Hydrogen bonds. In addition to 300 water molecules there are 3 ${\text{SO}}_{4}^{2 - }$ ions, 2 ethyl sulphate molecules (SOE) and 7 ethanol molecules per asymmetric unit. Some of these form H-bonds with protein residues. These are listed in Additional file 1: Table S1.

General Comments: The most protein–protein H-bonds (6) occurs for Ser-80. Several other residues have 5, these are Arg-10, His-12, Asp-14, Gln-55, Gln-60, Ser-80, and Gln-101. A large number of residues are void of protein–protein interaction. Ser-21 is void of any Hydrogen-bonding. There are examples of Hydrogen-bond formation in the examples given in previous sections. Additional file 1: Table S2 provides a complete listing of the numbers of residues with a given number of Hydrogen-bonds.

#### Salt bridges in orthorhombic RNase A

**Fig. 17:**
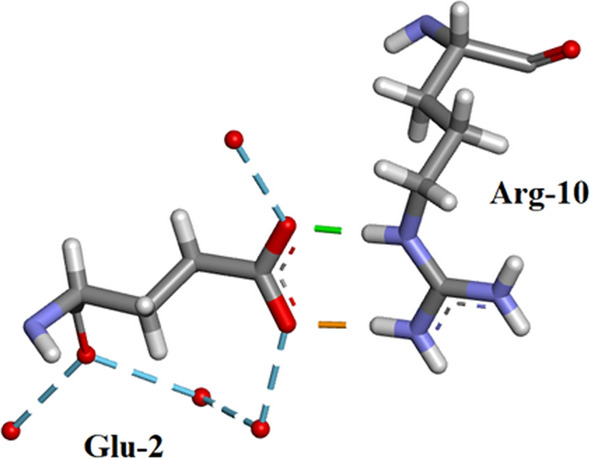
Shows the salt bridge 1 between Arg-10 and Glu-2. Some waters have been included which H-bond to Glu-2. Drawn with Biovia [19]

Residues involved in the five salt bridges observed in the Orthorhombic RNase A Structure are listed in Table 4 together with the corresponding bridge length. An example of a salt bridge between residues Arg-10 and Glu-2 is shown in Fig. 17.

**Table 4:** Orthorhombic RNase A residues involved in salt bridge formation

Salt bridge number	Residue 1	Residue 2	Distance in Å
1	Arg-10	Glu-2	1.992
2	Arg-33	Asp-14	3.151
3	Lys-41	SO_4_1152	2.916
4	Lys-66	Asp-121	2.420
5	Arg-85	Asp-83	2.236

### Survey of the peptide side chain electron density and conformations

#### Peptide side chain electron density and conformations in orthorhombic RNase A and 3RN3

It is well known that ultra-high resolution protein structures derived from X-ray diffraction data using cryo-cooled crystals often reveal amino acid residues which display more than a single ordered conformation. See for example Smith et al. [22], Addlagatta et al. [23] and Lisgarten et al. [24]. When such effects are observed it is possible that the use of these harsh high speed experimental conditions have both caused and allowed these alternative structures to be captured for detailed examination. It is also possible that such alternative conformations may have a bearing on the biological activity of the protein.

##### Properties of the electron density for orthorhombic RNase A and 3RN3

**Fig. 18:**
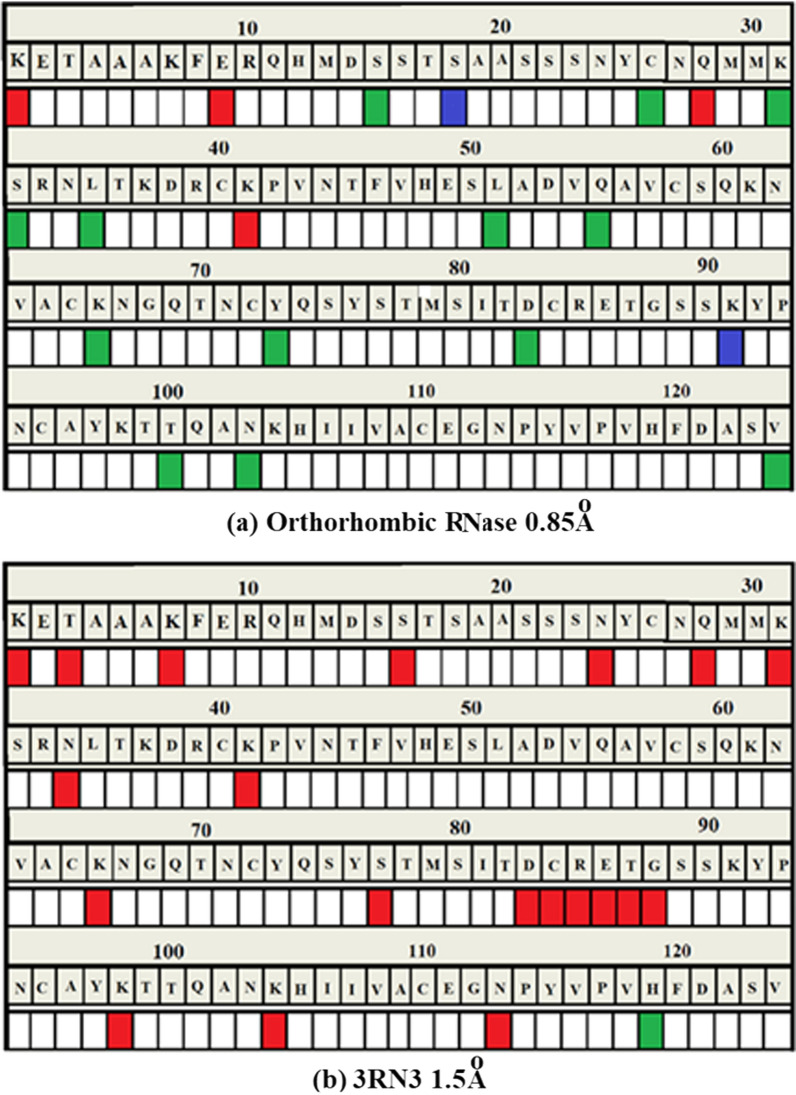
Analysis of the correspondence of amino acid modelling and electron density quality in (**a**) Orthorhombic RNase A and (**b**) 3RN3 [5]. Colour codes: White = Excellent quality electron density with minimal problems for modelling a clear single conformation; Green = Clear electron density with two distinct conformations modelled; Red = Very weak or missing electron density: Blue = Disordered

Properties of the electron density for Orthorhombic RNase A and 3RN3 [5] are summarised in colour code in Fig. 18a, b and in further detail in Additional file 1: Table S3.

##### Orthorhombic RNase A

**Fig. 19:**
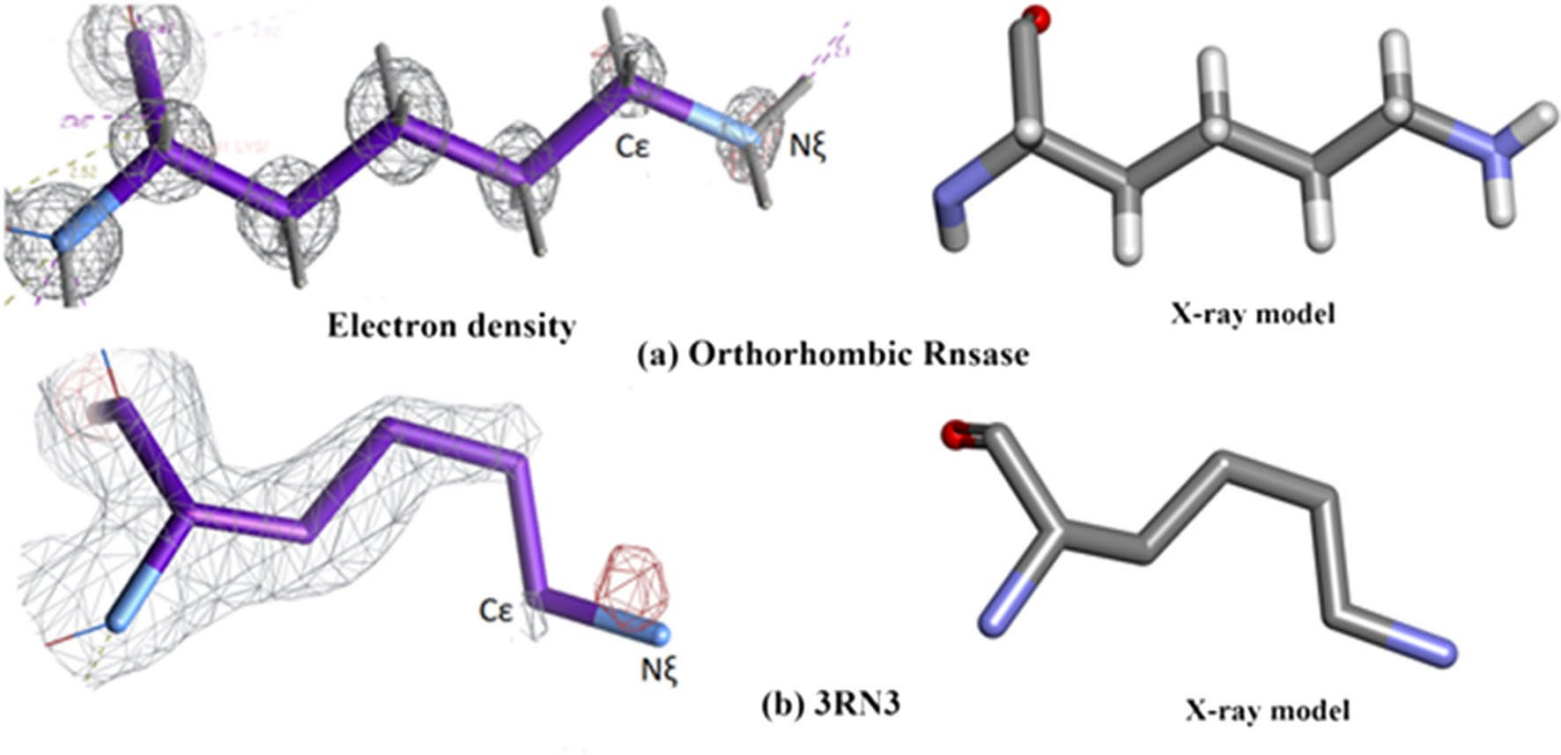
This shows the comparison of the electron density between Orthorhombic Rnase and 3RN3 for residue Lysine-61

The electron density of Orthorhombic RNase A is of very high quality (mainly white in Fig. 18a) with few problems associated with fitting the amino acid residue structures. Thirteen residues were modelled with complete or partial doubling (Green), but otherwise having very good electron density. Only two residues are noted as being disordered (Blue), Ser-18 and Cys-26. There are four residues with missing density (Red). Lys-91 has very poor density in both structures (Fig. 19).

##### 3RN3

As is to be expected the overall quality of electron density in the 3RN3 [5] structure is much lower than that of Orthorhombic RNase A. One residue, His-119, has a double conformation (Green). Twenty-two residues are designated Red, having missing density. The rest are categorised as White, having minimal problems.

##### The active site in orthorhombic RNase A in light of the electron density

As discussed previously the following residues have been recognised as the most likely to be involved in the Active Site Mechanism: Lys-1 (R), Lys-7 (W), Gln-11 (W), His-12 (W), Lys-41(R), Asn-44 (W), Thr-45 (W), Lys-66(G), His-119 (W), and Ser-123 (G). In view of their electron density properties, it seems justified to omit from this list: Lys-1 (R), Lys-41(R), Lys-66(G) and Ser-123 (G). The list then becomes: Lys-7 (W), Gln-11 (W), His-12 (W), Asn-44 (W), Thr-45 (W), and His-119 (W) together with the ${\text{SO}}_{4}^{2 - }$ group disordered into sites A (60%) and B (40%).

##### Overall comments

Two of the residues designated red, Fig. 18, are common to both structures, having missing density or disorder. These are Lys-1 and Gln-28. In Orthorhombic RNase A there are 4 Lysine residues which are either red or green as opposed to 9 in 3RN3 [5]. Lys-61 has complete and clear electron density in the case of Orthorhombic RNase A as shown in Fig. 19a whereas the electron density plot for 3RN3, Fig. 19b, shows Cε and Nξ to be absent. In Orthorhombic RNase A the sidechain of Lys-61 is fully extended whereas in 3RN3 it is coiled.

**Fig. 20:**
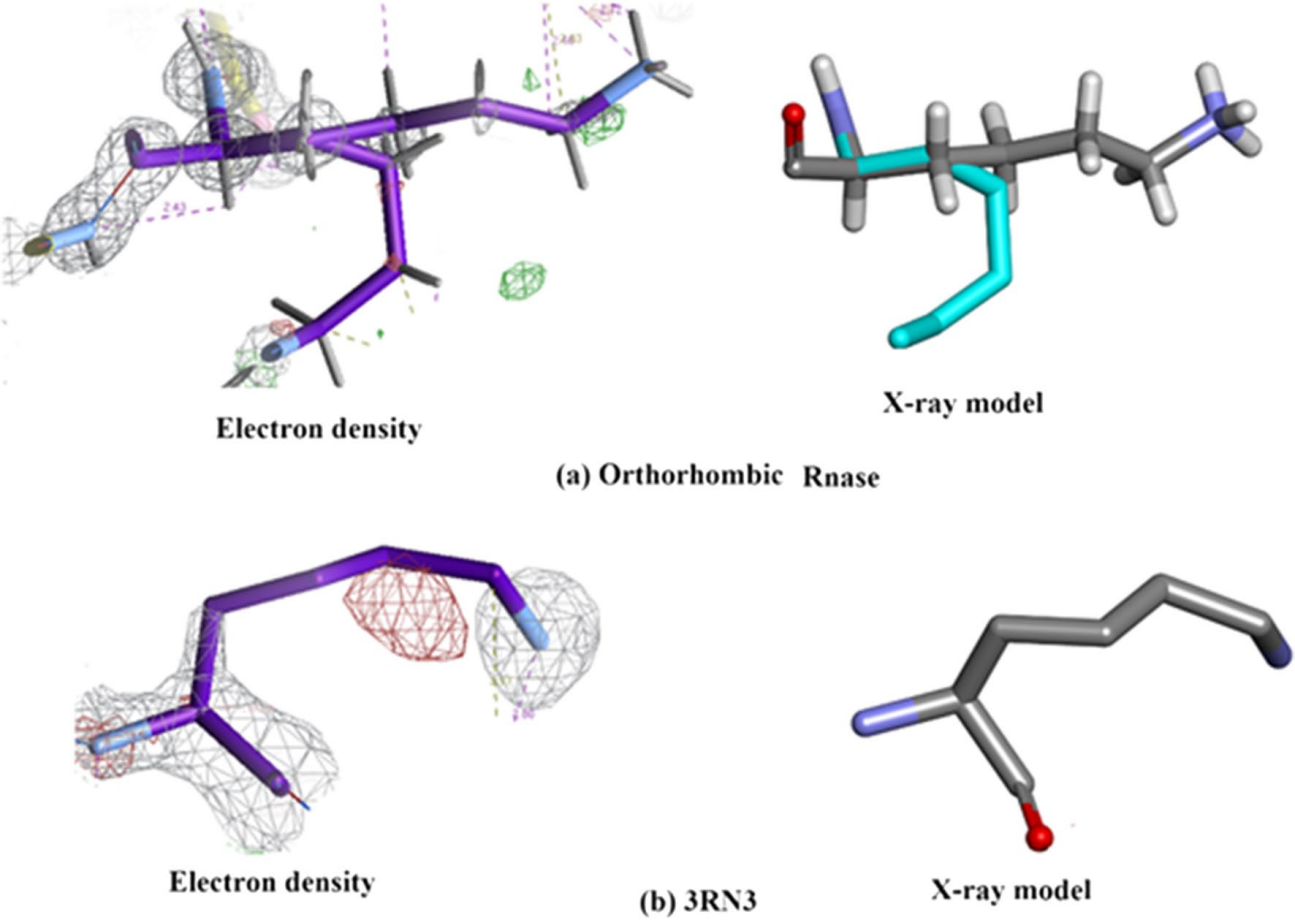
This shows the comparison of the electron density between Orthorhombic Rnase and 3RN3 for residue Lysine-91

In the case of Lys-91, Fig. 20a, b, the electron density for both the structures is weak making it difficult to model this part of the structure into the X-ray model. Fortunately, this is perhaps the only example of its kind in this study which gives so many problems.

It may be possible to rationalise these differences for example via Molecular Dynamics calculations.

##### Crystal packing

The molecular structure of Orthorhombic RNase A has been compared with that of the monoclinic 3RN3. It is also of interest to compare the crystal packing in both. Differences in orientations of the proteins appear as differences in protein–protein interactions. The interatomic interactions with neighbouring protein molecules have been studied for both the orthorhombic structures 7P4R and the monoclinic structure 3RN3 using PDBe PISA v1.52 [20/10/2014] on the EBI Web server. The results can be found in Additional file 1: Table S4.

### ${\text{SO}}_{4}^{2 - }$ moieties and solvated ethanol molecules in orthorhombic RNase A

**Fig. 21:**
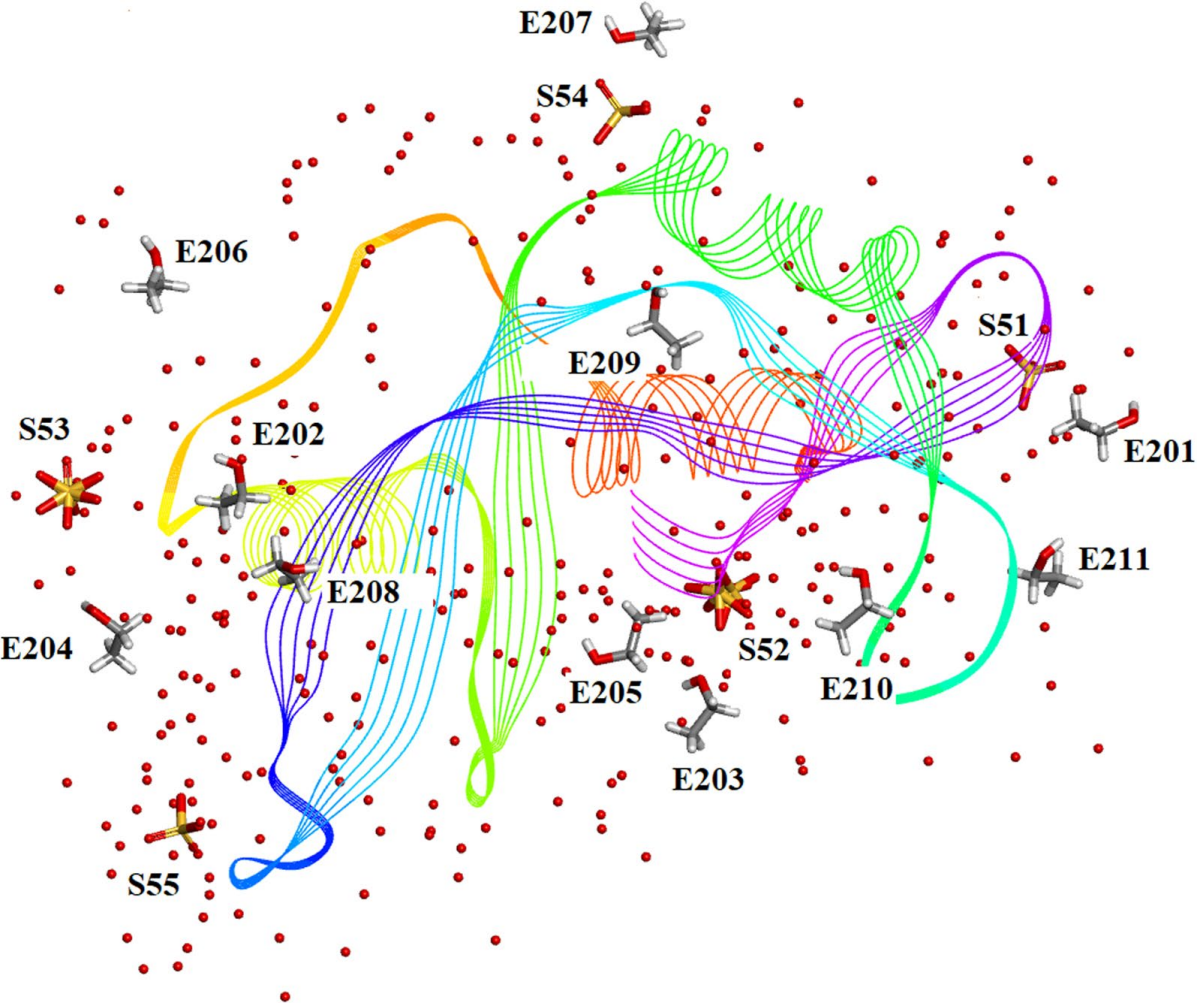
The distribution of Ethanol and Sulphate molecules in Orthorhombic Ribonuclease A Key: S51 is SO41151 etc. and E201 is EOH A201 etc. SO41152 is the active site sulphate and is disordered in two parts A (60%) and B (40%). SO41153 is also disordered in two parts A (50%) and B (50%). The ethanol molecules all ordered with good geometry. Both the sulphates and ethanol molecules are largely located in the external regions of the molecule

**Fig. 22:**
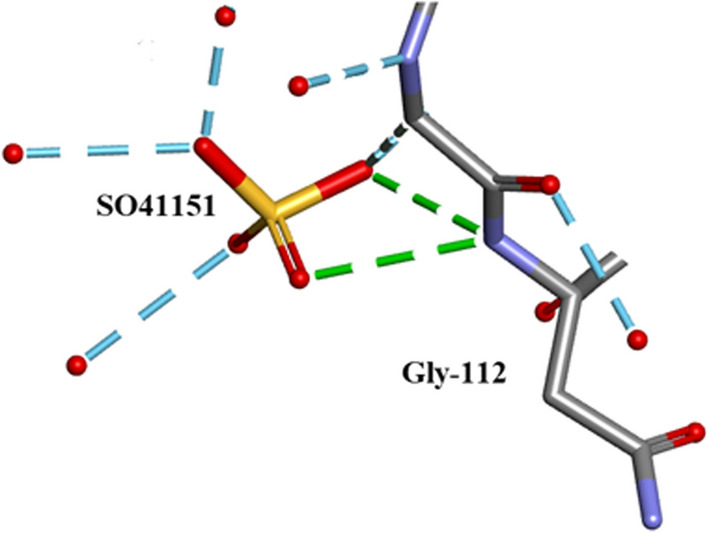
SO41151 links to Gly-112 and several waters

**Fig. 23:**
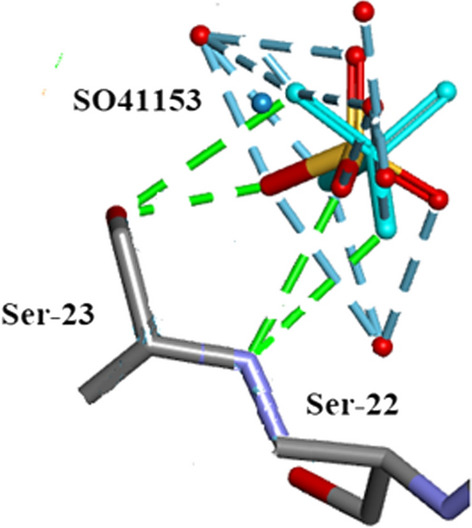
SO41153 Hydrogen Bonds to Ser-22 and Ser-23 and several waters

**Fig. 24:**
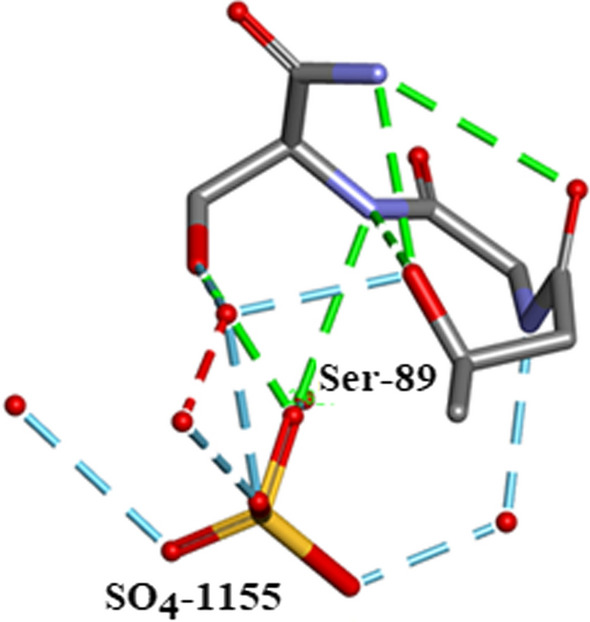
SO41155 forms a complex network with Ser-89 and several waters.

**Fig. 25:**
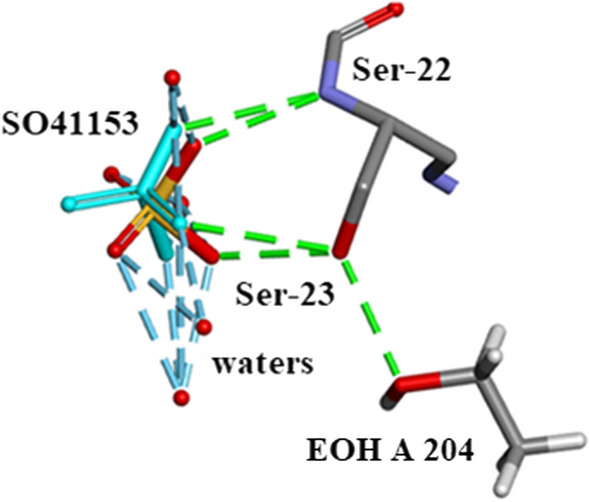
SO41153 Hydrogen Bonds to Ser-22, Ser-23, EOH A204 and several waters

**Fig. 26:**
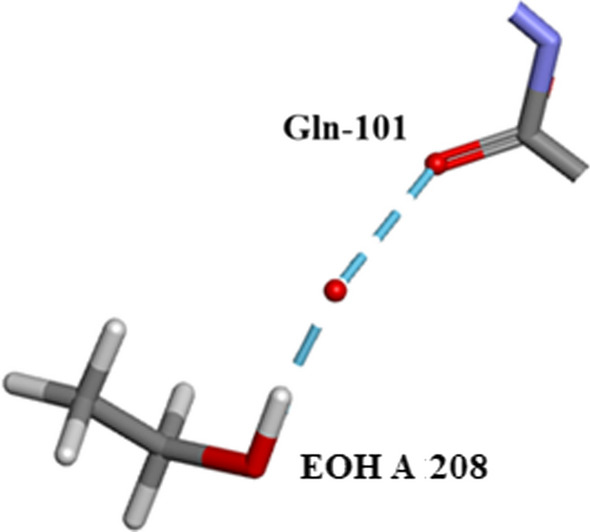
EOH A 208 links to Gln-101 via a water molecule

**Fig. 27:**
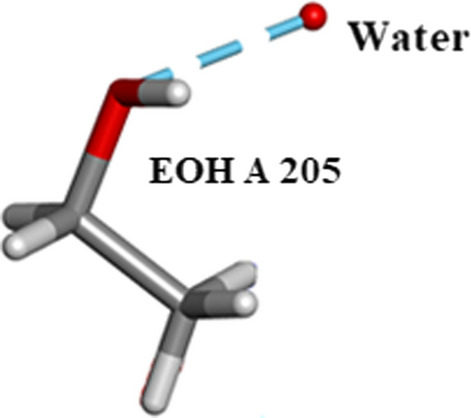
Showing EOH, A 205 hydrogen bonded to a water molecule

**Fig. 28:**
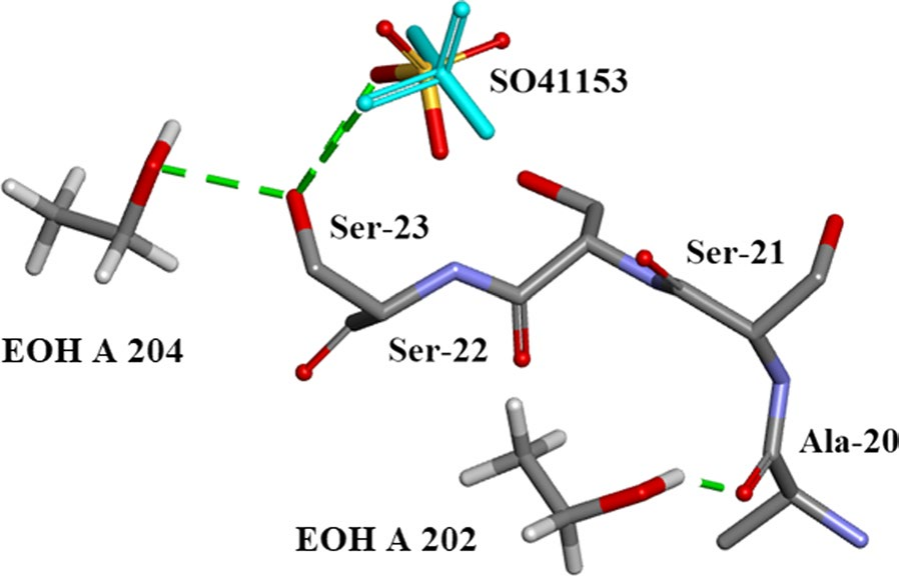
The sequence Ala-20 to Ser-23 and interactions with ethanol molecules EOH A 202 and EOH A 204

Five ${\text{SO}}_{4}^{2 - }$ moieties and eleven ethanol molecules were located and refined in the 0.85Å Orthorhombic RNase A structure designated SO41151 to1155 and EOH A201 to A211, respectively. H atoms were originally inserted geometrically into the EtOH molecules with Biovia [19]. Two of the ${\text{SO}}_{4}^{2 - }$ moieties SO41152 and SO41153 are disordered and refined into two conformations A and B. Most of these 12 solvates are located in or near to the intermolecular water bearing regions of the structure as can be seen in Fig. 21. Some examples are given in Figs. 22, 23, 24, 25, 26, 27 and 28. We have already seen: (i) the active site ${\text{SO}}_{4}^{2 - }$ A (SO41152A) in Fig. 5a and noted that it is linked via two water molecules to the protein main chain; (ii) the active site ${\text{SO}}_{4}^{2 - }$ (SO41152A and B) in Fig. 6a and noted links of site A with His-12, Gln-11 and a water molecule, and links of site B with Gln-11 and the same water molecule; and further details in Fig. 6. Figure 8c shows the hydrogen bonding of sulphate SO41154 with Alpha-Helix III and Fig. 9b, c show sulphate SO41152 H-bonding to Alpha-Helix I and Ethanol EOH1164 via a water molecule.

Figures 22, 23, 24, 25, 26, 27 and 28 are examples of the interactions between these solvates and the protein residues. It would appear that some degree of stabilization is achieved by these interactions.

## Study of the conserved waters

### ***ProBiS H***_***2***_***O***

**Fig. 29:**
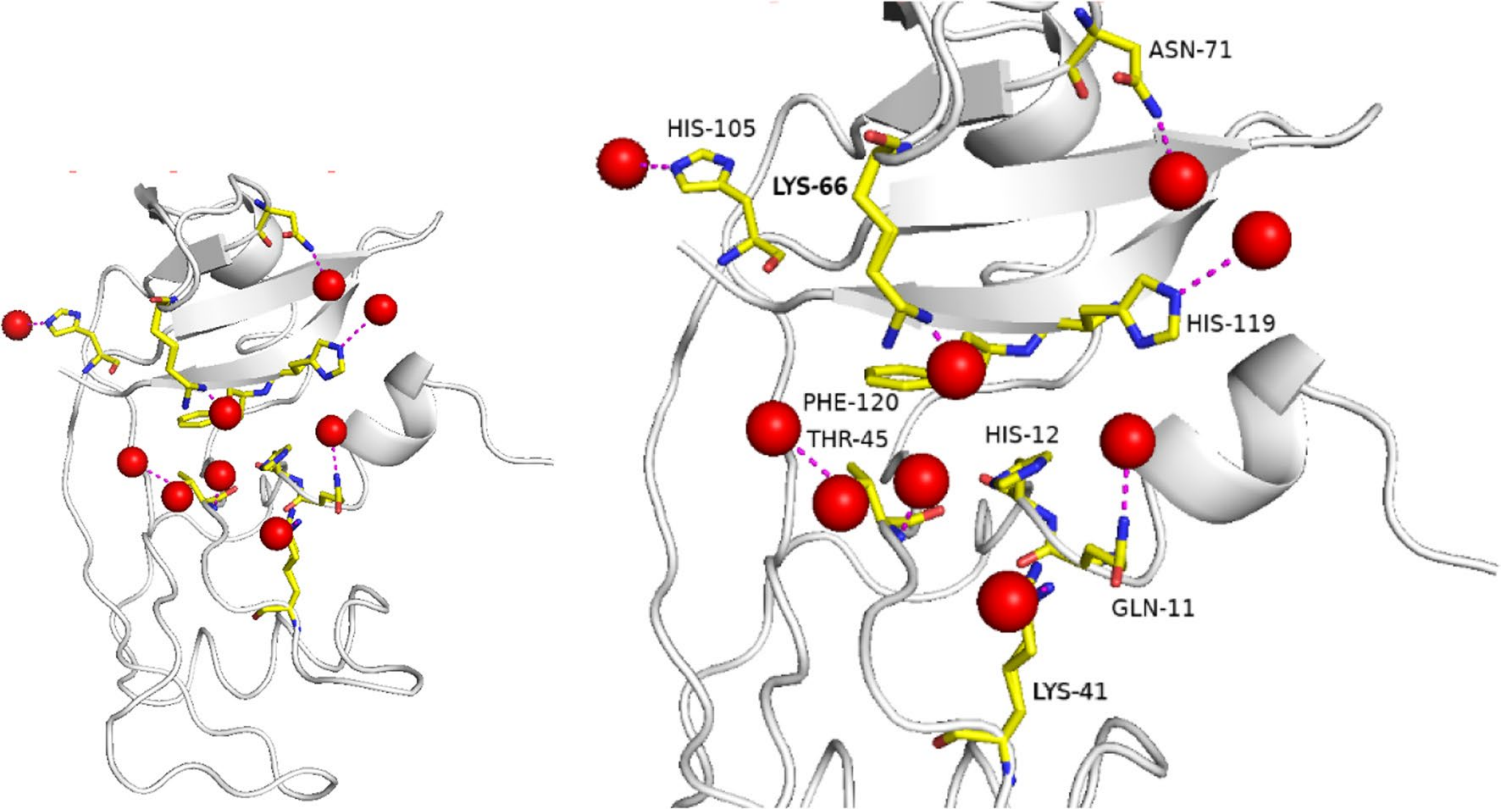
Many conserved waters were identified in the active site of Ribonuclease A (PDB ID: 7p4r). Left, whole structure, right, close-up of active site. Drawn with the PyMOL Molecular Graphics System, Version 2.0 Schrödinger, LLC

Conserved water sites were identified using ProBiS H_2_O cluster analysis [25], implemented as a plugin through the molecular visualisation system, PyMOL (v2.0). The ProBiS H_2_O algorithm superimposes similar protein structures available from the Protein Data Bank (PDB) with the query structure. The entire protein chains of four high resolution structures selected from the Protein DataBank, with PDB IDs: 6etl, [6] 1kf2, [7] 7rsa [26] and 3rn3, [1] were aligned with the Ribonuclease A structure (PDB ID: 7p4r) presented in this work (Table 5). The superimposition process allows water molecules to be transposed from the four structures to 7p4r, resulting in water clusters to be identified (Table 6). Figure 29 depicts the water clusters as large red spheres.

**Table 5:** Selected ribonuclease structures used in the ProBiS H_2_O algorithm

Coordinate set (PDB ID)	6etl	1kf2	7rsa	3rn3	7p4r
Precipitating agent	20 mM sodium citrate at pH 5.3 against 10 µl isopropanol 99.9% isopropanol	2-propanol, pH 5.2, Liquid diffusion, temperature 298 K	0.3 M sodium hydroxide, pH 5.3, 2-methyl-2-propanol 43% (v/v)	30–40% ethanol pH 5.2–5.7 stored in 60% aqueous EtOH	Distilled water. Absolute ethanol
pH	5.3	5.2	5.3	5.2–5.7	6.5
Resolution (Å)	0.85	1.10	1.26	1.45	0.85
Number of solvent molecules located	232	243	183	139	293
R factor	9.8	10.3	15.0	22.3	11.2

**Table 6:** Water clusters identified in Ribonuclease A (PDB ID: 7p4r)

Number of clusters	Number of H_2_O molecules per cluster	Conservation score
2	6	1.0
14	5	1.0
39	4	0.8
81	3	0.6
185	2	0.4

A sequence identity cut-off of 95% was used

### Conserved waters identified in Ribonuclease A at conserved sites

Conservation score is calculated as the number of water molecules in a certain position consistent in all similar structures and the query protein structure divided by the number of superimposed structures, multiplied by 100; the higher the conservation score indicates that the water cluster has a higher propensity to be in that location in the different protein structures [27].

Using ProBiS H_2_O, we identified several water clusters with conservation scores between 0.4 and 1.0 (Table 6). Several conserved waters were found near active site residues as described by Zegers et al. [28] (Fig. 29). Within the active site, seven residues make interactions with conserved waters (Table 7). Waters W86 and W91 make interactions with imidazole groups of His-105 and His-119, respectively. Both His-119 and His-12 are known to be involved in the catalytic mechanism for cyclization and cleavage of RNA [29]. Residue Thr-45 is shown to interact with three water clusters, including cluster W64; this cluster makes an interaction with the side chain OH group of Thr-45 and in turn makes an interaction with the side chain OH group of Ser-123. This residue is understood to have important hydrogen bond characteristics enabling the protein-substrate complex [28].

**Table 7:** Important residues forming bonds to water clusters in Ribonuclease A (PDB ID: 7p4r)

Residue	Water cluster	Conservation score	Bond distance W∙∙∙A (Å)
Glutamine 11	W107	1.0	3.0
Histidine 12	–	–	–
Lysine 41	W35	0.4	2.2
Threonine 45	W36	0.8	2.8
Threonine 45	W64	0.8	3.2
Threonine 45	W127	0.6	2.6
Lysine 66	W168	0.4	2.0
Asparagine 71	W90	0.6	3.0
Histidine 105	W86	0.6	2.9
Histidine 119	W91	0.4	3.5
Phenylalanine 120	–	–	–

### Conserved waters that interact with both the alpha helix and beta sheet

**Fig. 30:**
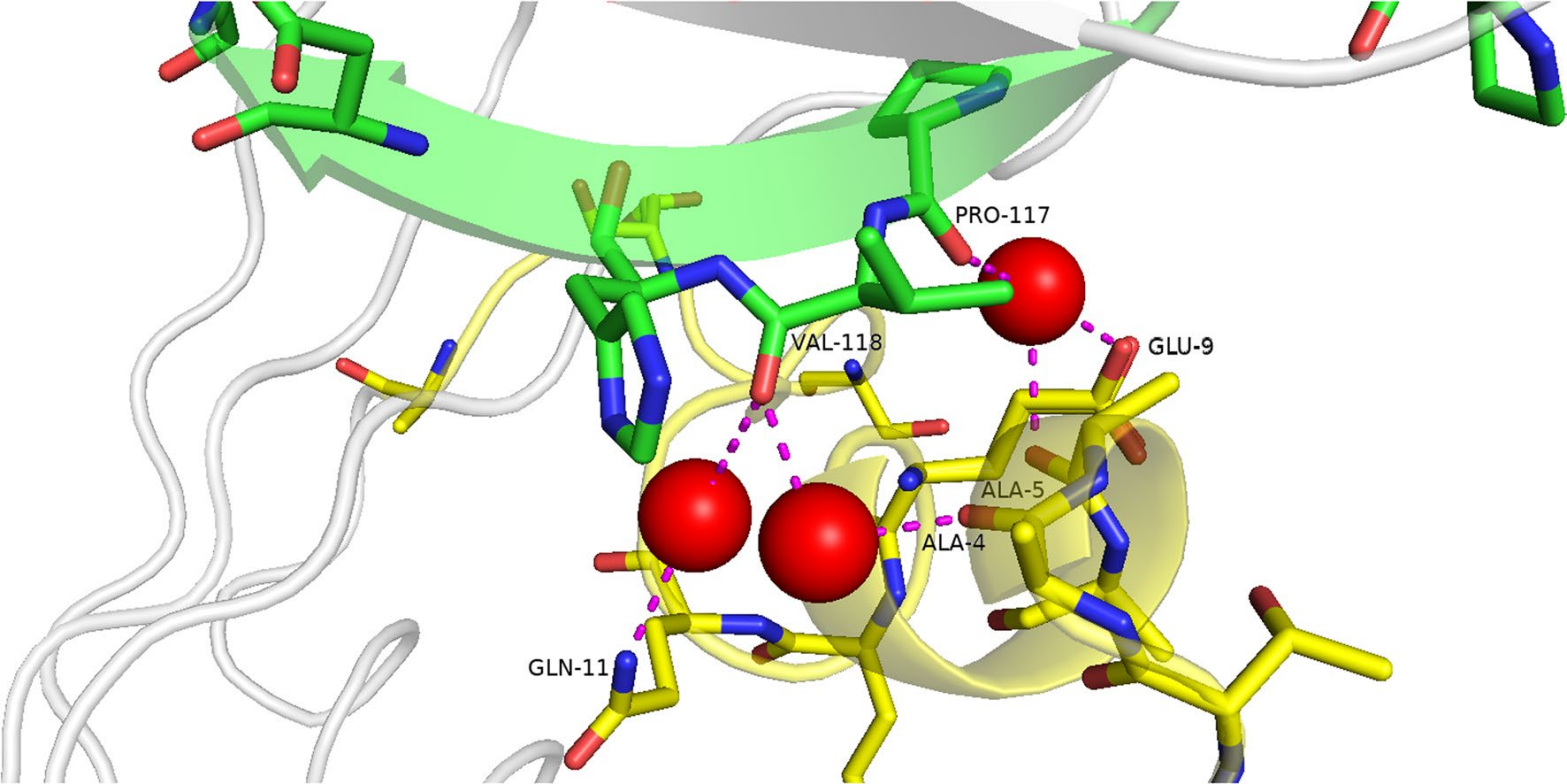
Ribonuclease A (PDB ID: 7p4r), residues from the alpha helix (yellow) and beta sheet (green). Drawn with the PyMOL Molecular Graphics System, Version 2.0 Schrödinger, LLC

Three water clusters interact with residues from both the alpha helix (1–22) and beta sheet (111–121), these are shown in Fig. 30. Cluster 107 has six H_2_O molecules with a conservation score of 1.0; this cluster is located between the alpha helix and beta sheet and makes interactions with residues Gln-11 (alpha helix) and Val-118 (beta sheet). Cluster 100 makes interactions with Glu-9 and Ala-5 (alpha helix) and Pro-117 (beta sheet); this cluster has five H_2_O molecules with a conservation score of 1.0. Cluster 101 has three H_2_O molecules with a conservation score of 0.6; this cluster makes interactions with Ala-4 (alpha helix) and Val-118 (beta sheet). These water clusters may have a very important role in stabilising the tertiary structure of the protein.

## Molecular dynamics studies on orthorhombic RNase A

### Introduction

As discussed previously, a molecular dynamics study has investigated His-119 which was modelled as two conformations in 3RN3 [5] and is observed to have a single clearly defined conformation in the present orthorhombic structure. MD was also used to investigate alternative conformations of Lys-31, Ser-32 and Lys-41. Lys-31 and Ser-32 have been modelled in the orthorhombic structure as each having 2 alternative conformations with occupancy A 62.6%, B = 37.4%, and A = 76%, B = 24%, respectively, whereas only single conformations occur in 3RN3. The Orthorhombic active site SO_4_ is also disordered.

#### Materials and methods

In order to prepare for the Molecular Dynamics (MD) simulation, the two files, 3RN3.pdb and the Orthorhombic Ribonuclease A described in this paper pdb including water molecules in the crystal structure were subjected to energy minimisation using the smart minimiser in MOE2019.09 [30]. Energy minimisation was performed using the AMBER14; EHT forcefield [31, 32], with only the original contents of the crystal structure contained in a periodic box, since the object of the MD simulation was to explain the disorder in the original unit cell of the high-resolution crystal structure, rather than a protein under normal solvated biological conditions. Following this, MD simulations were performed.

The simulation was run at 310 K for a production time of 300 ps, using an initial equilibration (heat) time of 100 ps. with data collected every 0.01 picoseconds, with a time step of 0.002 ps. using NVT dynamics under the Nosé Poincaré Andersen method [33, 34] with a Berendsen thermostat [35]. Data was collected with respect to torsion angles for the respective residues.

#### Results

**Fig. 31:**
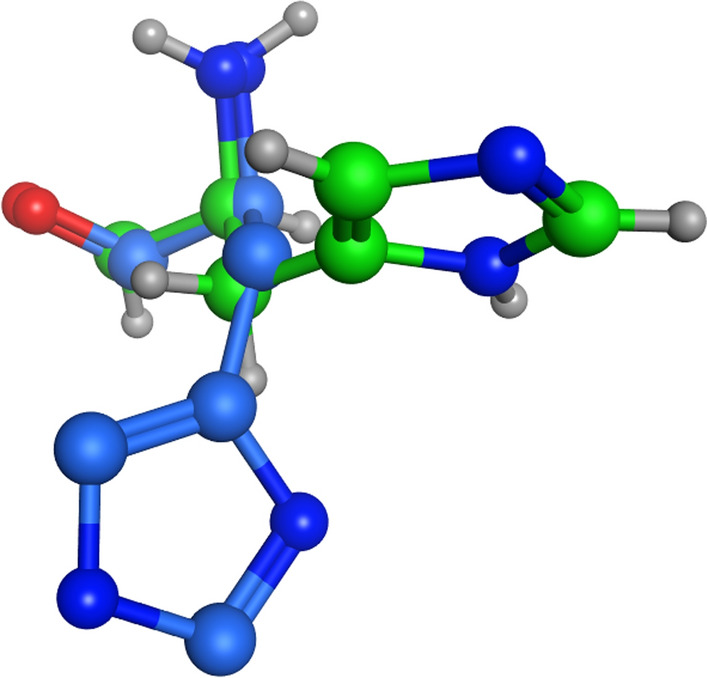
Starting conformations of His-119 in orthorhombic RNase A (green atoms) and 3RN3 (blue atoms)

The starting conformations for the His-119 residue are shown in Fig. 31. It can be clearly seen the orthorhombic RNase A starts the simulation in the minor ‘B’ position of 3RN3.

**Fig. 32:**
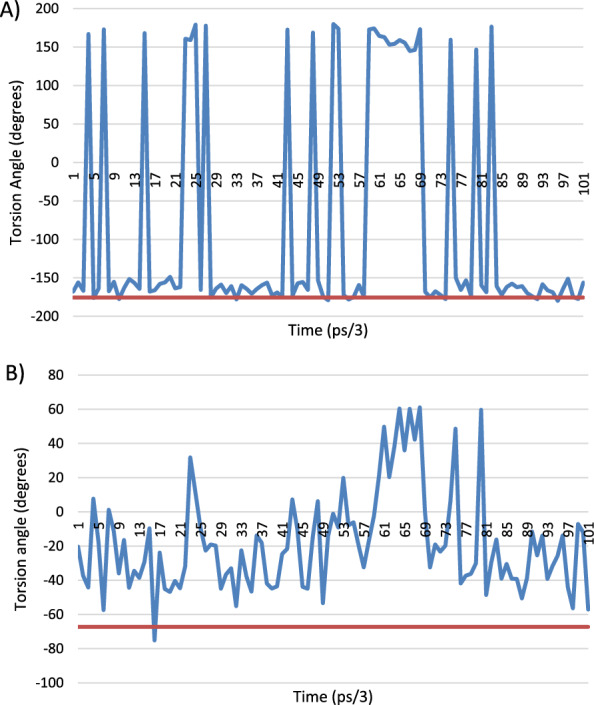
Torsion angles for His-119, the blue trace is the MD simulation of orthorhombic RNase A and the orange line is the crystallographically observed value. **A** C-CA-CB-CG torsion angle, **B** CA-CB-CG-ND1 torsion angle. The structure has just migrated to the second minimum and stays there because the movement of the protein hasn’t given it the energy to jump back. It doesn’t make any extra hydrogen bonds in this position and is just a consequence of thermal motion. If we extended the simulation to much longer time scales, we would expect to see many more of these events

The MD results for His-119 in the orthorhombic structure show the C-CA-CB-CG torsion angle moving between two values, i.e. − 175° to + 175° (Fig. 32A). The observed X-ray torsion angle for the Orthorhombic structure is at the − 175° minimum. The corresponding torsion angle for the main ‘A’ position for the 3RN3 structure is between these two positions at 42.8° whilst that of the ‘B’ position is at the second minimum at 168.94°. The other torsion angle (CA-CB-CG-ND1) shows a variety of minima in the MD (Fig. 32B), with the orthorhombic structure being situated in a shallow minimum at − 67.28°, whilst the 3RN3 conformations are also shallow minima at 79.17° and − 54.8° for the ‘A’ and ‘B’ positions, respectively. The ‘B’ position of 3RN3 corresponds most closely with the orthorhombic structure.

**Fig. 33:**
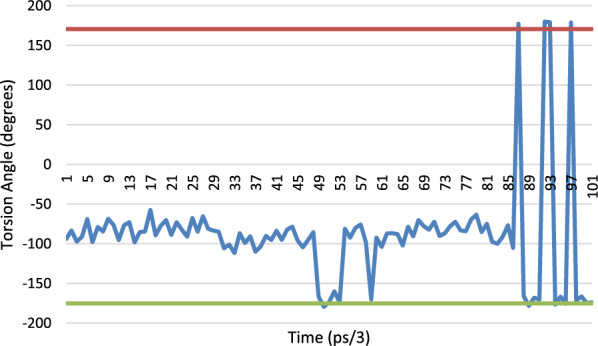
Torsion angle for CG-CD-CE-NZ for Lys-41, the blue trace is the MD simulation of orthorhombic RNase A and the red line is the crystallographically observed value for position A and the green line the crystallographically observed value for site B

There are two positions in the X-ray structure of Lys-41 in the orthorhombic structure, the MD results from the simulation of this structure show that for the C-CA-CB-CG torsion angle oscillates between 40° and 80°. The torsion angle of the A position corresponds nicely to this range (60.76°) whereas the B position is in an area not explored by the MD at 132.64°. The CA-CB-CG-CD torsion angle shows the same trend with the A position at the observed minimum in the MD simulation and the B position in-between. For the CB-CG-CD-CE torsion the same situation applies with the A position at a minimum (− 177°) and the B position between the 2 observed minima at − 43.5°. Only for the CG-CD-CE-NZ torsion angle do both the A and B positions occupy observed minima (Fig. 33).

The excel spreadsheet for all the Lys-41 torsions studied is given in Additional file 1: Table S5 together with the corresponding plots, Additional file 1: Figs S8, S9, S10 and S11.

**Fig. 34:**
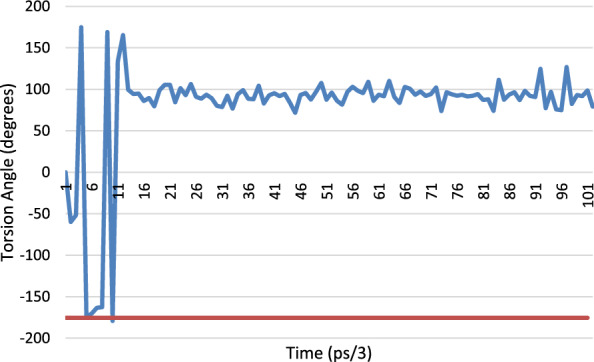
Torsion angle for CA-CB-CG-CD for Lys-31, the blue trace is the MD simulation of orthorhombic RNase A and the red line is the crystallographically observed value

Again, the crystallographically determined torsion angles for the 3RN3 structure correspond closely to the B position of the orthorhombic structure. In Lys-31 the orthorhombic structure is firmly in the MD minimum for C-CA-CB-CG and the 3RN3 one is not. Interestingly for the CA-CB-CG-CD torsion angle (Fig. 34), the orthorhombic structure sits in a minimum that is only explored by the MD at the start of the simulation whereupon it transits to another minimum which is the one occupied by the 3RN3 structure.

**Fig. 35:**
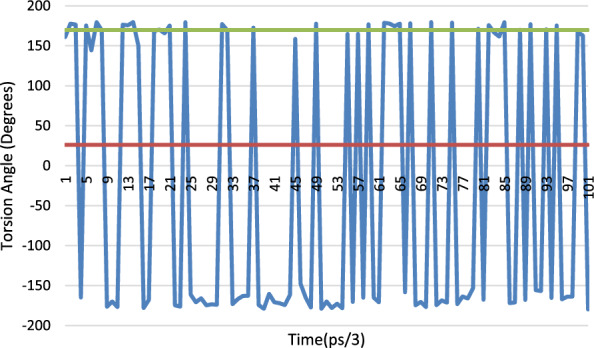
Torsion angle for C-CA-CB-OG for Ser-32, the blue trace is the MD simulation of orthorhombic RNase A and the red line is the crystallographically observed value for the A position and the green line the crystallographically determined value for the B site

Ser-32 also occupies 2 positions in the orthorhombic structure (Fig. 35), the torsion angle of the B position corresponding to the MD minimum and the A position being in between in this case. The 3RN3 position is also in between the MD minima (− 60.82°).

**Fig. 36:**
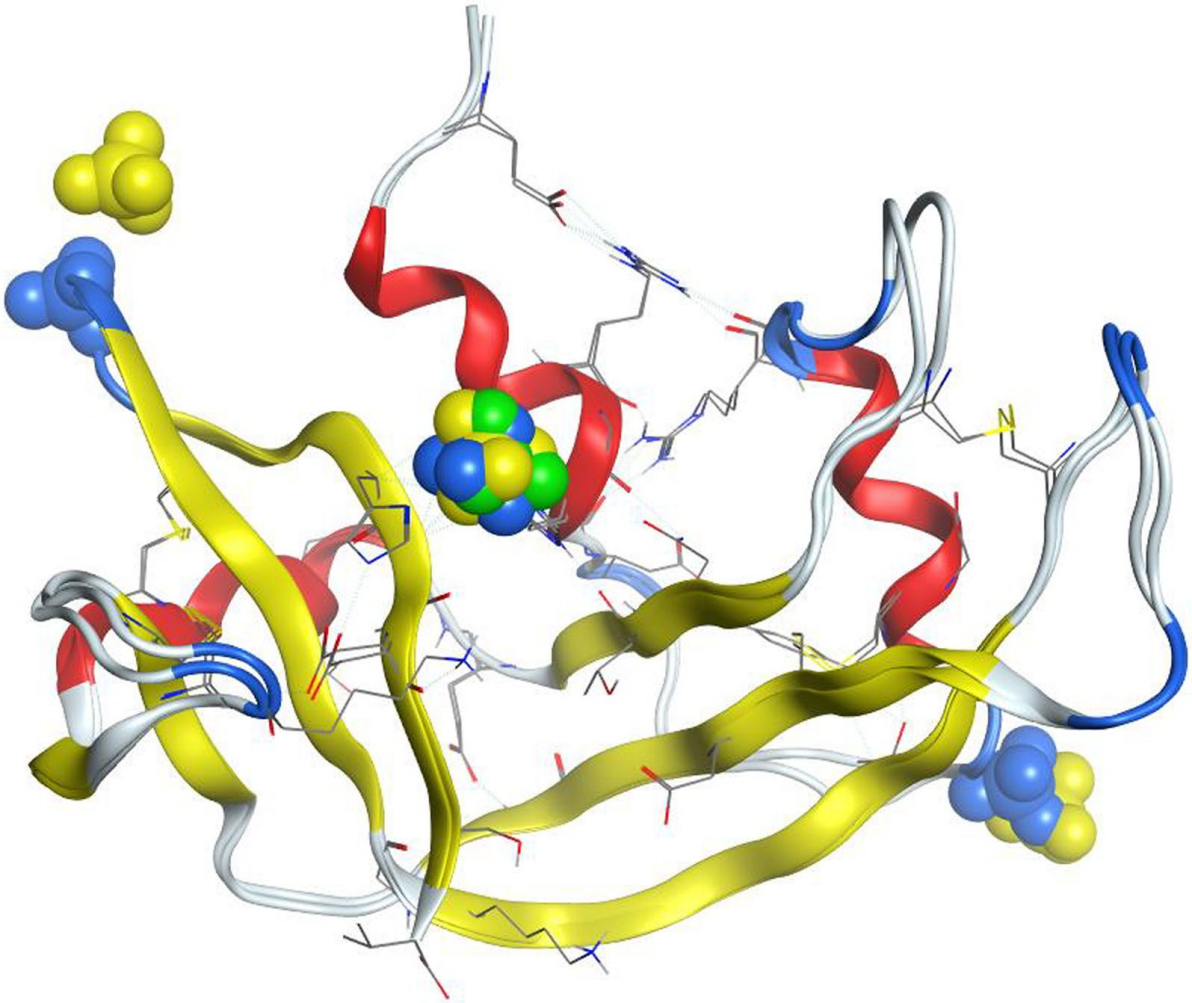
Overlay of the sulphate positions for orthorhombic RNase A (yellow atoms), 3RN3 (green atoms) and the position after 300 ps. of simulation of the MD simulation of the orthorhombic RNase A (blue atoms)

There are 3 positions for the sulphate anion in the orthorhombic structure and only one in the 3RN3 structure. An overlay of the sulphate positions is shown below (Fig. 36). The sulphate in the active site rotates in position during the MD simulation without moving from its centroid whereas the other two move about significantly during the simulation.

### Overall conclusions from the molecular dynamics study

If the alternative positions found for the residues in the X-ray structures result from thermal disorder, then they should also be accessible from the MD simulations. This does indeed seem to be the case for the B positions of both the orthorhombic and 3RN3 structures. However, this is not the case for the A positions, so they are more strongly influenced by static disorder, where they are in different positions in the mosaic that makes up the overall crystal.

## Analysis of subsidiary sites of orthorhombic RNase A

**Fig. 37:**
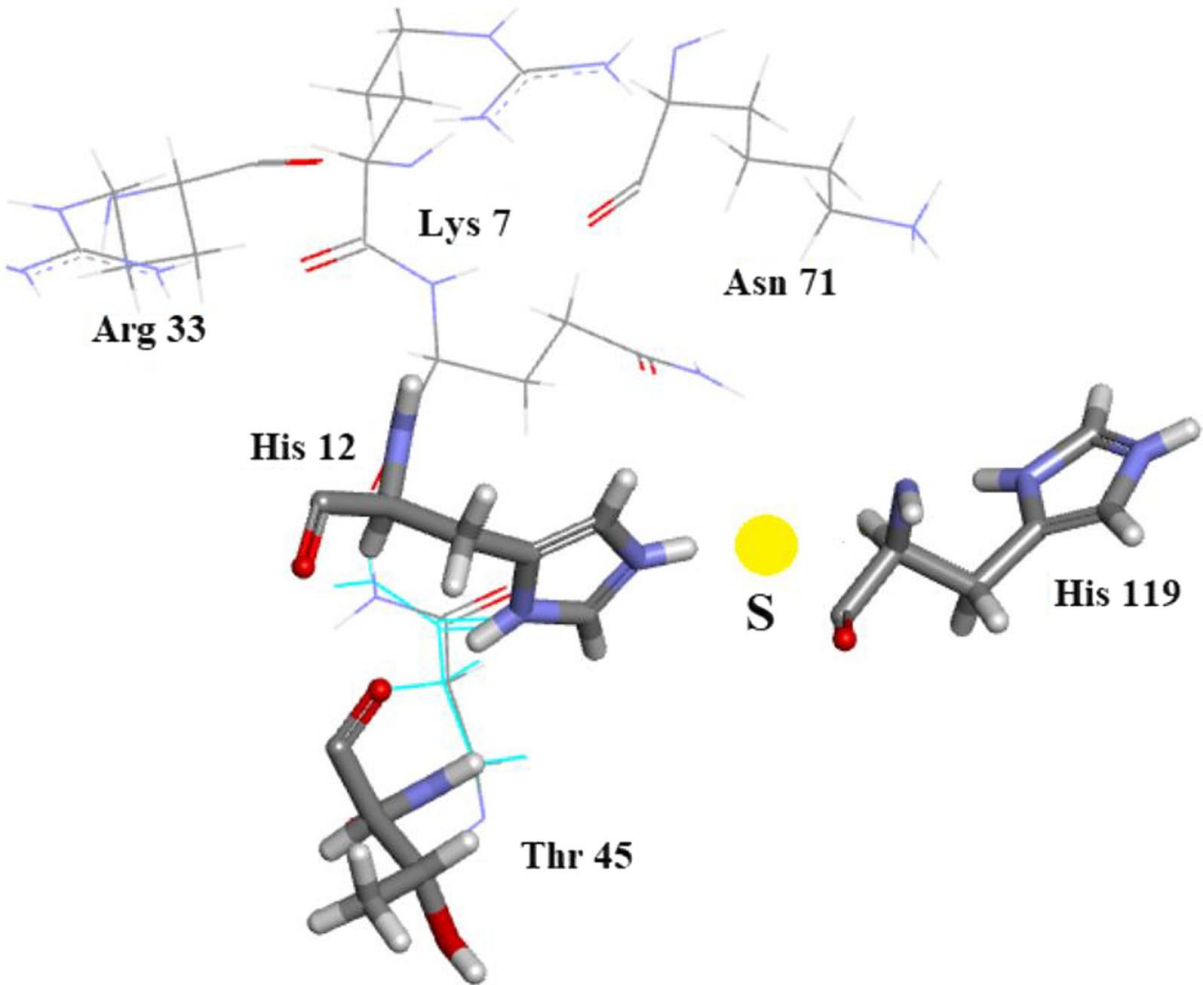
Subsidiary site

**Fig. 38:**
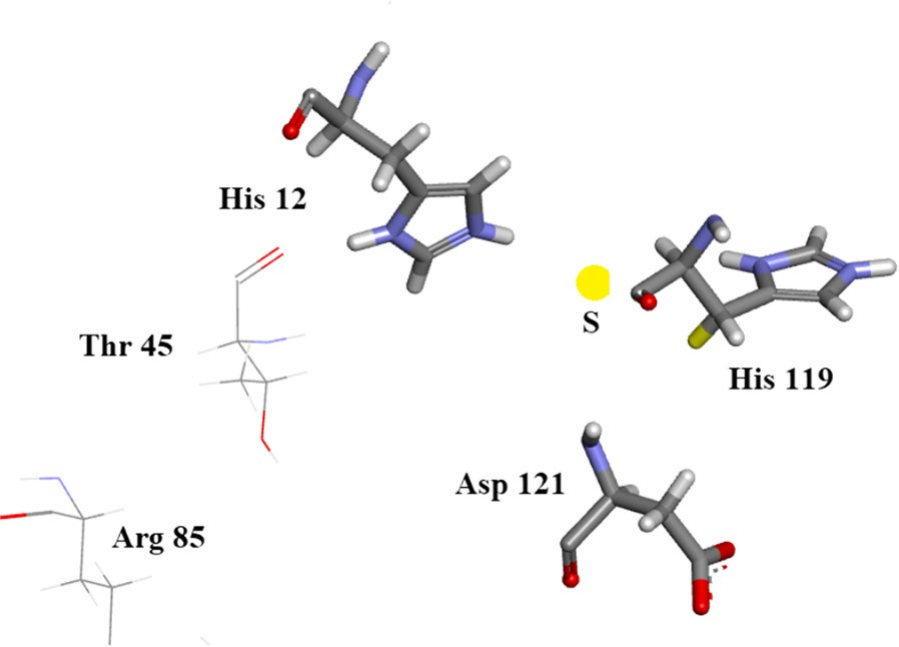
Subsidiary Site

The commonly accepted residues associated with the active site of Bovine RNase A are Lys 7, Gln 11, His 12, Lys 41, Asn 44 and His 119. Lys 41 is bound to two waters and His 119 to a single water molecule. This group of residues and water molecules has been studied with ultra-high precision in the present work. In a recent publication A New Remote Subsite in Ribonuclease A Fisher et al*.* [36]the active site is described in terms of biochemical studies recently carried out. In addition to the six residues listed above seven other residues seem to be proposed by these authors as having a bearing on the active site. They are: Arg 10, Asp 33, Thr 45, Lys 66, Asn 71, Arg 85, and Asp 121. A study of our model which includes as active site residues: Lys 7, Gln 11, His 12, Lys 41, Asn 44 and His 119, Figs. 37 and 38, where Lys 41 is bound to two waters and His 119 to a single water molecule leads to the following conclusions about the subsite residues of Fisher et al.: Arg 10 may be linked to active site residue Lys 7. Arg 33 has no links for interaction with active site residues. Thr 45 is possibly linked to active site residue His 12 and is of course bonded to active site residue Asn44. Lys 66 is blocked by Asp 121. Asn 71 is possibly within reach of the active site via water molecules. Arg 85 is too far removed to be associated with our active site. Asp 121 could link to His 119 but is badly oriented for H bond formation.

## Supplementary Information

**Additional file 1: Figure S1**: Orthorhombic RNase A. Ramachandran Plot for alpha helix I. With the exception of His-12 and Gln-11 the (φ, ψ) values are well placed in the alpha helix region. The distortion associated with these deviations is evident in Figure 8. **Figure S2**: Orthorhombic RNase A. Ramachandran Plot for alpha helix II. All the (φ, ψ) values are well placed in the alpha helix region. **Figure S3**: Ramachandran Plot for alpha helix III in Orthorhombic RNase A. The main chain conformation deviates slightly from the ideal at residue Ser-59. **Figure S4**: Ramachandran Plot for Beta Sheets in Orthorhombic RNase A. The main chain conformation is well within the accepted Beta Sheet region. **Figure S5**. The Side-Chain–Main–Chain H-bond for Asn-71 in Orthorhombic RNase A. **Table S1**: Numbers of Protein-Protein and Protein Solvent Hydrogen Bonds. **Table S2**: Numbers of Residues with m Protein-Protein and n Protein-Water Hydrogen Bonds Per Residue. **Table S3**. Comparison of observed electron densities between the Orthorhombic and Monoclinic structures using Coot [14]. **Figure S6**. The last three residues of Orthorhombic RNase A. (a) Electron density (Coot [14]) and (b) model (Biovia [17]). The electron density is complete but Val-124 is disordered. Compare this diagram with Figure S7 corresponding to 3RN3. **Figure S7**. The last three residues of 3RN3. (a) Electron density (Coot [14]) and (b) model (Biovia [17]). Part of the electron density is missing from the Val-124 side-chain. This is contrasted by the corresponding views of Orthorhombic RNase in **Figure S6**. The effect of a much higher resolution, 0.85Å compared to 1.5Å, is easy to see. The density for the orthorhombic structure is also complete for this excerpt of the structure. In (b) the coloured wavy lines indicate the course of the main chain. **Table S4**. Comparison of PISA interfaces between Ribonuclease 3rn3 and 7p4r. Table S5 MD For Lys-41. **Figure S8**. Torsions C-CA-CB-CG for Lysine 41 generated from 300ps of molecular dynamics. Figure S9. Torsions CA-CB-CG-CD for Lysine 41 generated from 300ps of molecular dynamics. **Figure S10**. Torsions CB-CG-CD-CE for Lysine 41 generated from 300ps of molecular dynamics. **Figure S11**. Torsions CG-CD-CE-NZ for Lysine 41 generated from 300ps of molecular dynamics.

## Data Availability

The datasets generated during and/or analysed during the current study are available in the Protein Databank repository, [https://www.rcsb.org/structure/unreleased/7P4R]. Entry on hold until publication.
