# Supplementary material for: Ultra-high resolution X-ray structure of orthorhombic bovine pancreatic Ribonuclease A at 100K
Source: BMC Chem. 2023 Jul 27;17(1):91. doi: 10.1186/s13065-023-00959-6 (PMC10375658; doi:10.1186/s13065-023-00959-6)
Supplement: Supplementary file 1 — Additional file 1: Figure S1: Orthorhombic RNase A. Ramachandran Plot for alpha helix I. With the exception of His-12 and Gln-11 the (φ, ψ) values are well placed in the alpha helix region. The distortion associated with these deviations is evident in Figure 8. Figure S2: Orthorhombic RNase A. Ramachandran Plot for alpha helix II. All the (φ, ψ) values are well placed in the alpha helix region. Figure S3: Ramachandran Plot for alpha helix III in Orthorhombic RNase A. The main chain conformation deviates slightly from the ideal at residue Ser-59. Figure S4: Ramachandran Plot for Beta Sheets in Orthorhombic RNase A. The main chain conformation is well within the accepted Beta Sheet region. Figure S5. The Side-Chain–Main–Chain H-bond for Asn-71 in Orthorhombic RNase A. Table S1: Numbers of Protein-Protein and Protein Solvent Hydrogen Bonds. Table S2: Numbers of Residues with m Protein-Protein and n Protein-Water Hydrogen Bonds Per Residue. Table S3. Comparison of observed electron densities between the Orthorhombic and Monoclinic structures using Coot [14]. Figure S6. The last three residues of Orthorhombic RNase A. (a) Electron density (Coot [14]) and (b) model (Biovia [17]). The electron density is complete but Val-124 is disordered. Compare this diagram with Figure S7 corresponding to 3RN3. Figure S7. The last three residues of 3RN3. (a) Electron density (Coot [14]) and (b) model (Biovia [17]). Part of the electron density is missing from the Val-124 side-chain. This is contrasted by the corresponding views of Orthorhombic RNase in Figure S6. The effect of a much higher resolution, 0.85Å compared to 1.5Å, is easy to see. The density for the orthorhombic structure is also complete for this excerpt of the structure. In (b) the coloured wavy lines indicate the course of the main chain. Table S4. Comparison of PISA interfaces between Ribonuclease 3rn3 and 7p4r. Table S5 MD For Lys-41. Figure S8. Torsions C-CA-CB-CG for Lysine 41 generated from 300ps of molecular dynamic [file 13065_2023_959_MOESM1_ESM.docx]

**Additional file 1**

**Ultra-High Resolution X-ray Structure of Orthorhombic Bovine Pancreatic Ribonuclease A at 100K**

**David. R. Lisgarten^a^, Rex A. Palmer^b*^, Jon B. Cooper^c^ , Claire E. Naylor^d^, Rosemary. C. Talbert^a^, Brendan J. Howlin^e^, John N. Lisgarten^f^ , Janez Konc^g^, Shabir Najmudin^h^ and Carina M. C. Lobley^i^**

(a)Biomolecular Research Group, School of Psychology and Life Sciences, Canterbury Christ Church University, North Holmes Road, Canterbury, Kent CT1 1QU, UK [david.lisgarten@canterbury.ac.uk](mailto:david.lisgarten@canterbury.ac.uk) +44(0)1227783903, [rosemary.talbert@canterbury.ac.uk](mailto:rosemary.talbert@canterbury.ac.uk) +44(0)1227783924

(b) Department of Crystallography, Biochemical Sciences, Birkbeck College, Malet St, London WC1E7HX, UK [rex.palmer@btinternet.com](mailto:rex.palmer@btinternet.com) +44( 0)2084491049

(c) Division of Medicine, UCL, Gower Street, London, WC1E 6BT and Department of Biological Sciences, Birkbeck, University of London, Malet Street, Bloomsbury, London, WC1E 7HX. jon.cooper@ucl.ac.uk

(d)

[Kisaco Research, 41a Maltby Street, London, SE1 3PA. censtares@googlemail.com](mailto:Kisaco Research, 41a Maltby Street, London, SE1 3PA. censtares@googlemail.com)

(e) Chemical Sciences Division, Faculty of Health and Medical Sciences, University of Surrey, Guildford, Surrey GU2 7HX, UK b.howlin@surrey.ac.uk+44(0)1483300800.

(f) School of Science, University of Greenwich (Medway Campus), Chatham Maritime,

Kent ME4 4TB, UK [J.Lisgarten@greenwich.ac.uk](mailto:J.Lisgarten@greenwich.ac.uk)

(g) National Institute of Chemistry, Hajdrihova 19 SI-1000, Ljubljana, Slovenia. konc@cmm.ki.si

(h) Randall Centre for Cell and Molecular Biophysics, 3rd floor New Hunt's House, Faculty of Life Sciences and Medicine, King's College, London SE1 1UL, UK [shabir.najmudin@kcl.ac.uk](mailto:shabir.najmudin@kcl.ac.uk).

(i) European Spallation Source ERIC Lund, Kalmar County, Sweden carina.lobley@diamond.ac.uk


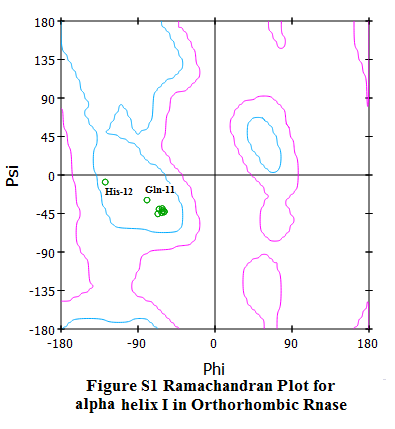


**Figure S1: Orthorhombic RNase A. Ramachandran Plot for alpha helix I. With the exception of His-12 and Gln-11 the (φ, ψ) values are well placed in the alpha helix region. The distortion associated with these deviations is evident in Figure 8.**

**
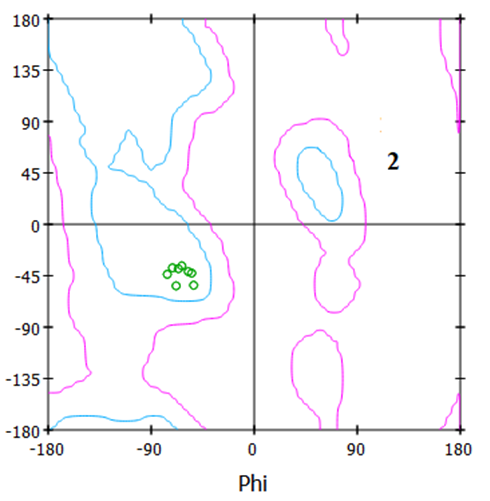
**

**Figure S2: Orthorhombic RNase A. Ramachandran Plot for alpha helix II. All the (φ, ψ) values are well placed in the alpha helix region.**


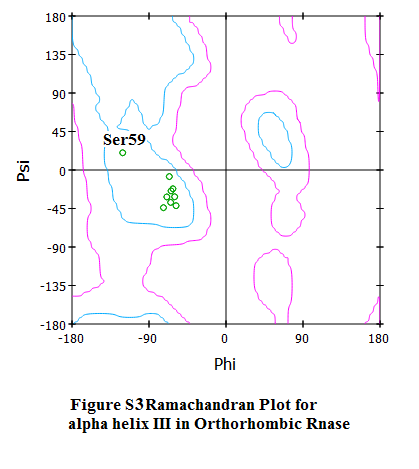


**Figure S3: Ramachandran Plot for alpha helix III in Orthorhombic RNase A. The main** **chain conformation deviates slightly from the ideal at residue Ser-59.**

**
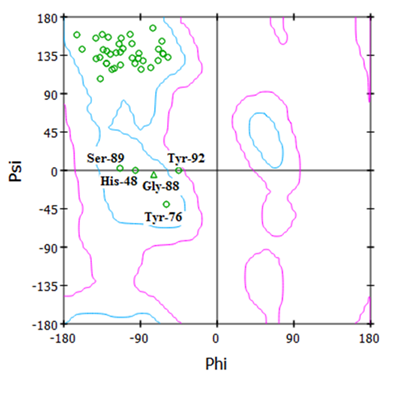
**

**Figure S4: Ramachandran Plot for Beta Sheets in Orthorhombic RNase A. The main chain conformation is well within the accepted Beta Sheet region.**

**Figure S5. The Side-Chain – Main – Chain H-bond for Asn-71 in Orthorhombic RNase A.**

**Table S1: Numbers of Protein-Protein and Protein Solvent Hydrogen Bonds**

Residue Protein-Protein Protein-Water Solvent

K1 Lys-1 2 [Glu-2 Thr-3] 4

E2 Glu-2 2 [Lys-1 Arg-10] 3

T3 Thr-3 4 [Lys-1Ala-5Ala-6 Lys-7] 0

A4 Ala-4 1 [Phe-8] 1

A5 Ala-5 4 [Thr-3, Phe-8] 2

[Glu-9, Pro-117]

A6 Ala-6 3 [Thr-3, Glu-9, Arg-10] 1

K7 Lys-7 3 [Thr-3, Arg-10, Gln-11] 0

F8 Phe-8 3 [Ala-4, Ala-5, His-12] 0

E9 Glu-9 4 [Ala-5, His-12] 5

[Arg-33, Ala-6]

R10 Arg-10 5 [Lys-7, Glu-2] 0

Fig 9 [Arg-33, Asn-34, Ala-6] 0

Q11 Gln-11 3 [Lys-7, Lys-41, Asn-44] 2 SO41152 H2O2174

H12 His-12 5 [Phe-8, Glu-9, Thr-45] 0

[Phe-46, Val-47]

X13 Met-13 2 [Glu-9, Arg-33] 0

D14 Asp-14 5 [Arg-33, Val-47, Thr-17] 0

[Tyr-25, His-48]

S15 Ser-15 1 [Glu-49] 0 SOE1168

S16 Ser-16 0 2 HOH2040 HOH2028

T17 Thr-17 2 [Asp-14], [His-48] 0

S18 Ser-18 1 [Ser-80] 2

A19 Ala-19 1 [Gln-101] 0

A20 Ala-20 1 [Gln-101] 1 ETOH1162/11163 H202163

S21 Ser-21 0* 0

S22 Ser-22 2 [Asn-24, Tyr-25] 0 SO41153

S23 Ser-23 0 1 SO41153 EOH1165

N24 Asn-24 3 [Ser-22, Asn-27, Gln-28] 2 ETOH1165

Y25 Tyr-25 4 [Ser-22, Asp-14, Gln-28] 0

[Met-29]

C26 Cys-26 2 [Thr-99, Met-30] 0

N27 Asn-27 3 [Asn-24, Tyr-97, Lys-31] 2

Q28 Gln-28 4 [Asn-24, Tyr-25, Lys-31] 2

[Ser-32]

M29 Met-29 2 [Tyr-25, Arg-33] 1

M30 Met-30 2 [Cys-26, Leu-35] 0

K31 Lys-31 3 [Asn-27, Gln-28, Asn-34] 1

S32 Ser-32 1 [Gln-28] 1

R33 Arg-33 4 [Asp-14, Arg-10, Met-29] 0 [Glu-9]

N34 Asn-34 3 [Arg-10, Lys-31, Lys-37] 1

L35 Leu-35 2 [Met-30, Lys-41] 1

T36 Thr-36 1 [Arg-39] 1

K37 Lys-37 2 [Arg-39, Asn-34] 1

D38 Asp-38 1 [Tyr-92] 2

R39 Arg-39 1 [Lys-37, Thr-36] 1

Residue Protein-Protein Protein-Water Solvent

C40 Cys-40 1 [Pro-42] 1

K41 Lys-41 4 [Leu-35, Gln-11] 2 SO41152

[Tyr-97, Val-43]

P42 Pro-42 3 [Glu-86, Cys-40, Arg-85] 0

V43 Val-43 2 [Lys-41, Cys-84] 0

N44 Asn-44 2 [Cys-84, Gln-11] 2

T45 Thr-45 3 [His-12, Asp-83, Thr-82] 2

F46 Phe-46 3 [Thr-82, His-12, Ile-81] 0

V47 Val-47 4 [Asp-14, His-12] 0

[Glu-49, Ser-80]

H48 His-48 3 [Ser-80, Asp-14, Thr-17] 0

E49 Glu-49 4 [Ser-15, Val-47] 0

[Ser-80, Met-79]

S50 Ser-50 2 [Asp-53, Val-54] 0 SOE1168

L51 Leu-51 2 [Val-54, Gln-55] 0 SOE1168

A52 Ala-52 2 [Ala-56, Gln-55] 1 SOE1168

D53 Asp-53 1 [Ser-50] 1

V54 Val-54 3 [Ser-50, Leu-51, Val-57] 0

Q55 Gln-55 5 [Leu-51, Cys-58, Ala-52] 2

[Val-116, Prp-117]

A56 Ala-56 3 [Ala-52, Ser-59, Gln-60] 0

V57 Val-57 2 [Val-54, Gln-60] 0

C58 Cys-58 1 [Gln-55] 0

S59 Ser-59 1 [Ala-56] 3

Q60 Gln-60 5 [Val-57, Ala-56, Asn-62] 2

[Tyr-76, Ser-75]

K61 Lys-61 2 [Gln-74, Tyr-73] 0

N62 Asn-62 3 [Gln-60, Thr-70, Cys-72] 0 EOH 1161 [H-bond needs work]

V63 Val-63 1 [Cys-72] 0

A64 Ala-64 1 [Gln-69] 1

C65 Cys-65 3 [Gln-69, Gly-68, Asp-121] 1

K66 Lys-66 1 [Asp-121] 1

N67 Asn-67 1 [Gln-69] 2

G68 Gly-68 1 [Cys-65] 1

Q69 Gln-69 3 [Cys-65, Asn-67, Ala-64] 2

T70 Thr-70 1 [Asn-62] 0

N71 Asn-71 3 [Asn-71*, Cys-110] 1

[Ala-109]

C72 Cys-72 3 [Asn-62, Val-63] 0

Y73 Tyr-73 3 [Val-108, Lys-61, Ile-107] 0

Q74 Gln-74 3 [Lys-61, Tyr-76, Ile-106] 2

S75 Ser-75 3 [Ile-106, His-105, Gln60] 1

Y76 Tyr-76 3 [His-105, Gln-60, Gln-74] 5

S77 Ser-77 1 [Thr-78] 2

Residue Protein-Protein Protein-Water Solvent

T78 Thr-78 2 [Asn-103, Ser-77] 4

M79 Met-79 3 [Lys-104, Glu-49, Asn-103]0

S80 Ser-80 6 [His-48, Glu-49, Ser-18] 0

[Gln-101, Val-47, Asn-103]

I81 Ile-81 3 [Ala-102, Phe-46, Gln-101] 0

T82 Thr-82 4 [Phe-46, Gln-101, Thr-45] 0

[Thr-100]

D83 Asp-83 5 [Arg-85, Thr-45, Thr-100] 6

[Cys-84, Thr-99]

C84 Cys-84 4 [Asn-44, Asp-83] 0

[Val-43, Lys-98]

R85 Arg-85 4 [Asp-83, Lys-98] 1

[Pro-42, Tyr-97]

E86 Glu-86 3 [Pro-42, Ala-96, Ser-90] 3

T87 Thr-87 2 [Ala-96, Ser-90] 1

G88 Gly-88 0* 1

S89 Ser-89 0* 3 SOE1169

S90 Ser90 3 [Thr-87, Ala-96, Glu-86] 0

K91 Lys-91 2 [Tyr-92, Asn-44] 2

Y92 Tyr-92 2 [Asp-38, Lys-91] 1

P93 Pro-93 1 [Cys-95] 1

N94 Asn-94 1 [Lys-91] 0

C95 Cys-95 1 [Pr0-93] 2

A96 Ala-96 3 [Ser-90, Thr-87, Glu-86] 0

Y97 Tyr-97 3 [Asn-27, Lys-41, Arg-85] 4

K98 Lys-98 2 [Arg-85, Cys-84] 1

T99 Thr-99 2 [Cys-26, Asp-83] 4

T100 Thr-100 2 [Asp-83, Thr-82] 0

Q101 Gln-101 5 [Ala-20, Thr-82, Ser-80] 3

[Ala-19, Ile-81]

A102 Ala-102 1 [Ile-81] 0

N103 Asn-103 3 [Thr-78, Ser-80, Met-79] 2

K104 Lys-104 2 [Met-79, Val-124] 1

H105 His-105 4 [Ser-75, Val-124] 0

[Tyr-76, Ser-123]

I106 Ile-106 3 [Ser-75, Gln-74, Ala-122] 0

I107 Ile-107 3 [Tyr-73, Ala-122, Asp-121] 0

V108 Val-108 3 [Tyr-73, Cys-72, His-119] 0

A109 Ala-109 4 [His-119, Val-118] 0

[Asn-71, Pro-117]

C110 Cys-110 2 [Asn-71, Val-116] 0

E111 Glu-111 2 [Val-116, Tyr-115] 3

G112 Gly-112 1 [Tyr-115] 0 SO41151

Residue Protein-Protein Protein-Water Solvent

N113 Asn-113 1 [Pro-114] 1 SO41151

P114 Pro-114 2 [Val-116, Asn-113] 1

Y115 Tyr-115 2 [Glu-111, Gly-112] 2

V116 Val-116 4 [Glu-111, Pro-114] 0

[Cys-110, Gln-55]

P117 Pro-117 3 [Ala-5, Ala-109, Gln55] 3

V118 Val-118 1 [Ala-109] 1

H119 His-119 2 [Ala-109, Val-108] 1 SO41152

F120 Phe-120 0 3 SO41152

D121 Asp-121 3 [Lys-66, Ile-107, Cys-65] 3

A122 Ala-122 2 [Ile-107, Ile-106] 1

S123 Ser-123 1 [His-105] 3

V125 Val-124 1 [His-105, Lys-104] 1

Notes: The residues in Orthorhombic RNase A are listed in order from 1 to 124.

Explanation: For example, starting at the top of the list Lys-1 forms 2 H-bonds with Glu-2 and Thr-3 respectively and 4 H-bonds with water molecules. Ser-21 forms no H-bonds with other residues or with water molecules. *Unusually Asn-71 forms probably the only inter-chain H-bond (see Figure 12). No other H-bonds were detected for Asn-71.

**Table S2: Numbers of Residues with m Protein-Protein and n Protein-Water Hydrogen Bonds Per Residue:**

Protein-Protein Protein-Water Number of Residues Special Remarks

m n

0 0 1 Ser-21 1 0 6

2 0 12

3 0 13

4 0 10

5 0 2

6 0 1 Ser-80

0 1 2

1 1 10

2 1 4

3 1 2

4 1 1 Arg-85

0 2 2

1 2 5

2 2 3

3 2 7

0 3 1 Phe-120

1 3 2

2 3 2

3 3 3

4 3 0

5 3 1 Gln-101

0 4 0

1 4 0

2 4 2

0 5 0

1 5 0

2 5 0

3 5 1 Tyr-76

4 5 1 Glu-9

5 6 1 Asp-83

Explanation: This table summarises the number of residues with m Protein-Protein and n Protein-Water H-bonds. Thus Ser-21 has neither and there are 10 residues with 1 Protein-Protein and 1 Protein-Water H-bond.

**Table S3. Comparison of observed electron densities between the Orthorhombic and Monoclinic structures using Coot [14].**

3

1. Using Coot [14] a complete survey of the electron density of individual amino acid residues in bovine pancreatic ribonuclease X-ray structures has been carried out: (a) the present 0.85Å resolution orthorhombic structure at 100K (18 problem areas) and (b) the 1.45Å resolution monoclinic structure at RT of Howlin 1989 [ ] 24 problem areas. Those residues where problems have been detected are highlighted.
2. Regions where the same or similar defects have been observed are: Lys-1, Gln-28, Lys-31, Lys-41, Lys-66, Asp-83, Lys-91. This list includes 5 Lysines.
3. Lys-1 and Thr-3 are close to the N-terminus.
4. His-119 is close to the C-terminus and Val-124 is at the C-terminus.
5. In the active site for Orthorhombic RNase only Lys-41 is bifurcated.

For 3RN3[1] Lys-7 has missing density; Lys-41 has poor density; His-119 is bifurcated.

**(a) 0.85Å 100K (orthorhombic) (b) 1.45Å (monoclinic)**

K1 Weak and missing after Cβ No density after Cδ

E2 Clear and complete density Clear and complete density

T3 Clear and complete density Clear except for CH_3_

A4 Clear and complete density Clear and complete density

A5 Clear and complete density Clear and complete density

A6 Clear and complete density Clear and complete density

K7 Clear and complete density Clear but missing end density

F8 Clear and complete density Clear and complete density

E9 Clear and complete density Clear and complete density

Except for one oxygen missing

R10 Clear and complete density Clear and complete density

Q11 Clear and complete density Clear and complete density

H12 Clear and complete density Clear and complete density

M13 Clear and complete density Clear and complete density

D14 Clear and complete density Clear and complete density

S15 Modelled with double end OH Clear and complete density

S16 Clear and complete density Missing OH at end

T17 Clear and complete density Clear and complete density

S18 OH disordered Clear and complete density

Density otherwise good

A19 Clear and complete density Clear and complete density

A20 Clear and complete density Clear and complete density

S21 Clear and complete density Clear and complete density

S22 Clear and complete density Clear and complete density

S23 Clear and complete density Clear and complete density

N24 Clear and complete density Problems and missing density

at end of chain

Y25 Clear and complete density Clear and complete density

C26 Slight disorder at end of chain Clear and complete density N27 Clear and complete density Clear and complete density

Q28 Clear but weak Missing density at end of chain M29 Clear and complete density Clear and complete density M30 Clear and complete density Clear and complete density

K31 Side chain bifurcated but Clear but density missing after Cδ

density good

S32 Disorder at end of side chain Clear and complete density

R33 Clear and complete density Clear and complete density

N34 Clear and complete density Clean but middle density missing

L35 Side chain is bifurcated. Clear and complete density

Good density.

T36 Clear and complete density Clear and complete density

K37 Clear and complete density Poor density badly modelled D38 Clear and complete density Clear and complete density

R39 Clear and complete density Poor density after Cβ

C40 Clear and complete density Clear and complete density

K41 Clear but weak density End parts missing and badly modelled

P42 Clear and complete density Clear and complete density

V43 Clear and complete density Good but one CH_3_ missing

N44 Clear and complete density Clear and complete density

T45 Clear and complete density Clear and complete density

F46 Clear and complete density Clear and complete density

V47 Clear and complete density Clear and complete density

H48 Clear and complete density Clear and complete density

E49 Clear and complete density Clear and complete density

S50 Clear and complete density Clear and complete density

L51 Good density but centrally Clear and complete density

bifurcated

A52 Clear and complete density Clear and complete density

D53 Clear and complete density Clear and complete density

V54 Clear and complete density Clear and complete density

Q55 Totally bifurcated Clear and complete density

Density good

A56 Clear and complete density Clear and complete density

V57 Clear and complete density Clear and complete density

C58 Clear and complete density Clear and complete density

S59 Clear and complete density Clear and complete density

Q60 Clear and complete density Clear and complete density

K61 Clear and complete density End density missing

N62 Clear and complete density Clear and complete density

V63 Clear and complete density Clear and complete density

A64 Clear and complete density Clear and complete density

C65 Clear and complete density Clear and complete density

K66 Double conformation at end Very poor density mostly missing

N67 Clear and complete density Clear and complete density

G68 Clear and complete density Clear and complete density

Q69 Clear and complete density Clear and complete density

T70 Clear and complete density Clear and complete density

N71 Clear and complete density Clear and complete density

C72 Clear and complete density Clear and complete density

Y73 Clear and complete density Clear and complete density

Q74 Clear and complete density Clear and complete density

S75  Clear and complete density Clear and complete density

Y76 Density good Clear and complete density but double conformation

S77 Clear and complete density Poor density and missing at end

T78 Clear and complete density Clear and complete density

M79 Clear and complete density Clear and complete density

S80 Clear and complete density Clear and complete density

I81 Clear and complete density Clear and complete density

T82 Clear and complete density Clear and complete density

D83 Goo density one O missing at end of chain but double conformation

C84 Clear and complete density one O missing at end of chain

R85 Clear and complete density Side chain mostly missing

E86 Clear and complete density one O missing at end of chain

T87 Clear and complete density Poor density some atoms missing

G88 Clear and complete density Cβ missing

S89  Clear and complete density Clear and complete density

S90 Clear and complete density Clear and complete density

K91 Weak density modelled as Very poor density, middle missing

two conformations

Y92 Clear and complete density Clear and complete density

P93 Clear and complete density Clear and complete density

N94 Clear and complete density Clear and complete density

C95 Clear and complete density Clear and complete density

A96 Clear and complete density Clear and complete density

Y97 Clear and complete density Clear and complete density

K98  Clear and complete density Density good but end two atoms missing T99 Clear and complete density Clear and complete density

T100 Double end of chain conformation Clear and complete density

Q101 Clear and complete density Clear and complete density

A102 Clear and complete density Clear and complete density

N103 Double conformation at end Clear and complete density

poor density

K104 Clear and complete density Good density but end atom missing H105 Clear and complete density Clear and complete density

I106 Clear and complete density Clear and complete density

I107 Clear and complete density Clear and complete density

V108 Clear and complete density Clear and complete density

A109 Clear and complete density Clear and complete density

C110 Clear and complete density Clear and complete density

E111 Clear and complete density Clear and complete density

G112 Clear and complete density Clear and complete density

N113 Clear and complete density V poor density even in the main chain missing atoms in side chain

P114 Clear and complete density Clear and complete density

Y115 Clear and complete density Clear and complete density

V116 Clear and complete density Clear and complete density

P117 Clear and complete density Clear and complete density

V118 Clear and complete density Clear and complete density

H119 Clear and complete density Modelled as major and minor. Minor vv weak

F120 Clear and complete density Clear and complete density

D121 Clear and complete density Clear and complete density

A122 Clear and complete density Clear and complete density S123 Clear and complete density Clear and complete density V124 Good density Clear and complete density but double conformation at end

**Notes: 1. Lysine frequently has problems. 2.The side –chains of Gln-11, His-12, Lys-41, Thr-45 and His-119 are generally recognised as being closely involved in the enzyme activity. It has also been suggested that Lys-7, Asp-44, Lys-66, Phe-120, Asp-121 and Ser-123 may also have a possible role in this mechanism. In the present orthorhombic structure the only problems with any of these residues occur for Lys-41 and Lys-66 both of which have clear density. In the monoclinic structure of Howlin *et al* [1 ] both Lys-41 and Lys-66 are again problematic as is also Lys-7.**


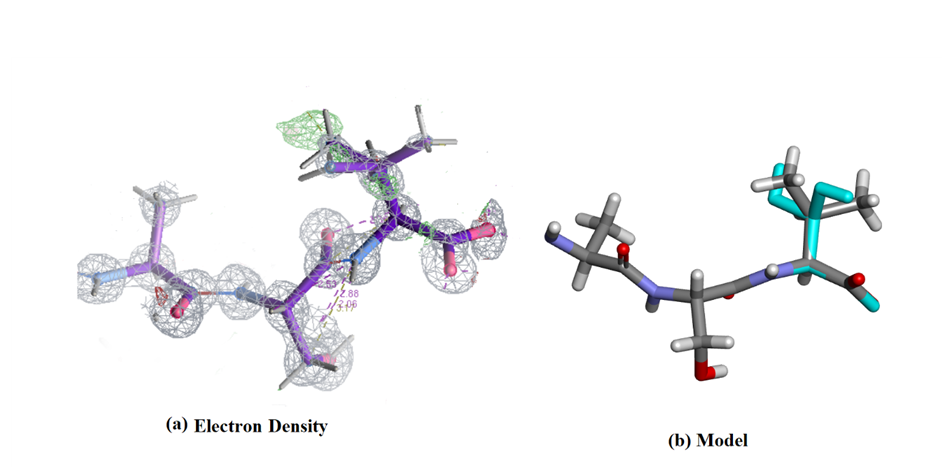


**Figure S6. The last three residues of Orthorhombic RNase A. (a) Electron density (Coot [14]) and (b) model (Biovia [17]). The electron density is** complete but Val-124 is disordered. Compare this diagram with Figure S7 corresponding to 3RN3.


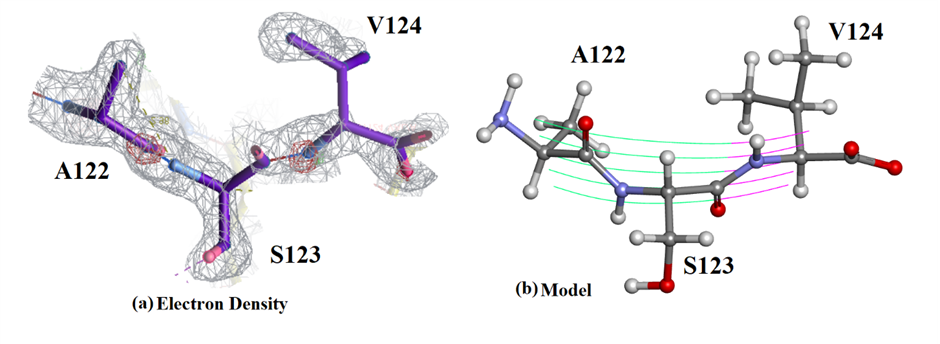


**Figure S7. The last three residues of 3RN3. (a) Electron density (Coot [14]) and (b) model (Biovia [17]). Part of the electron density is missing from the Val-124 side-chain. This is contrasted by the corresponding views of Orthorhombic RNase in Figure S6. The effect of a much higher resolution, 0.85Å compared to 1.5Å, is easy to see. The density for the orthorhombic structure is also complete for this excerpt of the structure. In (b) the coloured wavy lines indicate the course of the main chain.**

**Table S4. Comparison of PISA interfaces between Ribonuclease 3rn3 and 7p4r**

**Interfaces in PDB 3rn3 crystal.**
Space symmetry group: P 1 21 1. Resolution: 1.45 Å

| **##** | | **Structure 1** | | | | **×** | **Structure 2** | | | | | | **interface** **area, Å**^2^ | **Δ**^i^**G** **kcal/mol** | **Δ**^i^**G** **P-value** | **N**_HB_ | **N**_SB_ | **N**_DS_ | **CSS** |
| --- | --- | --- | --- | --- | --- | --- | --- | --- | --- | --- | --- | --- | --- | --- | --- | --- | --- | --- | --- |
| **NN** | **«»** | **Range** | ^i^**N**_at_ | ^i^**N**_res_ | **Surface Å**^2^ |  | **Range** | **Symmetry op-n** | **Sym.ID** | ^i^**N**_at_ | ^i^**N**_res_ | **Surface Å**^2^ |  |  |  |  |  |  |  |
| 1 |  | A | 24 | 7 | 6790 | x | A | -x+2,y-1/2,-z | 2_745 | 34 | 7 | 6790 | 229.0 | 0.3 | 0.695 | 4 | 3 | 0 | 0.000 |
| 2 |  | A | 25 | 7 | 6790 | x | A | x-1,y,z | 1_455 | 19 | 8 | 6790 | 190.2 | -2.8 | 0.269 | 0 | 0 | 0 | 0.000 |
| 3 |  | A | 14 | 4 | 6790 | x | A | -x+2,y-1/2,-z+1 | 2_746 | 23 | 9 | 6790 | 169.1 | -1.3 | 0.455 | 3 | 0 | 0 | 0.000 |
| 4 |  | A | 22 | 7 | 6790 | x | A | x,y-1,z | 1_545 | 17 | 7 | 6790 | 153.0 | -1.1 | 0.470 | 2 | 0 | 0 | 0.000 |
| 5 |  | [SO4]A:155 | 5 | 1 | 187 | f | A | x,y,z | 1_555 | 18 | 8 | 6790 | 103.8 | -14.9 | 0.792 | 4 | 0 | 0 | 0.100 |
| 6 |  | A | 12 | 4 | 6790 | x | A | -x+1,y-1/2,-z | 2_645 | 8 | 2 | 6790 | 80.8 | 0.5 | 0.597 | 1 | 0 | 0 | 0.000 |

**interface #1/6**

|  | **Structure 1** | | **Structure 2** | |
| --- | --- | --- | --- | --- |
| **Selection range** | A | | A | |
| **class** | Protein | | Protein | |
| **symmetry operation** | x,y,z | | -x+2,y-1/2,-z | |
| **symmetry ID** | 1_555 | | 2_745 | |
| **Number of atoms** |  |  |  |  |
| **interface** | 24 | 2.5% | 34 | 3.6% |
| **surface** | 552 | 58.0% | 552 | 58.0% |
| **total** | 951 | 100.0% | 951 | 100.0% |
| **Number of residues** |  |  |  |  |
| **interface** | 7 | 5.6% | 7 | 5.6% |
| **surface** | 114 | 91.9% | 114 | 91.9% |
| **total** | 124 | 100.0% | 124 | 100.0% |
| **Solvent-accessible area, Å** |  |  |  |  |
| **interface** | 234.9 | 3.5% | 223.1 | 3.3% |
| **total** | 6790.4 | 100.0% | 6790.4 | 100.0% |
| **Solvation energy, kcal/mol** |  |  |  |  |
| **isolated structure** | -92.8 | 100.0% | -92.8 | 100.0% |
| **gain on complex formation** | 0.5 | -0.5% | -0.2 | 0.2% |
| **average gain** | -0.5 | 0.6% | -0.8 | 0.8% |
| **P-value** | 0.754 |  | 0.640 |  |

**Hydrogen bonds**

| **##** | **Structure 1** | **Dist. [Å]** | **Structure 2** |
| --- | --- | --- | --- |
| 1 | A:LYS  91[ NZ ] | 2.84 | A:ASN  67[ O  ] |
| 2 | A:ARG  39[ NE ] | 3.18 | A:GLU 111[ OE2] |
| 3 | A:ASP  38[ O  ] | 3.01 | A:THR  70[ N  ] |
| 4 | A:ASP  38[ OD1] | 2.37 | A:THR  70[ OG1] |

**Salt bridges**

| **##** | **Structure 1** | **Dist. [Å]** | **Structure 2** |
| --- | --- | --- | --- |
| 1 | A:ARG  39[ NH2] | 3.44 | A:GLU 111[ OE1] |
| 2 | A:ARG  39[ NE ] | 3.18 | A:GLU 111[ OE2] |
| 3 | A:ARG  39[ NH2] | 3.65 | A:GLU 111[ OE2] |
|  |  |  |  |

**interface #2/6**

|  |  |  |  |  |  |
| --- | --- | --- | --- | --- | --- |
| **Structure 1** | **Structure 2** |  |  |  |  |
| **Selection range** | | A | | A | |
| **class** | | Protein | | Protein | |
| **symmetry operation** | | x,y,z | | x-1,y,z | |
| **symmetry ID** | | 1_555 | | 1_455 | |
| **Number of atoms** | |  |  |  |  |
| **interface** | | 25 | 2.6% | 19 | 2.0% |
| **surface** | | 552 | 58.0% | 552 | 58.0% |
| **total** | | 951 | 100.0% | 951 | 100.0% |
| **Number of residues** | |  |  |  |  |
| **interface** | | 7 | 5.6% | 8 | 6.5% |
| **surface** | | 114 | 91.9% | 114 | 91.9% |
| **total** | | 124 | 100.0% | 124 | 100.0% |
| **Solvent-accessible area, Å** | |  |  |  |  |
| **interface** | | 183.0 | 2.7% | 197.3 | 2.9% |
| **total** | | 6790.4 | 100.0% | 6790.4 | 100.0% |
| **Solvation energy, kcal/mol** | |  |  |  |  |
| **isolated structure** | | -92.8 | 100.0% | -92.8 | 100.0% |
| **gain on complex formation** | | -1.1 | 1.2% | -1.7 | 1.8% |
| **average gain** | | -0.6 | 0.6% | -0.4 | 0.5% |
| **P-value** | | 0.390 |  | 0.185 |  |

No disulfide bonds found, No covalent bonds found, No hydrogen bonds found, No salt bridges found

**interface #3/6**

|  |  |  |  |  |
| --- | --- | --- | --- | --- |
|  | **Structure 1** | | **Structure 2** | |
| **Selection range** | A | | A | |
| **class** | Protein | | Protein | |
| **symmetry operation** | x,y,z | | -x+2,y-1/2,-z+1 | |
| **symmetry ID** | 1_555 | | 2_746 | |
| **Number of atoms** |  |  |  |  |
| **interface** | 14 | 1.5% | 23 | 2.4% |
| **surface** | 552 | 58.0% | 552 | 58.0% |
| **total** | 951 | 100.0% | 951 | 100.0% |
| **Number of residues** |  |  |  |  |
| **interface** | 4 | 3.2% | 9 | 7.3% |
| **surface** | 114 | 91.9% | 114 | 91.9% |
| **total** | 124 | 100.0% | 124 | 100.0% |
| **Solvent-accessible area, Å** |  |  |  |  |
| **interface** | 180.4 | 2.7% | 157.7 | 2.3% |
| **total** | 6790.4 | 100.0% | 6790.4 | 100.0% |
| **Solvation energy, kcal/mol** |  |  |  |  |
| **isolated structure** | -92.8 | 100.0% | -92.8 | 100.0% |
| **gain on complex formation** | -0.6 | 0.6% | -0.8 | 0.8% |
| **average gain** | -0.3 | 0.3% | -0.5 | 0.6% |
| **P-value** | 0.447 |  | 0.462 |  |

**Hydrogen bonds**

| **##** | **Structure 1** | **Dist. [Å]** | **Structure 2** |
| --- | --- | --- | --- |
| 1 | A:SER  23[ N  ] | 2.86 | A:SER  15[ O  ] |
| 2 | A:ASN  24[ ND2] | 2.57 | A:SER  18[ OG ] |
| 3 | A:SER  23[ OG ] | 3.27 | A:SER  18[ N  ] |

**interface #4/6**

|  | **Structure 1** | | **Structure 2** | |
| --- | --- | --- | --- | --- |
| **Selection range** | A | | A | |
| **class** | Protein | | Protein | |
| **symmetry operation** | x,y,z | | x,y-1,z | |
| **symmetry ID** | 1_555 | | 1_545 | |
| **Number of atoms** |  |  |  |  |
| **interface** | 22 | 2.3% | 17 | 1.8% |
| **surface** | 552 | 58.0% | 552 | 58.0% |
| **total** | 951 | 100.0% | 951 | 100.0% |
| **Number of residues** |  |  |  |  |
| **interface** | 7 | 5.6% | 7 | 5.6% |
| **surface** | 114 | 91.9% | 114 | 91.9% |
| **total** | 124 | 100.0% | 124 | 100.0% |
| **Solvent-accessible area, Å** |  |  |  |  |
| **interface** | 153.9 | 2.3% | 152.1 | 2.2% |
| **total** | 6790.4 | 100.0% | 6790.4 | 100.0% |
| **Solvation energy, kcal/mol** |  |  |  |  |
| **isolated structure** | -92.8 | 100.0% | -92.8 | 100.0% |
| **gain on complex formation** | -0.3 | 0.3% | -0.9 | 0.9% |
| **average gain** | -0.5 | 0.5% | -0.4 | 0.4% |
| **P-value** | 0.585 |  | 0.378 |  |

**Hydrogen bonds**

| **##** | **Structure 1** | **Dist. [Å]** | **Structure 2** |
| --- | --- | --- | --- |
| 1 | A:SER  89[ OG ] | 3.80 | A:GLN  55[ OE1] |
| 2 | A:SER  89[ OG ] | 3.63 | A:GLN  55[ NE2] |

**interface #5/6**

|  |  |  |  |  |
| --- | --- | --- | --- | --- |
|  | **Structure 1** | | **Structure 2** | |
| **Selection range** | [SO4]A:155 | | A | |
| **class** | Ligand | | Protein | |
| **symmetry operation** | x,y,z | | x,y,z | |
| **symmetry ID** | 1_555 | | 1_555 | |
| **Number of atoms** |  |  |  |  |
| **interface** | 5 | 100.0% | 18 | 1.9% |
| **surface** | 5 | 100.0% | 552 | 58.0% |
| **total** | 5 | 100.0% | 951 | 100.0% |
| **Number of residues** |  |  |  |  |
| **interface** | 1 | 100.0% | 8 | 6.5% |
| **surface** | 1 | 100.0% | 114 | 91.9% |
| **total** | 1 | 100.0% | 124 | 100.0% |
| **Solvent-accessible area, Å** |  |  |  |  |
| **interface** | 133.2 | 71.4% | 74.5 | 1.1% |
| **total** | 186.5 | 100.0% | 6790.4 | 100.0% |
| **Solvation energy, kcal/mol** |  |  |  |  |
| **isolated structure** | 21.0 | 100.0% | -92.8 | 100.0% |
| **gain on complex formation** | -15.0 | -71.1% | 0.1 | -0.1% |
| **average gain** | -21.0 | -100.0% | -0.4 | 0.4% |
| **P-value** | 0.942 |  | 0.665 |  |

**Hydrogen bonds**

| **##** | **Structure 1** | **Dist. [Å]** | **Structure 2** |
| --- | --- | --- | --- |
| 1 | A:SO4 155[ O1 ] | 2.99 | A:HIS  12[ NE2] |
| 2 | A:SO4 155[ O2 ] | 2.82 | A:HIS  12[ NE2] |
| 3 | A:SO4 155[ O2 ] | 2.70 | A:PHE 120[ N  ] |
| 4 | A:SO4 155[ O4 ] | 2.53 | A:HIS 119[ ND1] |

**interface #6/6**

|  |  |  |  |  |
| --- | --- | --- | --- | --- |
|  | **Structure 1** | | **Structure 2** | |
| **Selection range** | A | | A | |
| **class** | Protein | | Protein | |
| **symmetry operation** | x,y,z | | -x+1,y-1/2,-z | |
| **symmetry ID** | 1_555 | | 2_645 | |
| **Number of atoms** |  |  |  |  |
| **interface** | 12 | 1.3% | 8 | 0.8% |
| **surface** | 552 | 58.0% | 552 | 58.0% |
| **total** | 951 | 100.0% | 951 | 100.0% |
| **Number of residues** |  |  |  |  |
| **interface** | 4 | 3.2% | 2 | 1.6% |
| **surface** | 114 | 91.9% | 114 | 91.9% |
| **total** | 124 | 100.0% | 124 | 100.0% |
| **Solvent-accessible area, Å** |  |  |  |  |
| **interface** | 77.1 | 1.1% | 84.5 | 1.2% |
| **total** | 6790.4 | 100.0% | 6790.4 | 100.0% |
| **Solvation energy, kcal/mol** |  |  |  |  |
| **isolated structure** | -92.8 | 100.0% | -92.8 | 100.0% |
| **gain on complex formation** | 0.9 | -1.0% | -0.4 | 0.5% |
| **average gain** | -0.3 | 0.3% | -0.2 | 0.2% |
| **P-value** | 0.858 |  | 0.416 |  |

**Hydrogen bonds**

| **##** | **Structure 1** | **Dist. [Å]** | **Structure 2** |
| --- | --- | --- | --- |
| 1 | A:LYS  37[ O  ] | 3.23 | A:LYS   1[ N  ] |
|  |  |  |  |

**Interfaces in 7p4r crystal.**
Space symmetry group: P 21 21 21. Resolution: 0.85 Å

Ultra-High Resolution X-ray Structure of Orthorhombic Bovine Pancreatic Ribonuclease at 100K

**Interfaces**

| **##** | | **Structure 1** | | | | **×** | **Structure 2** | | | | | | **interface** **area, Å**^2^ | **Δ**^i^**G** **kcal/mol** | **Δ**^i^**G** **P-value** | **N**_HB_ | **N**_SB_ | **N**_DS_ | **CSS** |
| --- | --- | --- | --- | --- | --- | --- | --- | --- | --- | --- | --- | --- | --- | --- | --- | --- | --- | --- | --- |
| **NN** | **«»** | **Range** | ^i^**N**_at_ | ^i^**N**_res_ | **Surface Å**^2^ |  | **Range** | **Symmetry op-n** | **Sym.ID** | ^i^**N**_at_ | ^i^**N**_res_ | **Surface Å**^2^ |  |  |  |  |  |  |  |
| 1 |  | A | 65 | 16 | 6957 | x | A | x-1/2,-y+1/2,-z | **3**_455 | 58 | 19 | 6957 | 492.7 | -1.1 | 0.541 | 0 | 0 | 0 | 0.000 |
| 2 |  | A | 42 | 13 | 6957 | x | A | -x+1/2,-y,z-1/2 | **2**_554 | 35 | 14 | 6957 | 335.0 | -1.2 | 0.507 | 0 | 0 | 0 | 0.000 |
| 3 |  | A | 30 | 8 | 6957 | x | A | -x,y-1/2,-z+1/2 | **4**_545 | 36 | 14 | 6957 | 296.0 | 1.9 | 0.650 | 0 | 0 | 0 | 0.000 |
| 4 |  | A | 15 | 6 | 6957 | x | A | -x+1,y-1/2,-z+1/2 | **4**_645 | 20 | 7 | 6957 | 148.4 | -0.7 | 0.512 | 0 | 0 | 0 | 0.000 |

Plus 66 other intermolecular links involving the ligands ie SO4 or Ethanol

**interface #1/70**

|  |  |  |  |  |
| --- | --- | --- | --- | --- |
|  | **Structure 1** | | **Structure 2** | |
| **Selection range** | AA | | AA | |
| **class** | Protein | | Protein | |
| **symmetry operation** | x,y,z | | x-1/2,-y+1/2,-z | |
| **symmetry ID** | 1_555 | | 2_455 | |
| **Number of atoms** |  |  |  |  |
| **interface** | 65 | 6.8% | 58 | 6.1% |
| **surface** | 564 | 59.3% | 564 | 59.3% |
| **total** | 951 | 100.0% | 951 | 100.0% |
| **Number of residues** |  |  |  |  |
| **interface** | 16 | 12.9% | 19 | 15.3% |
| **surface** | 115 | 92.7% | 115 | 92.7% |
| **total** | 124 | 100.0% | 124 | 100.0% |
| **Solvent-accessible area, Å** |  |  |  |  |
| **interface** | 461.1 | 6.6% | 524.4 | 7.5% |
| **total** | 6957.1 | 100.0% | 6957.1 | 100.0% |
| **Solvation energy, kcal/mol** |  |  |  |  |
| **isolated structure** | -94.2 | 100.0% | -94.2 | 100.0% |
| **gain on complex formation** | 0.9 | -1.0% | -2.0 | 2.1% |
| **average gain** | -1.2 | 1.3% | -1.1 | 1.1% |
| **P-value** | 0.804 |  | 0.363 |  |

**Hydrogen bonds**

| **##** | **Structure 1** | **Dist. [Å]** | **Structure 2** |
| --- | --- | --- | --- |
| 1 | AA:SER  15[ H  ] | 2.45 | AA:TYR  76[ OH ] |
| 2 | AA:ARG  33[HH21] | 1.92 | AA:SER  59[ O  ] |
| 3 | AA:ARG  33[HH22] | 2.43 | AA:TYR  76[ OH ] |
| 4 | AA:ASN  34[HD22] | 2.21 | AA:ASP  53[ OD1] |
| 5 | AA:MET  13[ O  ] | 2.94 | AA:TYR  76[ OH ] |

**Salt bridges**

| **##** | **Structure 1** | **Dist. [Å]** | **Structure 2** |
| --- | --- | --- | --- |
| 1 | AA:ARG  10[ NE ] | 3.35 | AA:ASP  53[ OD1] |

**interface #2/70**

|  | **Structure 1** | | **Structure 2** | |
| --- | --- | --- | --- | --- |
| **Selection range** | AA | | AA | |
| **class** | Protein | | Protein | |
| **symmetry operation** | x,y,z | | -x+1/2,-y,z-1/2 | |
| **symmetry ID** | 1_555 | | 1_554 | |
| **Number of atoms** |  |  |  |  |
| **interface** | 42 | 4.4% | 35 | 3.7% |
| **surface** | 564 | 59.3% | 564 | 59.3% |
| **total** | 951 | 100.0% | 951 | 100.0% |
| **Number of residues** |  |  |  |  |
| **interface** | 13 | 10.5% | 14 | 11.3% |
| **surface** | 115 | 92.7% | 115 | 92.7% |
| **total** | 124 | 100.0% | 124 | 100.0% |
| **Solvent-accessible area, Å** |  |  |  |  |
| **interface** | 331.8 | 4.8% | 338.1 | 4.9% |
| **total** | 6957.1 | 100.0% | 6957.1 | 100.0% |
| **Solvation energy, kcal/mol** |  |  |  |  |
| **isolated structure** | -94.2 | 100.0% | -94.2 | 100.0% |
| **gain on complex formation** | -1.5 | 1.5% | 0.2 | -0.3% |
| **average gain** | -0.8 | 0.8% | -0.6 | 0.7% |
| **P-value** | 0.381 |  | 0.674 |  |

**Hydrogen bonds**

| **##** | **Structure 1** | **Dist. [Å]** | **Structure 2** |
| --- | --- | --- | --- |
| 1 | AA:ASN 113[HD22] | 2.15 | AA:VAL  43[ O  ] |
| 2 | AA:THR  70[ OG1] | 3.31 | AA:GLY  88[ O  ] |
| 3 | AA:THR  70[ H  ] | 2.12 | AA:GLY  88[ O ] |

**interface #3/70**

|  |  |  |  |  |
| --- | --- | --- | --- | --- |
|  | **Structure 1** | | **Structure 2** | |
| **Selection range** | AA | | AA | |
| **class** | Protein | | Protein | |
| **symmetry operation** | x,y,z | | -x,y-1/2,-z+1/2 | |
| **symmetry ID** | 1_555 | | 3_545 | |
| **Number of atoms** |  |  |  |  |
| **interface** | 30 | 3.2% | 36 | 3.8% |
| **surface** | 564 | 59.3% | 564 | 59.3% |
| **total** | 951 | 100.0% | 951 | 100.0% |
| **Number of residues** |  |  |  |  |
| **interface** | 8 | 6.5% | 14 | 11.3% |
| **surface** | 115 | 92.7% | 115 | 92.7% |
| **total** | 124 | 100.0% | 124 | 100.0% |
| **Solvent-accessible area, Å** |  |  |  |  |
| **interface** | 329.7 | 4.7% | 262.3 | 3.8% |
| **total** | 6957.1 | 100.0% | 6957.1 | 100.0% |
| **Solvation energy, kcal/mol** |  |  |  |  |
| **isolated structure** | -94.2 | 100.0% | -94.2 | 100.0% |
| **gain on complex formation** | 3.2 | -3.4% | -1.2 | 1.3% |
| **average gain** | -0.5 | 0.6% | -0.7 | 0.7% |
| **P-value** | 0.965 |  | 0.438 |  |

**Hydrogen bonds**

| **##** | **Structure 1** | **Dist. [Å]** | **Structure 2** |
| --- | --- | --- | --- |
| 1 | AA:LYS  37[ HZ3] | 2.37 | AA:SER  21[ O  ] |
| 2 | AA:ARG  39[HH22] | 2.01 | AA:ASP  14[ OD1] |
| 3 | AA:ASP  38[ OD1] | 2.68 | AA:SER  21[ OG] |

**Salt bridges**

| **##** | **Structure 1** | **Dist. [Å]** | **Structure 2** |
| --- | --- | --- | --- |
| 1 | AA:ARG  39[ NH1] | 3.47 | AA:ASP  14[ OD2] |
| 2 | AA:ARG  39[ NH2] | 3.53 | AA:ASP  14[ OD2] |
| 3 | AA:ARG  39[ NH2] | 2.77 | AA:ASP  14[ OD1] |

**interface #4/70**

| **Structure 1** | **Structure 2** |  |  |  |  |
| --- | --- | --- | --- | --- | --- |
| **Selection range** | | AA | | AA | |
| **class** | | Protein | | Protein | |
| **symmetry operation** | | x,y,z | | -x+1,y-1/2,-z+1/2 | |
| **symmetry ID** | | 1_555 | | 3_645 | |
| **Number of atoms** | |  |  |  |  |
| **interface** | | 15 | 1.6% | 20 | 2.1% |
| **surface** | | 564 | 59.3% | 564 | 59.3% |
| **total** | | 951 | 100.0% | 951 | 100.0% |
| **Number of residues** | |  |  |  |  |
| **interface** | | 6 | 4.8% | 7 | 5.6% |
| **surface** | | 115 | 92.7% | 115 | 92.7% |
| **total** | | 124 | 100.0% | 124 | 100.0% |
| **Solvent-accessible area, Å** | |  |  |  |  |
| **interface** | | 157.3 | 2.3% | 139.5 | 2.0% |
| **total** | | 6957.1 | 100.0% | 6957.1 | 100.0% |
| **Solvation energy, kcal/mol** | |  |  |  |  |
| **isolated structure** | | -94.2 | 100.0% | -94.2 | 100.0% |
| **gain on complex formation** | | -0.2 | 0.2% | -0.5 | 0.6% |
| **average gain** | | -0.3 | 0.3% | -0.4 | 0.4% |
| **P-value** | | 0.538 |  | 0.487 |  |

For both forms: Analysis of the protein interfaces has not revealed any specific interactions that could result in the formation of stable quaternary structures.

Most probably, the structures do not form a complex in solution.

All the above stats were generated by using PDBe PISA v1.52 [20/10/2014] on the EBI Web server.

**Table S5 MD For Lys-41**

| C-CA-CB-CG |  | CA-CB-CG-CD | CB-CG-CD-CE | CG-CD-CE-NZ | | A | B | A | B |  | B | A | B |  | RN3 |  |  |  |  |  |  |  |  |  |  |  |  |  |  |  |
| --- | --- | --- | --- | --- | --- | --- | --- | --- | --- | --- | --- | --- | --- | --- | --- | --- | --- | --- | --- | --- | --- | --- | --- | --- | --- | --- | --- | --- | --- | --- |
| 54.58169 |  | 175.6994 | 175.0251 | -93.9457 |  | 60.76 | 132.64 | 175.01 | -143.13 | -177.97 | -43.5 | 170.61 | -175.04 |  | 132.64 | -143.13 | -43.5 | -175.04 |  |  |  |  |  |  |  |  |  |  |  |  |
| 65.55544 |  | 177.82 | -163.159 | -83.2331 |  | 60.76 | 132.64 | 175.01 | -143.13 | -177.97 | -43.5 | 170.61 | -175.04 |  |  |  |  |  |  |  |  |  |  |  |  |  |  |  |  |  |
| 68.06978 |  | 173.4704 | -171.853 | -97.1893 |  | 60.76 | 132.64 | 175.01 | -143.13 | -177.97 | -43.5 | 170.61 | -175.04 |  | | | | | | | | | | | | | | | | |
| 83.62248 |  | 176.9016 | 175.5179 | -91.2196 |  | 60.76 | 132.64 | 175.01 | -143.13 | -177.97 | -43.5 | 170.61 | -175.04 |  |  |  |  |  |  |  |  |  |  |  |  |  |  |  |  |  |
| 66.01351 |  | 169.9173 | -161.789 | -68.7005 |  | 60.76 | 132.64 | 175.01 | -143.13 | -177.97 | -43.5 | 170.61 | -175.04 |  |  |  |  |  |  |  |  |  |  |  |  |  |  |  |  |  |
| 75.59292 |  | 173.1697 | -171.78 | -97.8377 |  | 60.76 | 132.64 | 175.01 | -143.13 | -177.97 | -43.5 | 170.61 | -175.04 |  |  |  |  |  |  |  |  |  |  |  |  |  |  |  |  |  |
| 56.26902 |  | 170.4412 | -179.067 | -78.8421 |  | 60.76 | 132.64 | 175.01 | -143.13 | -177.97 | -43.5 | 170.61 | -175.04 |  |  |  |  |  |  |  |  |  |  |  |  |  |  |  |  |  |
| 64.36303 |  | 171.8756 | 179.919 | -84.5376 |  | 60.76 | 132.64 | 175.01 | -143.13 | -177.97 | -43.5 | 170.61 | -175.04 |  |  |  |  |  |  |  |  |  |  |  |  |  |  |  |  |  |
| 76.3103 |  | 168.6847 | 177.2181 | -68.3003 |  | 60.76 | 132.64 | 175.01 | -143.13 | -177.97 | -43.5 | 170.61 | -175.04 |  |  |  |  |  |  |  |  |  |  |  |  |  |  |  |  |  |
| 68.82958 |  | 165.221 | 179.637 | -76.1323 |  | 60.76 | 132.64 | 175.01 | -143.13 | -177.97 | -43.5 | 170.61 | -175.04 |  |  |  |  |  |  |  |  |  |  |  |  |  |  |  |  |  |
| 63.40079 |  | 174.346 | -167.866 | -95.2621 |  | 60.76 | 132.64 | 175.01 | -143.13 | -177.97 | -43.5 | 170.61 | -175.04 |  |  |  |  |  |  |  |  |  |  |  |  |  |  |  |  |  |
| 68.94984 |  | 167.4895 | -179.593 | -76.488 |  | 60.76 | 132.64 | 175.01 | -143.13 | -177.97 | -43.5 | 170.61 | -175.04 |  |  |  |  |  |  |  |  |  |  |  |  |  |  |  |  |  |
| 64.37743 |  | 172.8463 | -171.999 | -72.8285 |  | 60.76 | 132.64 | 175.01 | -143.13 | -177.97 | -43.5 | 170.61 | -175.04 |  |  |  |  |  |  |  |  |  |  |  |  |  |  |  |  |  |
| 62.20957 |  | -171.928 | 179.315 | -98.1099 |  | 60.76 | 132.64 | 175.01 | -143.13 | -177.97 | -43.5 | 170.61 | -175.04 |  |  |  |  |  |  |  |  |  |  |  |  |  |  |  |  |  |
| 66.6338 |  | -177.835 | -179.211 | -85.251 |  | 60.76 | 132.64 | 175.01 | -143.13 | -177.97 | -43.5 | 170.61 | -175.04 |  |  |  |  |  |  |  |  |  |  |  |  |  |  |  |  |  |
| 76.29916 |  | 167.2723 | 175.9471 | -84.4015 |  | 60.76 | 132.64 | 175.01 | -143.13 | -177.97 | -43.5 | 170.61 | -175.04 |  |  |  |  |  |  |  |  |  |  |  |  |  |  |  |  |  |
| 60.45237 |  | 166.1179 | -175.545 | -57.2791 |  | 60.76 | 132.64 | 175.01 | -143.13 | -177.97 | -43.5 | 170.61 | -175.04 |  |  |  |  |  |  |  |  |  |  |  |  |  |  |  |  |  |
| 63.74891 |  | 164.7692 | -171.683 | -89.4129 |  | 60.76 | 132.64 | 175.01 | -143.13 | -177.97 | -43.5 | 170.61 | -175.04 |  |  |  |  |  |  |  |  |  |  |  |  |  |  |  |  |  |
| 70.91924 |  | 154.6507 | -174.651 | -77.0713 |  | 60.76 | 132.64 | 175.01 | -143.13 | -177.97 | -43.5 | 170.61 | -175.04 |  |  |  |  |  |  |  |  |  |  |  |  |  |  |  |  |  |
| 53.89794 |  | 169.5462 | 178.9759 | -70.0819 |  | 60.76 | 132.64 | 175.01 | -143.13 | -177.97 | -43.5 | 170.61 | -175.04 |  |  |  |  |  |  |  |  |  |  |  |  |  |  |  |  |  |
| 79.94112 |  | 179.0931 | 177.7789 | -88.8721 |  | 60.76 | 132.64 | 175.01 | -143.13 | -177.97 | -43.5 | 170.61 | -175.04 |  |  |  |  |  |  |  |  |  |  |  |  |  |  |  |  |  |
| 64.5267 |  | 166.1826 | -174.37 | -72.7625 |  | 60.76 | 132.64 | 175.01 | -143.13 | -177.97 | -43.5 | 170.61 | -175.04 |  |  |  |  |  |  |  |  |  |  |  |  |  |  |  |  |  |
| 72.19032 |  | 173.3999 | -164.48 | -82.3553 |  | 60.76 | 132.64 | 175.01 | -143.13 | -177.97 | -43.5 | 170.61 | -175.04 |  |  |  |  |  |  |  |  |  |  |  |  |  |  |  |  |  |
| 70.18359 |  | 179.8584 | 175.2831 | -91.0744 |  | 60.76 | 132.64 | 175.01 | -143.13 | -177.97 | -43.5 | 170.61 | -175.04 |  |  |  |  |  |  |  |  |  |  |  |  |  |  |  |  |  |
| 65.60604 |  | 170.3221 | 168.231 | -67.521 |  | 60.76 | 132.64 | 175.01 | -143.13 | -177.97 | -43.5 | 170.61 | -175.04 |  |  |  |  |  |  |  |  |  |  |  |  |  |  |  |  |  |
| 73.92185 |  | 161.1304 | 171.8425 | -84.7503 |  | 60.76 | 132.64 | 175.01 | -143.13 | -177.97 | -43.5 | 170.61 | -175.04 |  |  |  |  |  |  |  |  |  |  |  |  |  |  |  |  |  |
| 71.19894 |  | 165.6356 | -175.001 | -65.1958 |  | 60.76 | 132.64 | 175.01 | -143.13 | -177.97 | -43.5 | 170.61 | -175.04 |  |  |  |  |  |  |  |  |  |  |  |  |  |  |  |  |  |
| 65.39854 |  | 163.476 | 174.3391 | -81.1036 |  | 60.76 | 132.64 | 175.01 | -143.13 | -177.97 | -43.5 | 170.61 | -175.04 |  |  |  |  |  |  |  |  |  |  |  |  |  |  |  |  |  |
| 60.51816 |  | -174.545 | -172.274 | -83.4251 |  | 60.76 | 132.64 | 175.01 | -143.13 | -177.97 | -43.5 | 170.61 | -175.04 |  |  |  |  |  |  |  |  |  |  |  |  |  |  |  |  |  |
| 76.5303 |  | 170.6277 | -176.239 | -84.6599 |  | 60.76 | 132.64 | 175.01 | -143.13 | -177.97 | -43.5 | 170.61 | -175.04 |  |  |  |  |  |  |  |  |  |  |  |  |  |  |  |  |  |
| 78.56857 |  | -174.458 | -172.008 | -105.723 |  | 60.76 | 132.64 | 175.01 | -143.13 | -177.97 | -43.5 | 170.61 | -175.04 |  |  |  |  |  |  |  |  |  |  |  |  |  |  |  |  |  |
| 67.10004 |  | 159.7704 | 166.8606 | -100.987 |  | 60.76 | 132.64 | 175.01 | -143.13 | -177.97 | -43.5 | 170.61 | -175.04 |  |  |  |  |  |  |  |  |  |  |  |  |  |  |  |  |  |
| 67.52151 |  | 172.6974 | 175.2203 | -111.684 |  | 60.76 | 132.64 | 175.01 | -143.13 | -177.97 | -43.5 | 170.61 | -175.04 |  |  |  |  |  |  |  |  |  |  |  |  |  |  |  |  |  |
| 82.62222 |  | 166.8644 | 167.2631 | -86.6312 |  | 60.76 | 132.64 | 175.01 | -143.13 | -177.97 | -43.5 | 170.61 | -175.04 |  |  |  |  |  |  |  |  |  |  |  |  |  |  |  |  |  |
| 82.69967 |  | 178.3232 | 177.6274 | -99.1173 |  | 60.76 | 132.64 | 175.01 | -143.13 | -177.97 | -43.5 | 170.61 | -175.04 |  |  |  |  |  |  |  |  |  |  |  |  |  |  |  |  |  |
| 73.62487 |  | 167.4484 | 175.4008 | -90.5004 |  | 60.76 | 132.64 | 175.01 | -143.13 | -177.97 | -43.5 | 170.61 | -175.04 |  |  |  |  |  |  |  |  |  |  |  |  |  |  |  |  |  |
| 65.98914 |  | -168.859 | -159.393 | -110.014 |  | 60.76 | 132.64 | 175.01 | -143.13 | -177.97 | -43.5 | 170.61 | -175.04 |  |  |  |  |  |  |  |  |  |  |  |  |  |  |  |  |  |
| 77.21452 |  | 177.6354 | 172.4325 | -103.213 |  | 60.76 | 132.64 | 175.01 | -143.13 | -177.97 | -43.5 | 170.61 | -175.04 |  |  |  |  |  |  |  |  |  |  |  |  |  |  |  |  |  |
| 33.45079 |  | -175.509 | -174.43 | -90.0829 |  | 60.76 | 132.64 | 175.01 | -143.13 | -177.97 | -43.5 | 170.61 | -175.04 |  |  |  |  |  |  |  |  |  |  |  |  |  |  |  |  |  |
| 64.99759 |  | 176.0298 | -171.407 | -95.1574 |  | 60.76 | 132.64 | 175.01 | -143.13 | -177.97 | -43.5 | 170.61 | -175.04 |  |  |  |  |  |  |  |  |  |  |  |  |  |  |  |  |  |
| 69.2588 |  | 166.4173 | 179.1099 | -83.5333 |  | 60.76 | 132.64 | 175.01 | -143.13 | -177.97 | -43.5 | 170.61 | -175.04 |  |  |  |  |  |  |  |  |  |  |  |  |  |  |  |  |  |
| 66.91754 |  | 177.1807 | 171.3219 | -95.1008 |  | 60.76 | 132.64 | 175.01 | -143.13 | -177.97 | -43.5 | 170.61 | -175.04 |  |  |  |  |  |  |  |  |  |  |  |  |  |  |  |  |  |
| 60.82489 |  | 171.6677 | 178.8515 | -82.1115 |  | 60.76 | 132.64 | 175.01 | -143.13 | -177.97 | -43.5 | 170.61 | -175.04 |  |  |  |  |  |  |  |  |  |  |  |  |  |  |  |  |  |
| 71.05885 |  | 148.012 | -175.481 | -78.3673 |  | 60.76 | 132.64 | 175.01 | -143.13 | -177.97 | -43.5 | 170.61 | -175.04 |  |  |  |  |  |  |  |  |  |  |  |  |  |  |  |  |  |
| 70.75826 |  | 169.4226 | 162.9723 | -95.6852 |  | 60.76 | 132.64 | 175.01 | -143.13 | -177.97 | -43.5 | 170.61 | -175.04 |  |  |  |  |  |  |  |  |  |  |  |  |  |  |  |  |  |
| 72.0741 |  | -179.528 | -175.548 | -104.512 |  | 60.76 | 132.64 | 175.01 | -143.13 | -177.97 | -43.5 | 170.61 | -175.04 |  |  |  |  |  |  |  |  |  |  |  |  |  |  |  |  |  |
| 44.41869 |  | 169.8338 | -152.43 | -95.0363 |  | 60.76 | 132.64 | 175.01 | -143.13 | -177.97 | -43.5 | 170.61 | -175.04 |  |  |  |  |  |  |  |  |  |  |  |  |  |  |  |  |  |
| 71.32714 |  | 174.1175 | -174.52 | -85.3897 |  | 60.76 | 132.64 | 175.01 | -143.13 | -177.97 | -43.5 | 170.61 | -175.04 |  |  |  |  |  |  |  |  |  |  |  |  |  |  |  |  |  |
| 71.06393 |  | 176.533 | -176.115 | -166.441 |  | 60.76 | 132.64 | 175.01 | -143.13 | -177.97 | -43.5 | 170.61 | -175.04 |  |  |  |  |  |  |  |  |  |  |  |  |  |  |  |  |  |
| 59.77693 |  | 178.6117 | -177.415 | -179.55 |  | 60.76 | 132.64 | 175.01 | -143.13 | -177.97 | -43.5 | 170.61 | -175.04 |  |  |  |  |  |  |  |  |  |  |  |  |  |  |  |  |  |
| 55.32205 |  | -177.125 | -175.89 | -173.476 |  | 60.76 | 132.64 | 175.01 | -143.13 | -177.97 | -43.5 | 170.61 | -175.04 |  |  |  |  |  |  |  |  |  |  |  |  |  |  |  |  |  |
| 76.40952 |  | 176.7609 | 169.5163 | -159.911 |  | 60.76 | 132.64 | 175.01 | -143.13 | -177.97 | -43.5 | 170.61 | -175.04 |  |  |  |  |  |  |  |  |  |  |  |  |  |  |  |  |  |
| 72.37405 |  | 172.025 | 177.6476 | -173.78 |  | 60.76 | 132.64 | 175.01 | -143.13 | -177.97 | -43.5 | 170.61 | -175.04 |  |  |  |  |  |  |  |  |  |  |  |  |  |  |  |  |  |
| 66.49053 |  | 167.0757 | -175.665 | -80.9558 |  | 60.76 | 132.64 | 175.01 | -143.13 | -177.97 | -43.5 | 170.61 | -175.04 |  |  |  |  |  |  |  |  |  |  |  |  |  |  |  |  |  |
| 54.53233 |  | 176.9167 | -152.843 | -92.1841 |  | 60.76 | 132.64 | 175.01 | -143.13 | -177.97 | -43.5 | 170.61 | -175.04 |  |  |  |  |  |  |  |  |  |  |  |  |  |  |  |  |  |
| 81.04482 |  | 168.7291 | 176.397 | -79.9651 |  | 60.76 | 132.64 | 175.01 | -143.13 | -177.97 | -43.5 | 170.61 | -175.04 |  |  |  |  |  |  |  |  |  |  |  |  |  |  |  |  |  |
| 63.40587 |  | 179.8063 | -166.595 | -75.6034 |  | 60.76 | 132.64 | 175.01 | -143.13 | -177.97 | -43.5 | 170.61 | -175.04 |  |  |  |  |  |  |  |  |  |  |  |  |  |  |  |  |  |
| 76.25713 |  | 164.3684 | -175.469 | -97.8912 |  | 60.76 | 132.64 | 175.01 | -143.13 | -177.97 | -43.5 | 170.61 | -175.04 |  |  |  |  |  |  |  |  |  |  |  |  |  |  |  |  |  |
| 76.79762 |  | -171.583 | 160.512 | -170.14 |  | 60.76 | 132.64 | 175.01 | -143.13 | -177.97 | -43.5 | 170.61 | -175.04 |  |  |  |  |  |  |  |  |  |  |  |  |  |  |  |  |  |
| 61.72213 |  | 173.6765 | -175.832 | -92.2099 |  | 60.76 | 132.64 | 175.01 | -143.13 | -177.97 | -43.5 | 170.61 | -175.04 |  |  |  |  |  |  |  |  |  |  |  |  |  |  |  |  |  |
| 72.31192 |  | 174.5703 | 177.7999 | -103.887 |  | 60.76 | 132.64 | 175.01 | -143.13 | -177.97 | -43.5 | 170.61 | -175.04 |  |  |  |  |  |  |  |  |  |  |  |  |  |  |  |  |  |
| 60.22483 |  | -175.928 | -172.893 | -86.8204 |  | 60.76 | 132.64 | 175.01 | -143.13 | -177.97 | -43.5 | 170.61 | -175.04 |  |  |  |  |  |  |  |  |  |  |  |  |  |  |  |  |  |
| 62.40728 |  | 169.8381 | -167.866 | -86.72 |  | 60.76 | 132.64 | 175.01 | -143.13 | -177.97 | -43.5 | 170.61 | -175.04 |  |  |  |  |  |  |  |  |  |  |  |  |  |  |  |  |  |
| 66.12922 |  | 171.1947 | -175.794 | -87.7225 |  | 60.76 | 132.64 | 175.01 | -143.13 | -177.97 | -43.5 | 170.61 | -175.04 |  |  |  |  |  |  |  |  |  |  |  |  |  |  |  |  |  |
| 68.34998 |  | 175.0003 | -173.106 | -102.132 |  | 60.76 | 132.64 | 175.01 | -143.13 | -177.97 | -43.5 | 170.61 | -175.04 |  |  |  |  |  |  |  |  |  |  |  |  |  |  |  |  |  |
| 60.95637 |  | 175.7762 | -177.16 | -78.4347 |  | 60.76 | 132.64 | 175.01 | -143.13 | -177.97 | -43.5 | 170.61 | -175.04 |  |  |  |  |  |  |  |  |  |  |  |  |  |  |  |  |  |
| 67.94522 |  | 165.7481 | -173.506 | -90.571 |  | 60.76 | 132.64 | 175.01 | -143.13 | -177.97 | -43.5 | 170.61 | -175.04 |  |  |  |  |  |  |  |  |  |  |  |  |  |  |  |  |  |
| 57.1723 |  | 176.5898 | -173.452 | -70.0332 |  | 60.76 | 132.64 | 175.01 | -143.13 | -177.97 | -43.5 | 170.61 | -175.04 |  |  |  |  |  |  |  |  |  |  |  |  |  |  |  |  |  |
| 70.111 |  | 172.114 | 166.1799 | -77.7115 |  | 60.76 | 132.64 | 175.01 | -143.13 | -177.97 | -43.5 | 170.61 | -175.04 |  |  |  |  |  |  |  |  |  |  |  |  |  |  |  |  |  |
| 48.40363 |  | -176.222 | -169.824 | -82.1711 |  | 60.76 | 132.64 | 175.01 | -143.13 | -177.97 | -43.5 | 170.61 | -175.04 |  |  |  |  |  |  |  |  |  |  |  |  |  |  |  |  |  |
| 69.72627 |  | 167.9584 | -172.992 | -72.2145 |  | 60.76 | 132.64 | 175.01 | -143.13 | -177.97 | -43.5 | 170.61 | -175.04 |  |  |  |  |  |  |  |  |  |  |  |  |  |  |  |  |  |
| 62.35901 |  | 176.8571 | -176.939 | -90.0976 |  | 60.76 | 132.64 | 175.01 | -143.13 | -177.97 | -43.5 | 170.61 | -175.04 |  |  |  |  |  |  |  |  |  |  |  |  |  |  |  |  |  |
| 68.232 |  | 174.1586 | -177.67 | -86.6799 |  | 60.76 | 132.64 | 175.01 | -143.13 | -177.97 | -43.5 | 170.61 | -175.04 |  |  |  |  |  |  |  |  |  |  |  |  |  |  |  |  |  |
| 66.50056 |  | 164.7873 | 173.0329 | -78.0127 |  | 60.76 | 132.64 | 175.01 | -143.13 | -177.97 | -43.5 | 170.61 | -175.04 |  |  |  |  |  |  |  |  |  |  |  |  |  |  |  |  |  |
| 71.19101 |  | 157.66 | -178.782 | -72.2984 |  | 60.76 | 132.64 | 175.01 | -143.13 | -177.97 | -43.5 | 170.61 | -175.04 |  |  |  |  |  |  |  |  |  |  |  |  |  |  |  |  |  |
| 70.77644 |  | -178.82 | -179.138 | -83.0819 |  | 60.76 | 132.64 | 175.01 | -143.13 | -177.97 | -43.5 | 170.61 | -175.04 |  |  |  |  |  |  |  |  |  |  |  |  |  |  |  |  |  |
| 60.91307 |  | 171.672 | -176.648 | -84.2188 |  | 60.76 | 132.64 | 175.01 | -143.13 | -177.97 | -43.5 | 170.61 | -175.04 |  |  |  |  |  |  |  |  |  |  |  |  |  |  |  |  |  |
| 65.27776 |  | 158.176 | 177.322 | -69.4548 |  | 60.76 | 132.64 | 175.01 | -143.13 | -177.97 | -43.5 | 170.61 | -175.04 |  |  |  |  |  |  |  |  |  |  |  |  |  |  |  |  |  |
| 62.96227 |  | 171.7693 | -172.353 | -63.2066 |  | 60.76 | 132.64 | 175.01 | -143.13 | -177.97 | -43.5 | 170.61 | -175.04 |  |  |  |  |  |  |  |  |  |  |  |  |  |  |  |  |  |
| 66.56477 |  | 172.5469 | 176.2744 | -85.2267 |  | 60.76 | 132.64 | 175.01 | -143.13 | -177.97 | -43.5 | 170.61 | -175.04 |  |  |  |  |  |  |  |  |  |  |  |  |  |  |  |  |  |
| 62.86177 |  | 160.4363 | -176.713 | -74.8353 |  | 60.76 | 132.64 | 175.01 | -143.13 | -177.97 | -43.5 | 170.61 | -175.04 |  |  |  |  |  |  |  |  |  |  |  |  |  |  |  |  |  |
| 56.59439 |  | -176.03 | -169.219 | -97.3984 |  | 60.76 | 132.64 | 175.01 | -143.13 | -177.97 | -43.5 | 170.61 | -175.04 |  |  |  |  |  |  |  |  |  |  |  |  |  |  |  |  |  |
| 73.97842 |  | 179.9518 | 175.8516 | -99.9641 |  | 60.76 | 132.64 | 175.01 | -143.13 | -177.97 | -43.5 | 170.61 | -175.04 |  |  |  |  |  |  |  |  |  |  |  |  |  |  |  |  |  |
| 78.95255 |  | 165.6451 | 176.7983 | -91.3072 |  | 60.76 | 132.64 | 175.01 | -143.13 | -177.97 | -43.5 | 170.61 | -175.04 |  |  |  |  |  |  |  |  |  |  |  |  |  |  |  |  |  |
| 69.20857 |  | 160.3372 | 169.7574 | -76.6803 |  | 60.76 | 132.64 | 175.01 | -143.13 | -177.97 | -43.5 | 170.61 | -175.04 |  |  |  |  |  |  |  |  |  |  |  |  |  |  |  |  |  |
| 56.44745 |  | 175.1069 | -174.844 | -105.237 |  | 60.76 | 132.64 | 175.01 | -143.13 | -177.97 | -43.5 | 170.61 | -175.04 |  |  |  |  |  |  |  |  |  |  |  |  |  |  |  |  |  |
| 78.56549 |  | -176.92 | 163.8216 | 177.2621 |  | 60.76 | 132.64 | 175.01 | -143.13 | -177.97 | -43.5 | 170.61 | -175.04 |  |  |  |  |  |  |  |  |  |  |  |  |  |  |  |  |  |
| 66.73845 |  | -177.713 | 167.0028 | -165.823 |  | 60.76 | 132.64 | 175.01 | -143.13 | -177.97 | -43.5 | 170.61 | -175.04 |  |  |  |  |  |  |  |  |  |  |  |  |  |  |  |  |  |
| 79.86969 |  | -179.903 | 166.7963 | -178.221 |  | 60.76 | 132.64 | 175.01 | -143.13 | -177.97 | -43.5 | 170.61 | -175.04 |  |  |  |  |  |  |  |  |  |  |  |  |  |  |  |  |  |
| 64.60264 |  | 169.9279 | 178.5578 | -167.99 |  | 60.76 | 132.64 | 175.01 | -143.13 | -177.97 | -43.5 | 170.61 | -175.04 |  |  |  |  |  |  |  |  |  |  |  |  |  |  |  |  |  |
| 69.91919 |  | 173.9784 | 179.3383 | -170.23 |  | 60.76 | 132.64 | 175.01 | -143.13 | -177.97 | -43.5 | 170.61 | -175.04 |  |  |  |  |  |  |  |  |  |  |  |  |  |  |  |  |  |
| 78.43949 |  | -177.878 | 163.0904 | 179.9971 |  | 60.76 | 132.64 | 175.01 | -143.13 | -177.97 | -43.5 | 170.61 | -175.04 |  |  |  |  |  |  |  |  |  |  |  |  |  |  |  |  |  |
| 66.16373 |  | -177.075 | 164.8829 | 179.2485 |  | 60.76 | 132.64 | 175.01 | -143.13 | -177.97 | -43.5 | 170.61 | -175.04 |  |  |  |  |  |  |  |  |  |  |  |  |  |  |  |  |  |
| 56.91754 |  | 178.2994 | -170.538 | -176.673 |  | 60.76 | 132.64 | 175.01 | -143.13 | -177.97 | -43.5 | 170.61 | -175.04 |  |  |  |  |  |  |  |  |  |  |  |  |  |  |  |  |  |
| 50.93956 |  | 170.4615 | 167.6037 | -166.622 |  | 60.76 | 132.64 | 175.01 | -143.13 | -177.97 | -43.5 | 170.61 | -175.04 |  |  |  |  |  |  |  |  |  |  |  |  |  |  |  |  |  |
| 66.87915 |  | 176.1749 | 164.3637 | -175.579 |  | 60.76 | 132.64 | 175.01 | -143.13 | -177.97 | -43.5 | 170.61 | -175.04 |  |  |  |  |  |  |  |  |  |  |  |  |  |  |  |  |  |
| 75.74519 |  | -177.151 | 163.6213 | 178.9253 |  | 60.76 | 132.64 | 175.01 | -143.13 | -177.97 | -43.5 | 170.61 | -175.04 |  |  |  |  |  |  |  |  |  |  |  |  |  |  |  |  |  |
| 62.29317 |  | 151.9507 | 174.5443 | -171.443 |  | 60.76 | 132.64 | 175.01 | -143.13 | -177.97 | -43.5 | 170.61 | -175.04 |  |  |  |  |  |  |  |  |  |  |  |  |  |  |  |  |  |
| 80.17129 |  | 163.745 | 158.8427 | -166.356 |  | 60.76 | 132.64 | 175.01 | -143.13 | -177.97 | -43.5 | 170.61 | -175.04 |  |  |  |  |  |  |  |  |  |  |  |  |  |  |  |  |  |
| 63.72564 |  | 158.6213 | 168.194 | -174.838 |  | 60.76 | 132.64 | 175.01 | -143.13 | -177.97 | -43.5 | 170.61 | -175.04 |  |  |  |  |  |  |  |  |  |  |  |  |  |  |  |  |  |
| 59.16823 |  | 171.468 | -173.25 | -173.902 |  | 60.76 | 132.64 | 175.01 | -143.13 | -177.97 | -43.5 | 170.61 | -175.04 |  |  |  |  |  |  |  |  |  |  |  |  |  |  |  |  |  |

**Figure S8. Torsions C-CA-CB-CG for Lysine 41 generated from 300ps of molecular dynamics.**

**Figure S9. Torsions CA-CB-CG-CD for Lysine 41 generated from 300ps of molecular dynamics.**

**Figure S10.** **Torsions CB-CG-CD-CE for Lysine 41 generated from 300ps of molecular dynamics.**

**Figure S11. Torsions CG-CD-CE-NZ for Lysine 41 generated from 300ps of molecular dynamics.**
